# Sex-specific role of myostatin signaling in neonatal muscle growth, denervation atrophy, and neuromuscular contractures

**DOI:** 10.7554/eLife.81121

**Published:** 2022-10-31

**Authors:** Marianne E Emmert, Parul Aggarwal, Kritton Shay-Winkler, Se-Jin Lee, Qingnian Goh, Roger Cornwall

**Affiliations:** 1 Department of Medical Sciences, University of Cincinnati College of Medicine Cincinnati United States; 2 Division of Orthopaedic Surgery, Cincinnati Children’s Hospital Medical Center Cincinnati United States; 3 The Jackson Laboratory Farmington United States; 4 Department of Genetics and Genome Sciences, University of Connecticut School of Medicine Farmington United States; 5 Department of Orthopaedic Surgery, University of Cincinnati College of Medicine Cincinnati United States; 6 Division of Developmental Biology, Cincinnati Children’s Hospital Medical Center Cincinnati United States; 7 Department of Pediatrics, University of Cincinnati College of Medicine Cincinnati United States; Icahn School of Medicine at Mount Sinai United States; Icahn School of Medicine at Mount Sinai United States

**Keywords:** sex dimorphisms, myostatin, neonatal denervation, neuromuscular contractures, muscle atrophy, muscle length, Mouse

## Abstract

Neonatal brachial plexus injury (NBPI) causes disabling and incurable muscle contractures that result from impaired longitudinal growth of denervated muscles. This deficit in muscle growth is driven by increased proteasome-mediated protein degradation, suggesting a dysregulation of muscle proteostasis. The myostatin (MSTN) pathway, a prominent muscle-specific regulator of proteostasis, is a putative signaling mechanism by which neonatal denervation could impair longitudinal muscle growth, and thus a potential target to prevent NBPI-induced contractures. Through a mouse model of NBPI, our present study revealed that pharmacologic inhibition of MSTN signaling induces hypertrophy, restores longitudinal growth, and prevents contractures in denervated muscles of female but not male mice, despite inducing hypertrophy of normally innervated muscles in both sexes. Additionally, the MSTN-dependent impairment of longitudinal muscle growth after NBPI in female mice is associated with perturbation of 20S proteasome activity, but not through alterations in canonical MSTN signaling pathways. These findings reveal a sex dimorphism in the regulation of neonatal longitudinal muscle growth and contractures, thereby providing insights into contracture pathophysiology, identifying a potential muscle-specific therapeutic target for contracture prevention, and underscoring the importance of sex as a biological variable in the pathophysiology of neuromuscular disorders.

## Introduction

Injury to the brachial plexus at birth (neonatal brachial plexus injury – NBPI) is the most common cause of upper limb paralysis in children (Foad et al., 2008), occurring in approximately 1.5 of every 1000 live births, in both females and males at an equal rate (McLaren et al., 2019). This initial nerve injury leads to permanent neurologic deficits in 20–40% of affected children (Govindan and Burrows, 2019; Pondaag et al., 2004), and results in the secondary formation of disabling and incurable muscle contractures, or joint stiffness. Contractures severely impede range of motion and functional use of the involved limbs, ultimately resulting in skeletal deformity that further worsens limb dysfunction (Hale et al., 2010). However, current strategies are insufficient in restoring muscle function and joint range of motion once contractures have developed, and may even worsen function by further weakening abnormal muscles (Newman et al., 2006; Pedowitz et al., 2007; van der Sluijs et al., 2004). To develop effective strategies for treating contractures, it is therefore important to establish greater insights into the pathophysiology of contracture formation.

Using a mouse model of NBPI, we previously discovered that contractures result from impaired longitudinal muscle growth, as characterized by the overstretch/elongation of sarcomeres in denervated muscles (Nikolaou et al., 2011; Weekley et al., 2012; Nikolaou et al., 2014; Nikolaou et al., 2015). Such deficits in muscle length are independent of satellite cell-mediated myonuclear accretion, but are instead caused by aberrant levels of muscle protein degradation associated with increased catalytic activity of the 20S proteasome (Nikolaou et al., 2019). These results posit a critical mechanistic role for proteasome-mediated dysregulation of muscle proteostasis in driving the impairment of longitudinal muscle growth that ultimately causes contractures. As proof of concept, we showed that inhibition of the proteasome with an FDA-approved proteasome inhibitor, bortezomib, restores sarcomere length and reduces contracture formation (Nikolaou et al., 2019). Our collective findings therefore establish that the biomechanical contracture phenotype can be attributed to a biological defect, and that contractures can be corrected by targeting their causative mechanism. However, despite the effectiveness of proteasome inhibition for preventing contractures, this pharmacologic strategy cannot be easily translated to children. We recently reported that bortezomib treatment is required throughout postnatal development to prevent contractures at skeletal maturity, and that its efficacy is diminished beyond the neonatal period (Goh et al., 2021). This need for chronic bortezomib treatment is problematic. Prolonged administration of proteasome inhibitors results in potential cumulative toxicity as these drugs nonspecifically block degradation and cause tissue damage to many organs, such as the brain (Nikolaou et al., 2019), and even impede musculoskeletal development (Goh et al., 2021). Due to these concerns, our laboratory seeks to identify safer strategies to prevent denervation-induced contractures by targeting skeletal muscle-specific regulators of proteostasis to reduce the elevated protein degradation responsible for contractures.

One such signaling mechanism specific to skeletal muscle is the myostatin (MSTN) pathway. MSTN, also known as growth differentiation factor-8 (GDF8), is a myokine and a member of the transforming growth factor-β (TGF-β) superfamily (Lee, 2004). In normally innervated skeletal muscles, ligand binding of MSTN to its Type 2 receptors, ACVR2 (ActRIIA) and ACVR2B (ActRIIB), activates the downstream Smad proteins 2 and 3 (Sartori et al., 2009; Trendelenburg et al., 2009). These signaling events regulate muscle homeostasis by increasing degradation and decreasing Akt/mTOR-mediated synthesis, ultimately attenuating muscle size and limiting aberrant growth (Lee, 2004; Lee, 2012; Lee, 2021). MSTN thereby functions as a negative regulator of muscle mass and protein balance. Given its prominent role in muscle proteostasis, it is possible that MSTN is a signaling pathway by which denervation impairs muscle growth. As mentioned, we previously discovered that neonatal denervation increases protein degradation in denervated muscles (Nikolaou et al., 2019), which leads to impaired longitudinal muscle growth that ultimately causes contractures (Nikolaou et al., 2011; Weekley et al., 2012; Nikolaou et al., 2014; Nikolaou et al., 2015). However, the mechanism by which neonatal denervation increases protein degradation is unknown. Given that MSTN is a potent inhibitor of muscle growth through its mediation of muscle proteostasis, we speculate that MSTN signaling may be a mechanistic pathway linking denervation with protein degradation, leading to impaired muscle growth and ultimately contractures. If contractures are indeed mediated through MSTN signaling, it could lead to a potential breakthrough in identifying a muscle-specific target for contracture prevention.

Hence, in the current study, we seek to elucidate the role of MSTN in the formation of denervation-induced muscle contractures. Using a soluble decoy receptor (ACVR2B-Fc) to inhibit ligand binding of MSTN to ACVR2B (Lee et al., 2005; Lee et al., 2012; Goh et al., 2019; Lee et al., 2020), we specifically investigated whether pharmacologic inhibition of MSTN signaling preserves longitudinal muscle growth and prevents contractures after NBPI. Our collective results establish several novel insights in skeletal muscle biology and contracture pathophysiology. First, we show that pharmacologic MSTN inhibition is efficacious in augmenting neonatal growth of normally innervated skeletal muscles in both female and male mice. Second, MSTN inhibition effectively restores sarcomere length and reduces contracture severity exclusively in neonatally denervated muscles of female mice, suggesting a sex-dependent role for the MSTN pathway in modulating longitudinal muscle growth and contracture formation. Curiously, MSTN inhibition improves muscle proteostasis in denervated female muscles by circumventing known downstream signaling pathways to directly target the 20S proteasome. These discoveries establish a critical link between denervation and impaired longitudinal muscle growth, which helps guide translation of safer strategies for preventing contractures. Furthermore, they highlight the importance of sex as a biological variable in disease pathology and treatment strategies, as well as in future studies dissecting the molecular regulation of longitudinal muscle growth.

## Results

### MSTN inhibition enhances neonatal skeletal muscle growth

**Figure 1.**
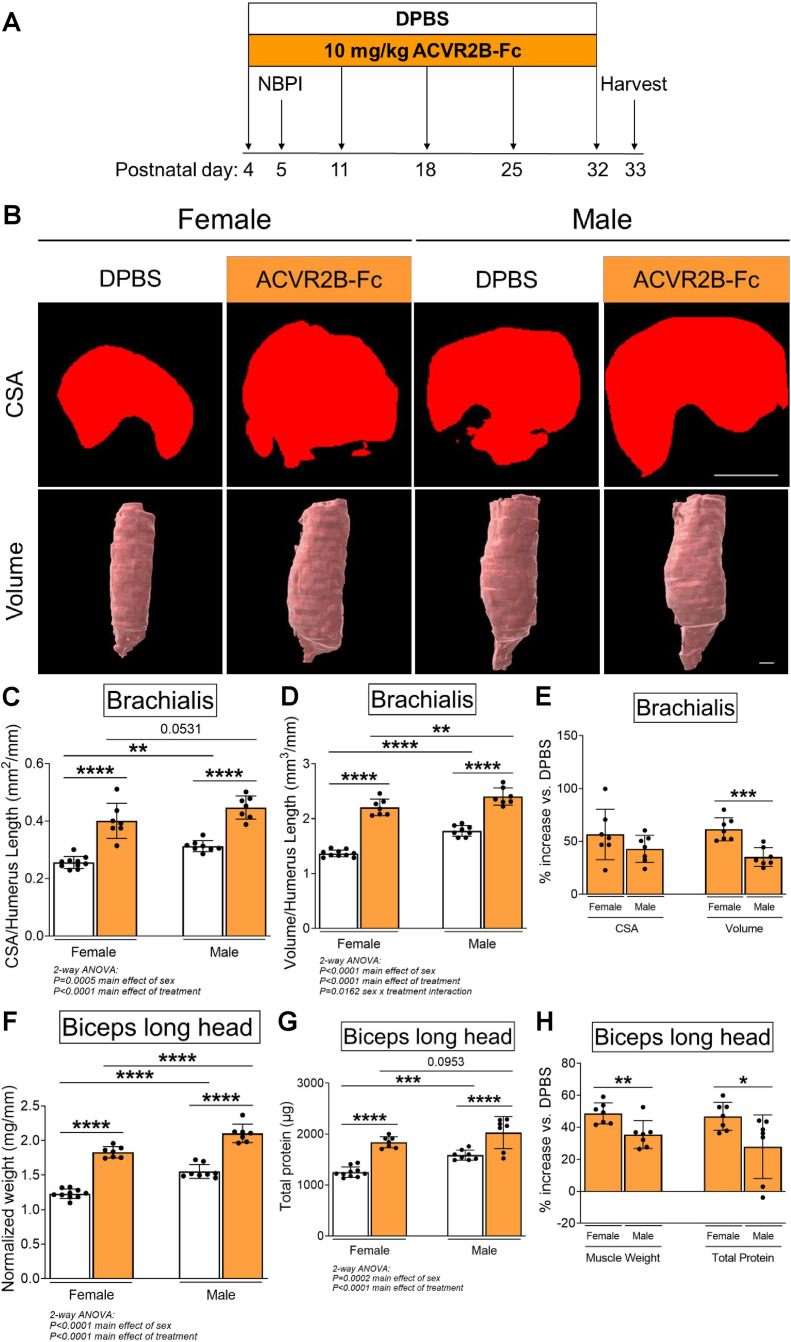
Pharmacologic inhibition of myostatin (MSTN) enhances neonatal skeletal muscle growth. (**A**) Schematic depiction of ACVR2B-Fc treatment to inhibit MSTN in neonatal mice prior to and after unilateral brachial plexus injury (NBPI) at P5. Representative MicroCT images in (**B**) transverse and three-dimensional views revealed increased growth of non-denervated brachialis muscles in both female and male mice following 4 weeks of pharmacologic MSTN inhibition with ACVR2B-Fc. Quantitative analyses of (**C**) cross-sectional area and (**D**) whole-muscle volume in control brachialis muscles confirmed that neonatal MSTN inhibition enhances skeletal muscle growth. Neonatal MSTN inhibition further enhances (**F**) muscle weight and (**G**) total protein content of non-denervated biceps muscles in both sexes. Despite this, the increases in (**E**) muscle volume, and (**H**) muscle weight and protein levels were larger in females than males when compared to their respective DPBS controls. Data are presented as mean ± standard deviation (SD), *n* = 7–10 independent mice. Statistical analyses: (**C**, **D**, **F**, **G**) two-way analysis of variance (ANOVA) for sex and treatment with a Bonferroni correction for multiple comparisons, (**E**, **H**) unpaired two-tailed Student’s *t*-tests. *p < 0.05, **p < 0.01, ***p < 0.001, ****p < 0.0001. Scale bar: 1000 µm.

While pharmacologic inhibition of MSTN signaling with the soluble ACVR2B-Fc decoy receptor has been shown to induce robust muscle growth in adult mice (Lee et al., 2005), it has not been validated in neonatal mice. To limit potential toxicity during the neonatal period, we standardized the frequency of ACVR2B-Fc treatment to weekly injections (Lee et al., 2005; Lee et al., 2012). We began by verifying the effects of this dosage on neonatal skeletal muscle growth (Figure 1A). To further account for sexual dimorphisms in muscle size during postnatal development (Griffin and Goldspink, 1973), we analyzed the effect of MSTN inhibition on developmental growth according to individual sexes. Our findings revealed that inhibition of the MSTN pathway resulted in robust growth of normally innervated forelimb muscles in both neonatal female and male mice. Specifically, MSTN inhibition enhanced cross-sectional and volumetric growth of the brachialis muscles (Figure 1B–D), as well as increased overall muscle weight and elevated total protein levels in biceps muscles (Figure 1F, G). There were no changes in protein levels normalized to muscle weight with ACVR2B-Fc treatment (Figure 1—figure supplement 1A). Despite this, the increases in brachialis volume (61% vs. 35%), biceps weight (49% vs. 35%), and biceps total protein content (47% vs. 28%) were greater in females than males when compared to respective controls (Figure 1E, H). These results indicate that while neonatal inhibition of MSTN signaling effectively enhances skeletal muscle growth in both sexes, it facilitates greater protein content and growth in female muscles. Curiously, ACVR2B-Fc treatment did not yield additional gains in body weight but instead attenuated humerus length and heart size in male mice, whereas female mice had smaller spleens after treatment (Figure 1—figure supplement 1B–E). Hence, while pharmacologic MSTN inhibition is associated with several off-target effects, these alterations manifest more prevalently in male tissues.

**Figure 1—figure supplement 1.**
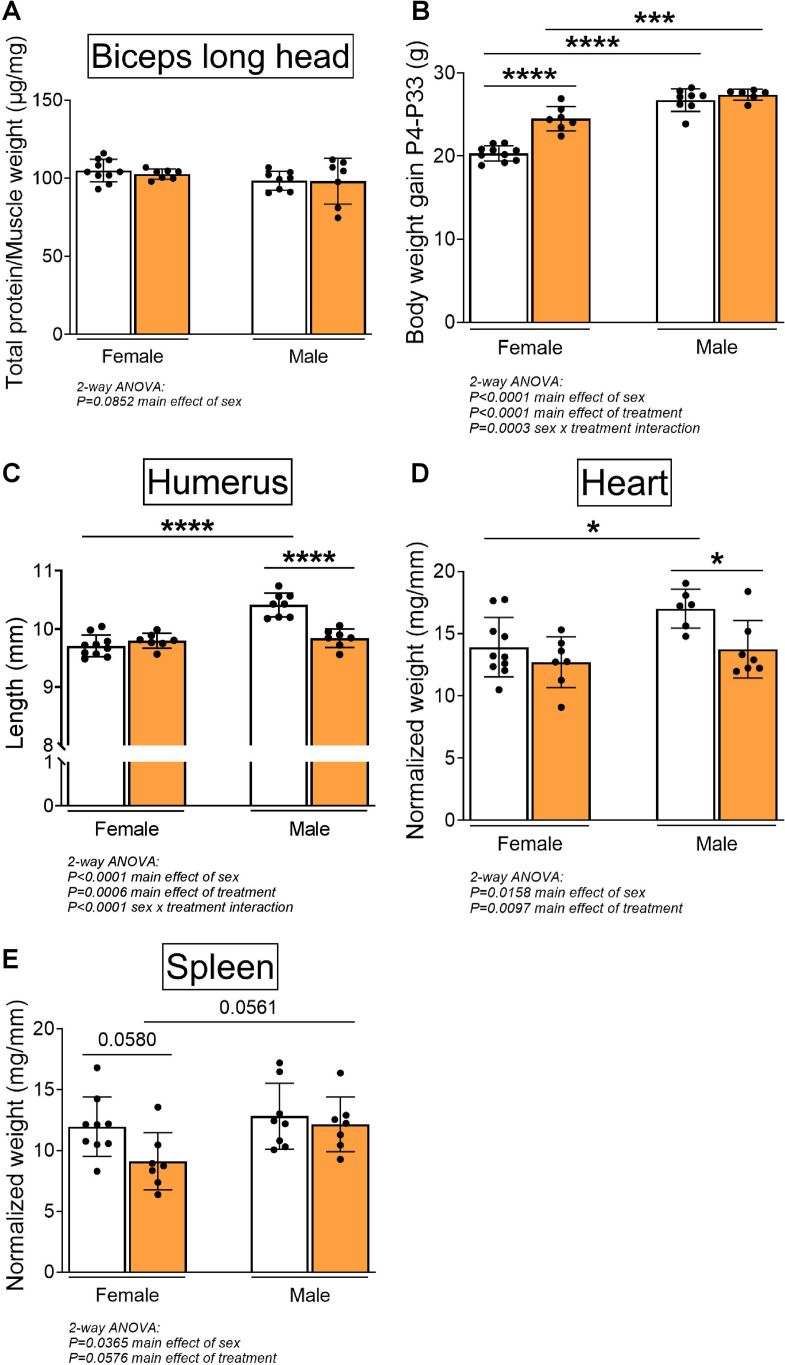
Off-target effects of pharmacologic myostatin (MSTN) inhibition. (**A**) MSTN inhibition does not alter protein levels in normally innervated biceps when normalized to muscle weight. Treatment with ACVR2B-Fc (**B**) does not promote additional weight gains in male mice, but instead, attenuates skeletal growth of non-denervated forelimbs and (**D**) reduces heart size. In contrast, ACVR2B-Fc treatment (**E**) reduces spleen size only in female mice. Data are presented as mean ± standard deviation (SD), *n* = 7–10 independent mice. Statistical analyses: (**A–E**) two-way analysis of variance (ANOVA) for sex and treatment with a Bonferroni correction for multiple comparisons. *p < 0.05, ***p < 0.001, ****p < 0.0001.

### MSTN signaling modulates denervation atrophy in a sex-specific manner

**Figure 2.**
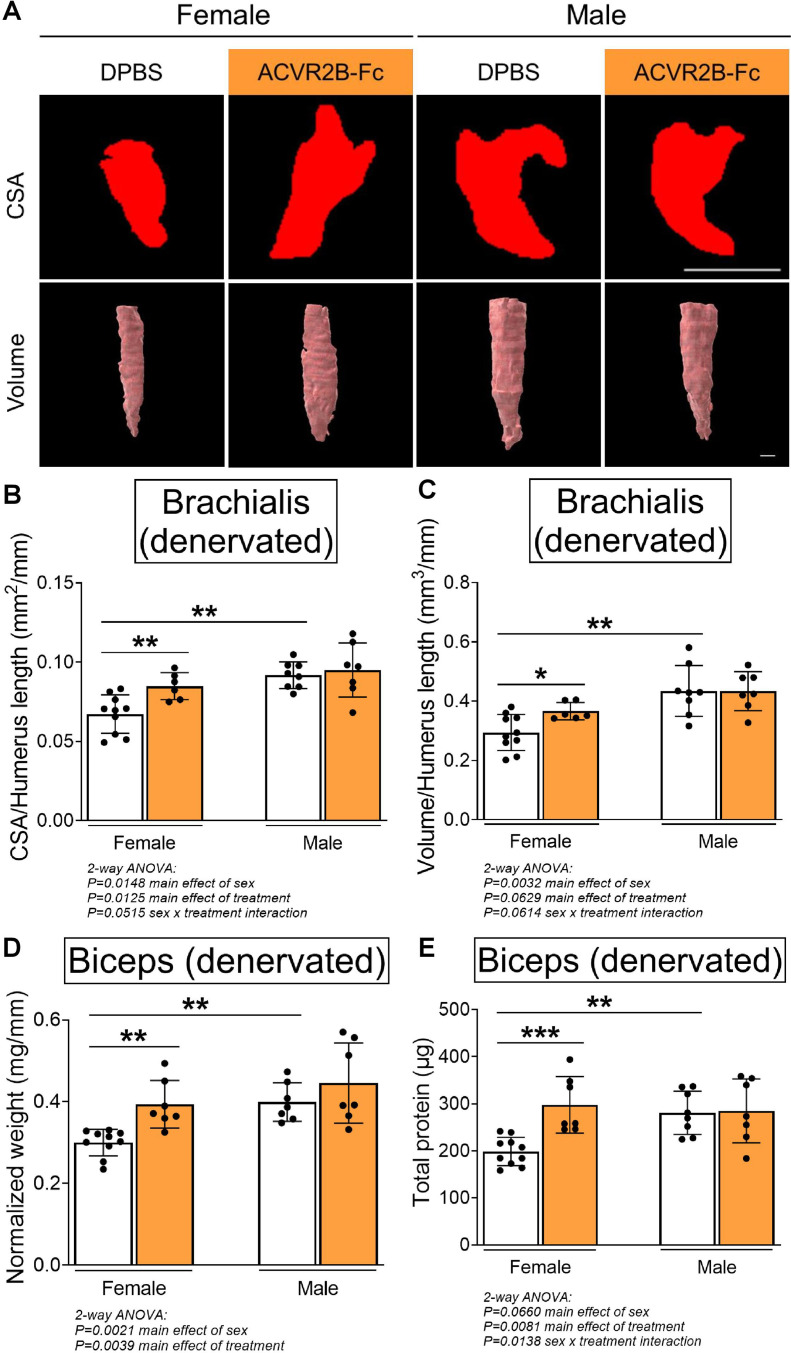
Pharmacologic myostatin (MSTN) inhibition reduces denervation-induced muscle atrophy in neonatal female mice. Representative MicroCT images in (**A**) transverse and three-dimensional views revealed larger denervated brachialis muscles in female mice following MSTN inhibition. Quantitative analyses of (**B**) brachialis cross-sectional area and (**C**) whole-muscle volume confirmed MSTN inhibition reduces denervation-induced atrophy only in female mice. This partial rescue in growth of the denervated brachialis muscles is accompanied by increased (**D**) muscle weight and (**E**) total protein content of denervated biceps muscles in female mice only. Data are presented as mean ± standard deviation (SD), *n* = 7–10 independent mice. Statistical analyses: (**B–E**) two-way analysis of variance (ANOVA) for sex and treatment with a Bonferroni correction for multiple comparisons. *p < 0.05, **p < 0.01, ***p < 0.001. Scale bar: 1000 µm.

To elucidate the contribution of the MSTN pathway in modulating contracture formation, we began by investigating the effects of ACVR2B-Fc treatment on neonatally denervated forelimb muscles. In contrast to normally innervated neonatal muscles, pharmacologic blockade of MSTN signaling reduced muscle loss of denervated brachialis and biceps muscles only in female mice (Figure 2A–E). In denervated female muscles, MSTN inhibition specifically increased brachialis cross-sectional area and volume by 25–26%, and biceps weight and total protein by 31–50%. However, there were no improvements with MSTN inhibition in denervated male muscles, as ACVR2B-Fc treatment failed to increase brachialis muscle CSA and volume (Figure 2A–C), or biceps muscle weight and total protein levels (Figure 2D, E) in male mice. Additionally, while MSTN inhibition did not elevate protein levels normalized to muscle weight in denervated muscles beyond the respective sex-specific controls, it led to higher levels in female than male muscles (Figure 2—figure supplement 1A). Similar to the contralateral forelimbs, MSTN inhibition in male mice resulted in an attenuation of skeletal growth in the denervated humerus (Figure 2—figure supplement 1B). These parallel observations suggest that ACVR2B-Fc attenuates neonatal skeletal growth exclusively in male mice. Taken together, our results illustrate that while neonatal MSTN inhibition facilitates developmental growth of normally innervated muscles in both males and females, it promotes neonatal growth and reduces atrophy of denervated muscles exclusively in females.

**Figure 2—figure supplement 1.**
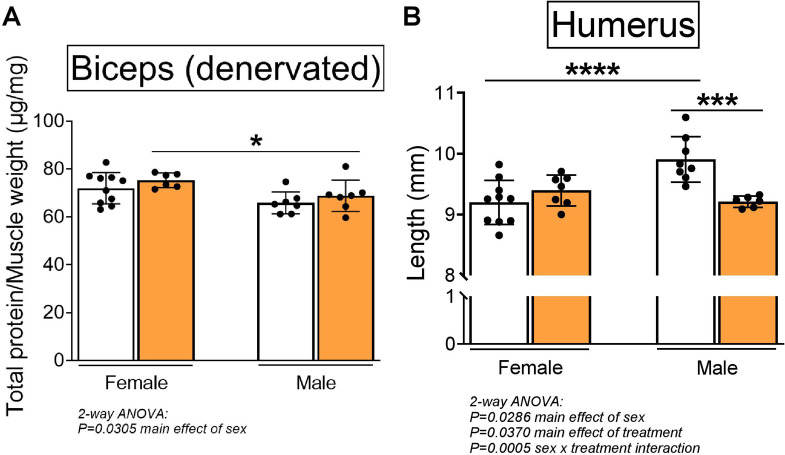
Effect of pharmacologic myostatin (MSTN) inhibition on normalized protein levels and skeletal growth in denervated forelimb. (**A**) MSTN inhibition does not alter protein levels in denervated biceps when normalized to muscle weight. (**B**) Treatment with ACVR2B-Fc attenuates skeletal growth of denervated forelimbs only in male mice. Data are presented as mean ± standard deviation (SD), *n* = 7–10 independent mice. Statistical analyses: (**A**,** B**) two-way analysis of variance (ANOVA) for sex and treatment with a Bonferroni correction for multiple comparisons. *p < 0.05, ***p < 0.001, ****p < 0.0001.

### MSTN signaling modulates contracture formation in a sex-specific manner

**Figure 3.**
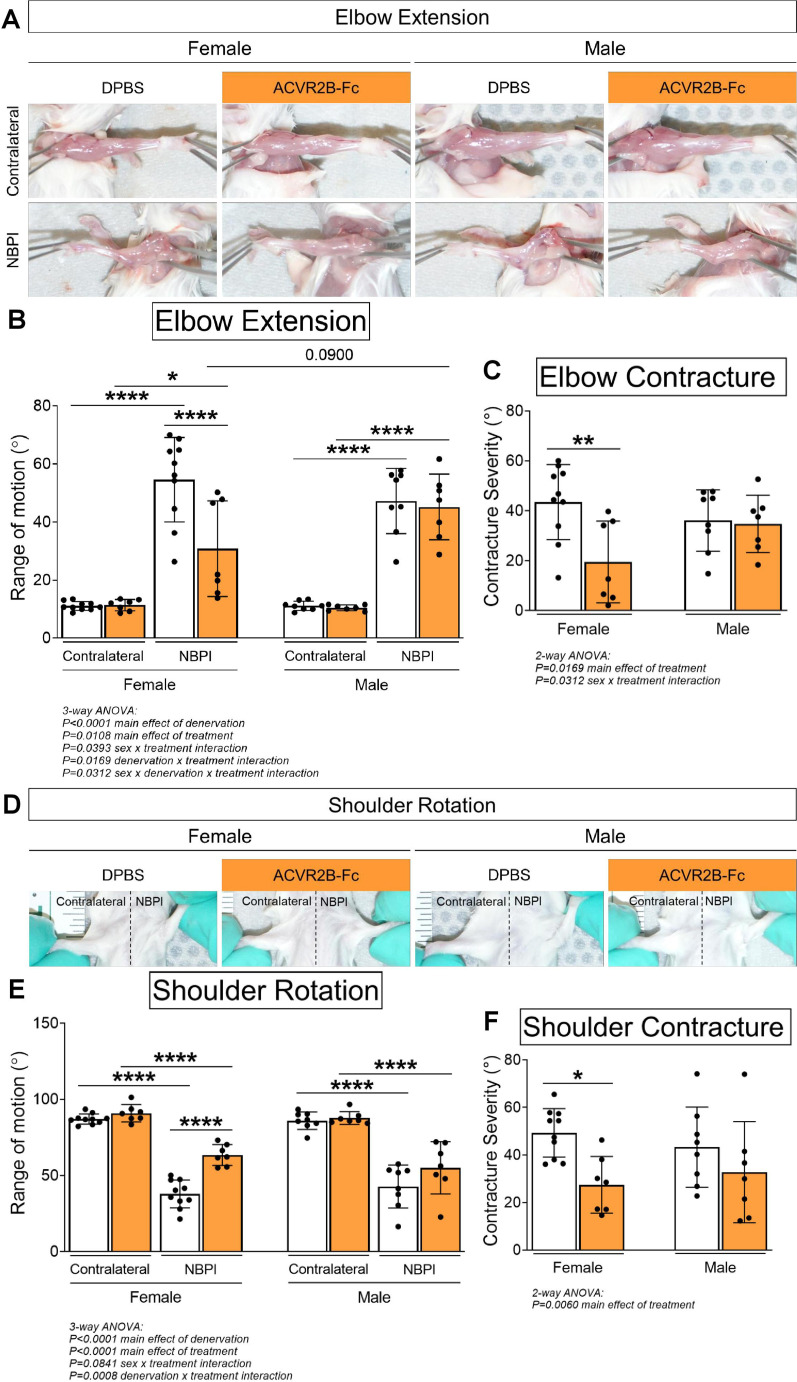
Pharmacologic myostatin (MSTN) inhibition reduces neonatal denervation-induced contractures in neonatal female mice. (**A**) Representative images of denervated (neonatal brachial plexus injury, NBPI) and contralateral forelimbs, and quantitation of both (**B**) elbow joint extension range of motion and (**C**) contracture severity revealed that MSTN inhibition reduces the formation of elbow flexion contractures after 4 weeks of neonatal denervation in female, but not male mice. Similarly, (**D**) representative images of bilateral forelimbs, and quantitation of both (**E**) shoulder joint external rotation range of motion and (**F**) contracture severity demonstrated improvements in shoulder rotation only in female mice. In (**C, F**), elbow and shoulder contracture severity is calculated as the difference in passive elbow extension and shoulder rotation, respectively, between the NBPI side and the contralateral side. Data are presented as mean ± standard deviation (SD), *n* = 7–10 independent mice. Statistical analyses: (**B**, **E**) three-way analysis of variance (ANOVA) for sex, treatment, and denervation (repeated measures between forelimbs) with a Bonferroni correction for multiple comparisons, (**C**, **F**) two-way ANOVA for sex and treatment with a Bonferroni correction for multiple comparisons. *p < 0.05, **p < 0.01, ****p < 0.0001.

**Figure 4.**
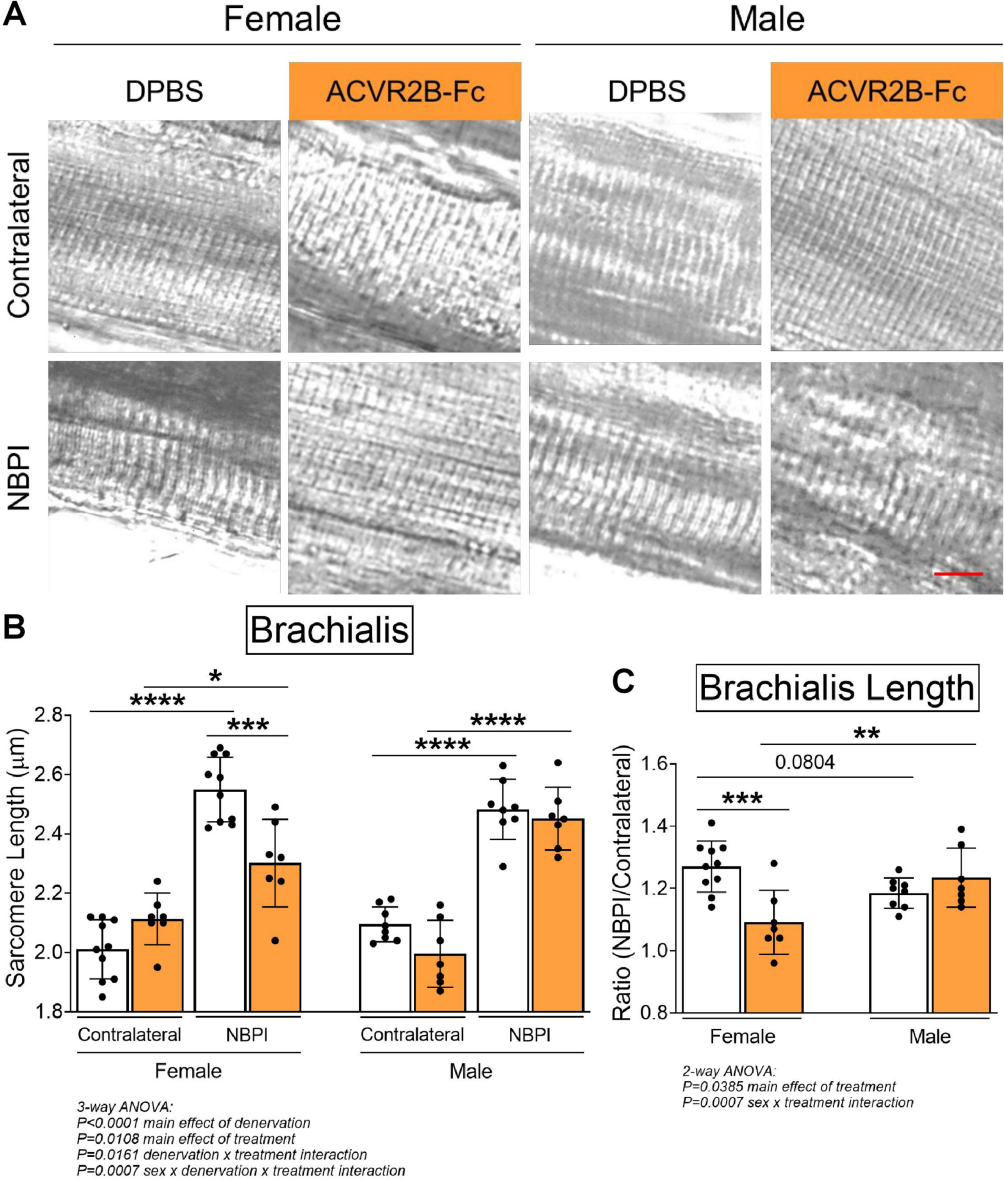
Pharmacologic myostatin (MSTN) inhibition preserves longitudinal muscle growth of denervated muscles in neonatal female mice. (**A**) Representative differential interference contrast (DIC) images of sarcomeres, and (**B**) quantitation of sarcomere length showed that MSTN inhibition preserves sarcomere length of denervated muscles after neonatal denervation in female mice, whereas the sarcomeres of denervated muscles in male mice remain overstretched. (**C**) Sarcomere length on the neonatal brachial plexus injury (NBPI) side is normalized to the contralateral side to generate a sarcomere length ratio. This normalized sarcomere length between control and NBPI limbs further confirmed that MSTN inhibition improves functional length of denervated muscles only in female mice. Data are presented as mean ± standard deviation (SD), *n* = 7–10 independent mice. Statistical analyses: (**B**) three-way analysis of variance (ANOVA) for sex, treatment, and denervation (repeated measures between forelimbs) with a Bonferroni correction for multiple comparisons, (**C**) two-way ANOVA for sex and treatment with a Bonferroni correction for multiple comparisons. *p < 0.05, **p < 0.01, ***p < 0.001, ****p < 0.0001. Scale bar: 10 µm.

Due to the differential effects in denervated muscle growth, we speculated that the MSTN signaling pathway may mediate contracture formation in a sex-dependent manner. Indeed, we discovered that ACVR2B-Fc treatment improved passive range of motion in both elbow and shoulder joints only in female mice 4 weeks post-NBPI (Figure 3A, B, D, E). Close scrutiny of the elbow extension data revealed two populations of female NBPI mice with MSTN inhibition (Figure 3B). To account for this lack of normal distribution, additional analysis was performed in female mice using nonparametric tests, which corroborated that ACVR2B-Fc treatment improved elbow extension in denervated forelimbs (Figure 3—figure supplement 1A). This sex-specific improvement in joint mobility corresponded to a reduction in both elbow flexion and shoulder rotation contracture severity in female, but not male mice (Figure 3C, F). These results indicate a critical therapeutic window for MSTN inhibition in preventing contracture formation, and importantly, illuminate a sex-specific role for the MSTN pathway in mediating contracture formation. To gain further insights on this sex dimorphism, we next assessed the role of MSTN signaling on longitudinal growth of denervated muscles. Here, we observed that neonatal MSTN inhibition rescued sarcomere length (reduced sarcomere elongation) in the denervated brachialis muscles of female mice (Figure 4A–C), indicating an improvement in functional muscle length (Goh et al., 2021). Conversely, sarcomeres in the denervated brachialis of male mice remained overstretched, an indication of impaired longitudinal muscle growth. Hence, our collective findings establish that contracture formation following neonatal muscle denervation in female mice is driven by MSTN-dependent impairment of longitudinal muscle growth.

**Figure 3—figure supplement 1.**
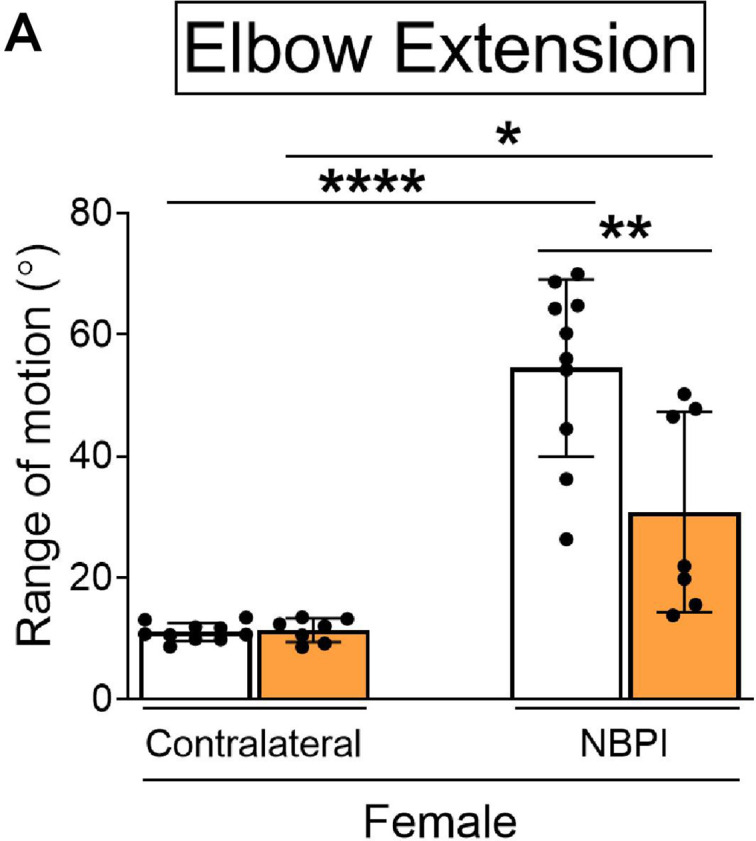
Nonparametric analysis in female mice. (**A**) Quantitation of elbow joint extension range of motion in female mice only. Data are presented as mean ± standard deviation (SD), *n* = 7–10 independent mice. Statistical analyses: unpaired, Mann–Whitney *U*-tests between groups and paired, Wilcoxon signed rank tests between limbs of mice in each group. *p < 0.05, **p < 0.01, ****p < 0.0001.

### MSTN mediates denervation-induced proteostasis dysregulation in a sex-specific manner

**Figure 5.**
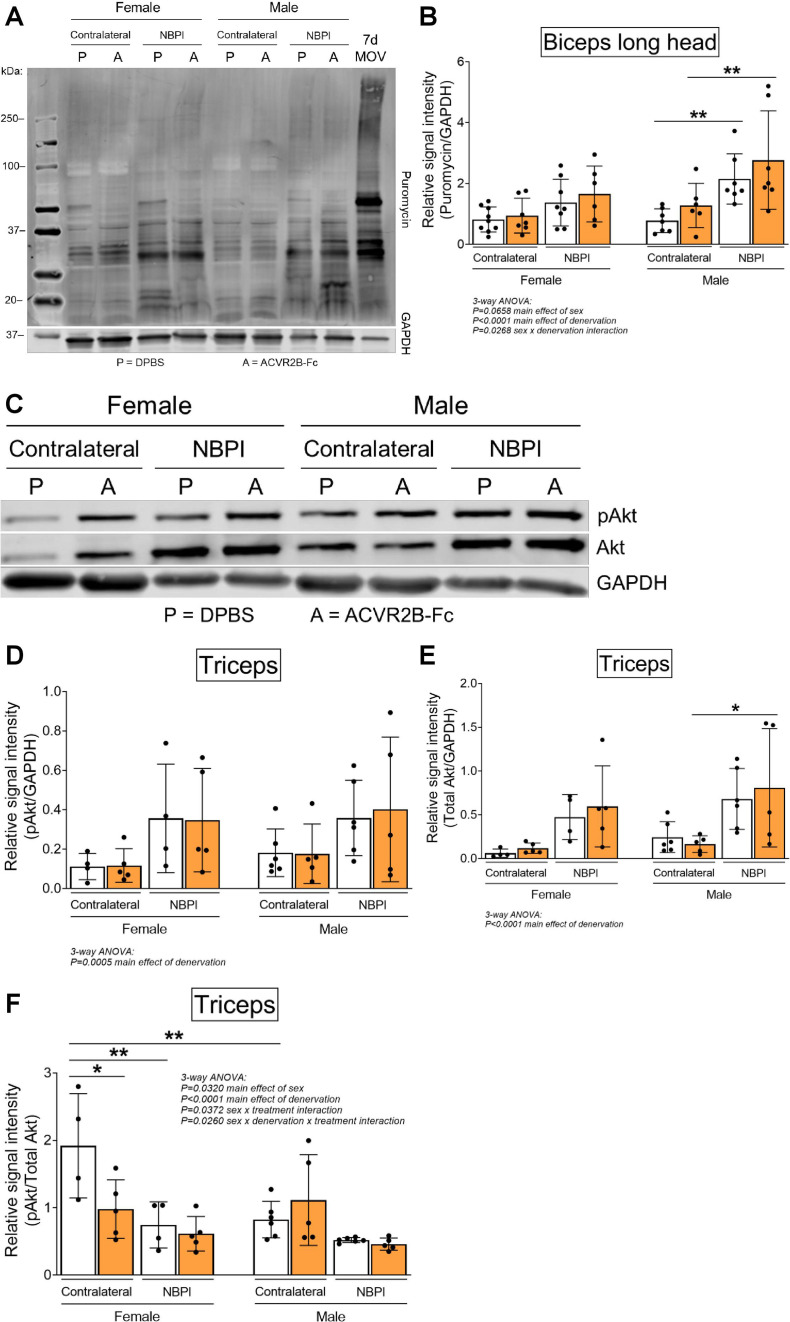
Sex-specific differences in myostatin (MSTN)-dependent contracture formation is not mediated through protein synthesis. (**A**) Representative western blots of puromycin incorporation and (**B**) quantitative analysis of optical densities revealed MSTN inhibition does not further alter the denervation-induced increases in whole-muscle protein synthesis in biceps muscles of both female and male mice. *n* = 7–10 independent mice. (**C**) Representative western blots and quantitative analyses of (**D**) pAkt (Ser473) and (**E**) total Akt similarly showed that ACVR2B-Fc treatment does not lead to additional increases in activity and translation of Akt after denervation in both sexes. (**F**) Quantification of the western signal for pAkt normalized to total protein levels further indicated that MSTN inhibition does not alter Akt/mTOR signaling in neonatally denervated muscles. *n* = 4–6 independent mice. Statistical analyses: (**B**, **D–F**) three-way analysis of variance (ANOVA) for sex, treatment, and denervation (repeated measures between forelimbs) with a Bonferroni correction for multiple comparisons. *p < 0.05, **p < 0.01. 7d MOV = adult mouse plantaris muscle that had been subjected to 7 days of mechanical overload. Figure 5—source data 1.Original and uncropped gels for Puromycin (Figure 5A). Figure 5—source data 2.Original and uncropped gels for GAPDH (Figure 5A). Figure 5—source data 3.Original and uncropped gels for pAkt (Figure 5C). Figure 5—source data 4.Original and uncropped gels for Akt (Figure 5C). Figure 5—source data 5.Original and uncropped gels for GAPDH (Figure 5C).

Since our prior work revealed that impaired longitudinal muscle growth following neonatal denervation is driven by increased levels of proteasome-mediated protein degradation (Nikolaou et al., 2019), we subsequently explored the role of MSTN signaling in proteostasis dysregulation to decipher mechanisms governing the observed sex dimorphisms. We first assessed whether MSTN inhibition is able to further increase the elevated levels of protein synthesis in neonatally denervated muscles (Nikolaou et al., 2019). Through western blot analysis of puromycin incorporation (Goodman et al., 2011; Schmidt et al., 2009), we discovered that while 4 weeks of NBPI drove protein synthesis in denervated biceps muscles, MSTN inhibition does not augment this increase in either sex (Figure 5A, B). This finding was corroborated by western blot analyses of the Akt/mTOR hypertrophic signaling pathway (Bodine et al., 2001; Rommel et al., 2001; Egerman and Glass, 2014; Sartori et al., 2021), which revealed that perturbations in Akt activity and total protein expression in denervated triceps muscles were not further impacted by ACVR2B-Fc treatment at 4 weeks post-NBPI (Figure 5C–F). These collective findings therefore indicate that MSTN-mediated proteostasis dysregulation in denervated female muscles occurs independent of Akt/mTOR-mediated protein synthesis.

**Figure 6.**
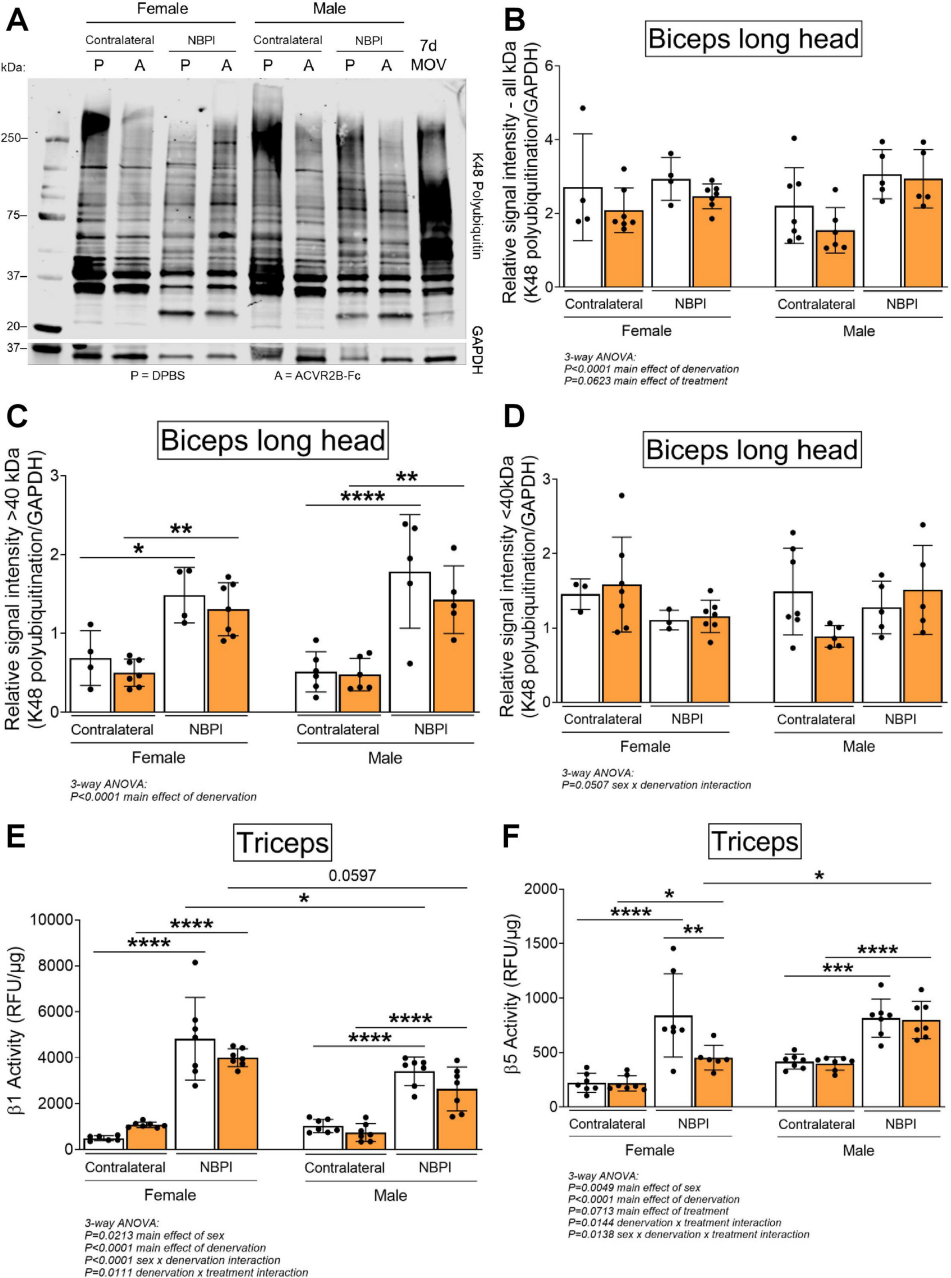
Sex-specific differences in myostatin (MSTN)-dependent contracture formation is mediated through proteasome activity. (**A**) Representative western blots of K48-linked polyubiquitin and (**B**) quantitative analysis of optical densities showed similar levels of ubiquitination in control and denervated biceps muscles of neonatal female and male mice. Despite this, (**C**) in-depth analyses of optical densities discovered that denervation increases ubiquitination levels of higher molecular weight proteins >40 kDa, (**D**) but not lower molecular proteins <40 kDa in both sexes. Importantly, MSTN inhibition does not alter levels of K48 polyubiquitination across the different molecular weights after denervation. *n* = 4–7 independent mice. (**E**, **F**) Assessment of proteasome activity in triceps muscles revealed that MSTN inhibition blunts the denervation-induced increase in β5 but not β1 constitutive proteasome activity solely in female mice. *n* = 7 independent mice. Data are presented as mean ± standard deviation (SD). Statistical analyses: (**B–F**) three-way analysis of variance (ANOVA) for sex, treatment, and denervation (repeated measures between forelimbs) with a Bonferroni correction for multiple comparisons. *p < 0.05, **p < 0.01, ***p < 0.001, ****p < 0.0001. 7d MOV = adult mouse plantaris muscle that had been subjected to 7 days of mechanical overload. Figure 6—source data 1.Original and uncropped gels for K48 Polyubiquitin (Figure 6A). Figure 6—source data 2.Original and uncropped gels for GAPDH (Figure 6A).

**Figure 7.**
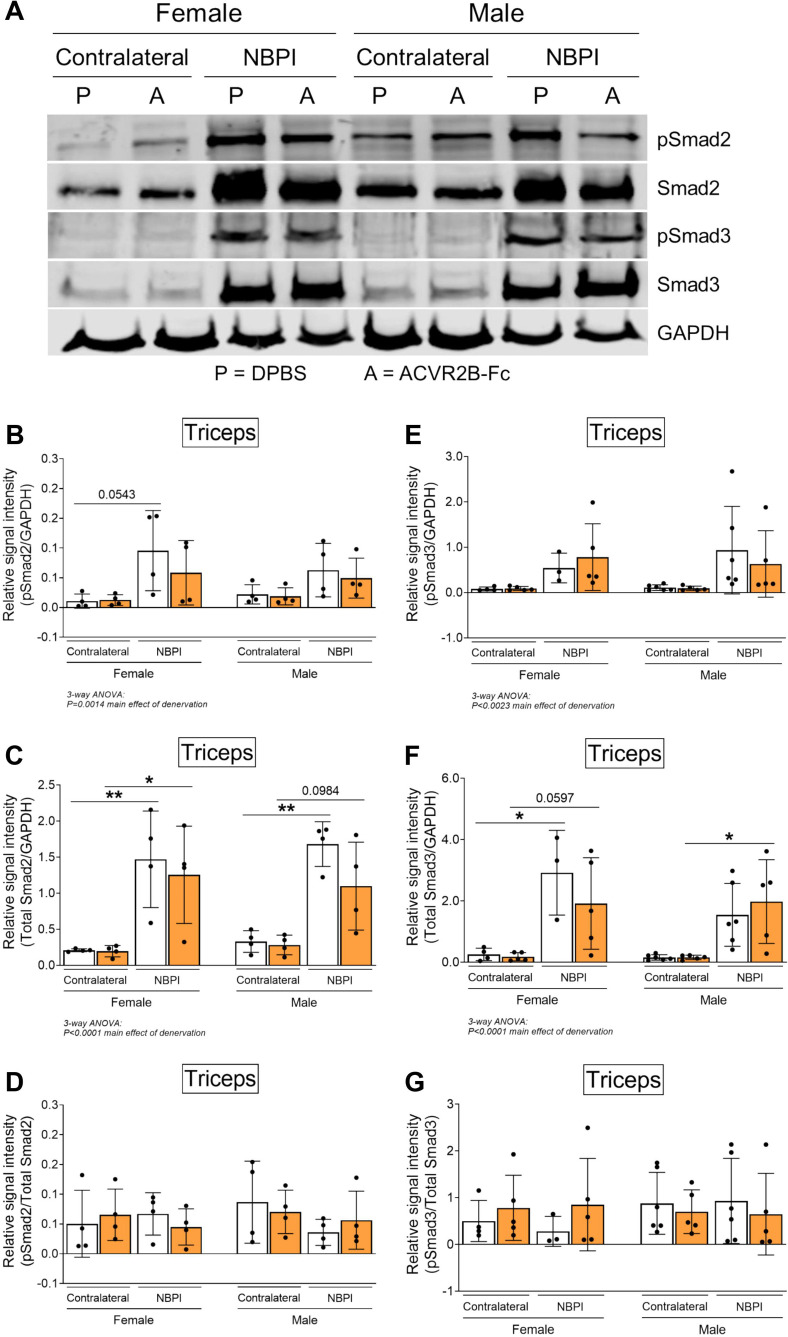
Sex-specific differences in myostatin (MSTN)-mediated proteostasis dysregulation occurs independent of Smad2/3 signaling. (**A**) Representative western blots and quantitative analyses of (**B**) pSmad2, (**C**) total Smad2, (**E**) pSmad3, and (**F**) total Smad3 revealed that ACVR2B-Fc treatment does not blunt the denervation-induced increase in activity and translation of Smad2 and Smad3 in triceps muscles of both sexes. Quantification of the western signal for (**D**) pSmad2 and (**G**) pSmad3 normalized to total protein levels further indicated that MSTN inhibition does not alter Smad2/3 signaling in neonatally denervated muscles. *n* = 3–6 independent mice. Data are presented as mean ± standard deviation (SD). Statistical analyses: (**B–G**) three-way analysis of variance (ANOVA) for sex, treatment, and denervation (repeated measures between forelimbs) with a Bonferroni correction for multiple comparisons. *p < 0.05, **p < 0.01. Figure 7—source data 1.Original and uncropped gels for pSmad2 (Figure 7A). Figure 7—source data 2.Original and uncropped gels for Smad2 (Figure 7A). Figure 7—source data 3.Original and uncropped gels for pSmad3 (Figure 7A). Figure 7—source data 4.Original and uncropped gels for Smad3 (Figure 7A). Figure 7—source data 5.Original and uncropped gels for GAPDH (Figure 7A).

Having ruled out synthesis as an underlying mechanism for the observed sex dimorphisms, we next investigated the role of degradation, the other side of proteostasis. While total levels of K48-linked polyubiquitin were not different between normally innervated and denervated biceps muscles, we observed increased polyubiquitination of proteins greater than 40 kDa (Figure 6A–D), similar to what we have reported before (Nikolaou et al., 2019). However, MSTN inhibition did not attenuate the denervation-induced increases in proteins tagged for degradation at this molecular weight range in either sex. Since MSTN is known to activate the ubiquitin–proteasome pathway by upregulating the expression of upstream ubiquitin ligases (McFarlane et al., 2006; Lokireddy et al., 2011), it was surprising that its inhibition did not reduce ubiquitination levels. Further investigation revealed that MSTN inhibition did not alter protein levels of the muscle-specific E3 ligases, MuRF1 and Atrogin-1, (Figure 6—figure supplement 1A–C). Unexpectedly, denervation decreased MuRF1 and did not change Atrogin-1 levels at 4 weeks post-NBPI, which differ from our earlier reports of increased transcriptional activity of MuRF1 at 2 weeks post-NBPI (Nikolaou et al., 2019). While there is a need to better characterize the precise kinetics of these upstream ubiquitin ligases over time following denervation, we show that downstream levels of ubiquitination are ultimately elevated with denervation. The relationship between ubiquitin ligase expression, ubiquitination, and proteasome activity in neonatally denervated muscle must be more extensively investigated. Instead, ACVR2B-Fc effectively reduced the catalytic activity of the 20S proteasome β5 subunit in denervated triceps muscles only in female mice (Figure 6E, F). As K48-linked ubiquitin chains are the most prevalent signal that mark protein substrates for proteasome degradation (Yau and Rape, 2016), these findings suggest that MSTN regulates sex-specific proteostasis dysregulation by circumventing polyubiquitination and directly targeting the proteasome. To further decipher signaling mechanisms governing the sex differences in MSTN-mediated protein degradation, we characterized the Smad2/3 pathway in triceps muscles (Egerman and Glass, 2014; Sartori et al., 2021; Chen et al., 2017; Goodman et al., 2013). Here, we observed sex-independent increases in both Smad2 and Smad3 phosphorylation and translation with neonatal denervation, which were not further altered with MSTN inhibition (Figure 7A–G). Our collective findings thus posit a sex-specific role for MSTN in muscle proteostasis dysregulation after neonatal denervation through non-canonical signaling pathways.

**Figure 6—figure supplement 1.**
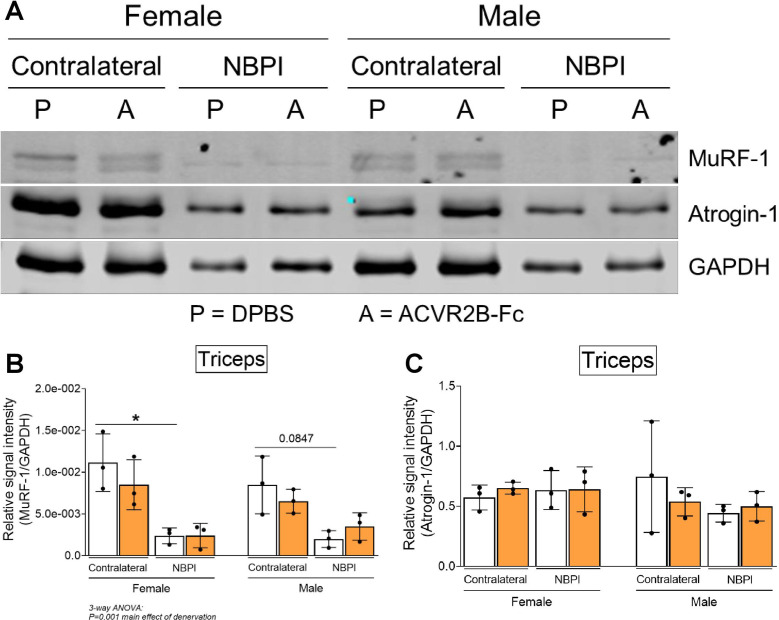
Assessment of muscle-specific ubiquitin ligases. (**A**) Representative western blots and quantitative analyses of (**B**) MuRF1, and (**C**) Atrogin-1 showed myostatin (MSTN) inhibition does not alter protein levels in denervated (neonatal brachial plexus injury, NBPI) or contralateral triceps muscles. Data are presented as mean ± standard deviation (SD), *n* = 3 independent mice. (**B**, **C**) Three-way analysis of variance (ANOVA) for sex, treatment, and denervation (repeated measures between forelimbs) with a Bonferroni correction for multiple comparisons. *p < 0.05. Figure 6—figure supplement 1—source data 1.Original and uncropped gels for MuRF1 (Figure 6—figure supplement 1A). Figure 6—figure supplement 1—source data 2.Original and uncropped gels for Atrogin-1 (Figure 6—figure supplement 1A). Figure 6—figure supplement 1—source data 3.Original and uncropped gels for GAPDH (Figure 6—figure supplement 1A).

### Less pronounced sex differences with proteasome inhibition

**Figure 8.**
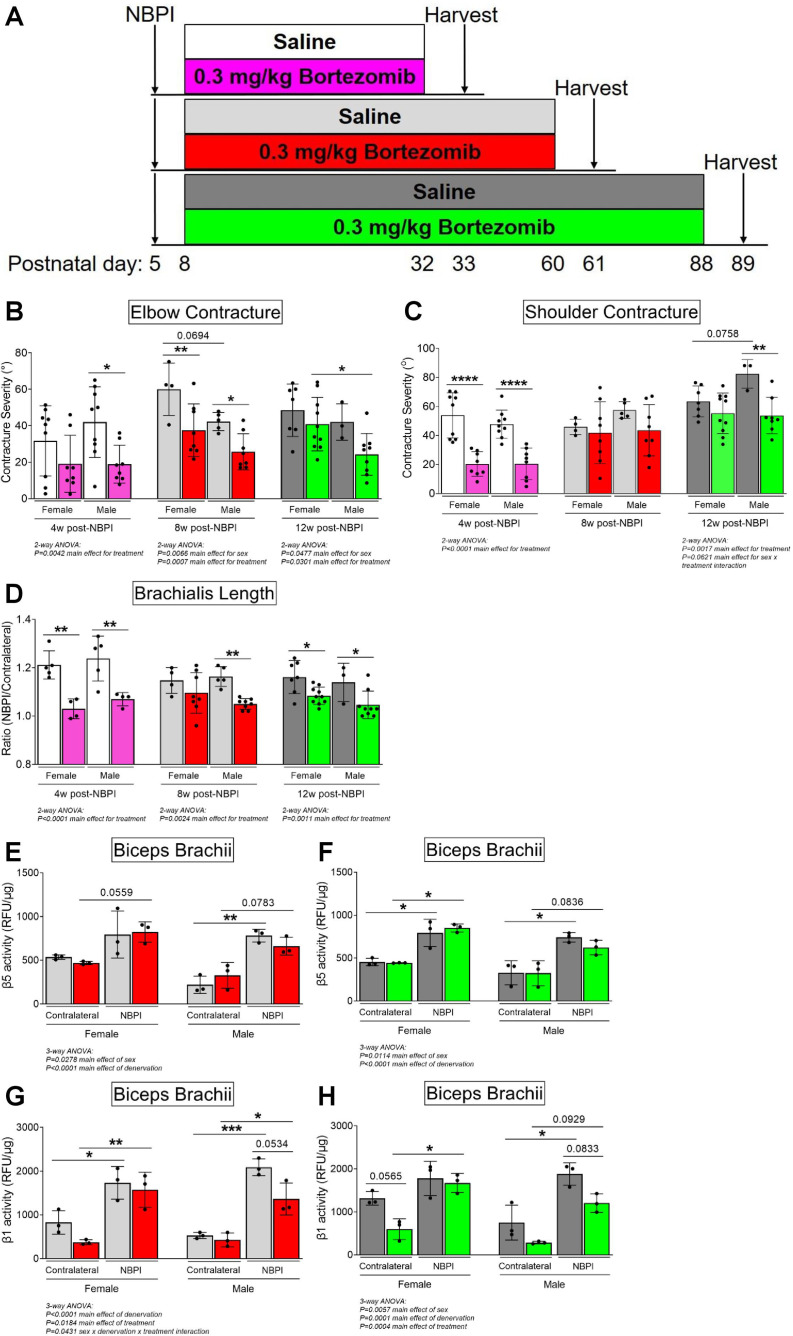
Proteasome inhibition with bortezomib prevents contractures and restores proteostasis preferentially in denervated muscles of neonatal male mice. (**A**) Schematic depiction of bortezomib treatment and assessment of outcomes at 4, 8, or 12 weeks post-neonatal brachial plexus injury (NBPI). (**B**, **C**) Continuous bortezomib treatment preferentially prevents elbow and shoulder contractures in male mice throughout postnatal development and into skeletal maturity (*n* = 3–10 independent mice). (**D**) Despite this, long-term bortezomib does not confer sex-specific improvements in longitudinal muscle growth, as sarcomere length was preserved in denervated brachialis muscles of both female and male mice (*n* = 3–10 independent mice). (**E–H**) As opposed to myostatin (MSTN) inhibition, the rescue in long-term contractures with continuous bortezomib treatment in male mice at 8 and 12 weeks post-NBPI is associated with a decrease in β1 proteasome subunit activity, rather than β5 activity of denervated biceps muscles (*n* = 3 independent mice). In (**B, C**), elbow and shoulder contracture severity is calculated as the difference in passive elbow extension and shoulder rotation, respectively, between the denervated (NBPI) side and the contralateral control side. In (**D**), sarcomere length on the NBPI side is normalized to the contralateral side to generate a sarcomere length ratio. Data are presented as mean ± standard deviation (SD). Statistical analyses: (**B–D**) two-way analysis of variance (ANOVA) for sex and treatment with a Bonferroni correction for multiple comparisons at each time point, (**E–H**) three-way ANOVA for sex, treatment, and denervation (repeated measures between forelimbs) with a Bonferroni correction for multiple comparisons. *p < 0.05, **p < 0.01, ***p < 0.001, ****p < 0.0001. This figure is generated from primary data reported in Goh et al., 2021.

Having observed a role for sex in MSTN-dependent contracture formation, we revisited our prior findings of long-term proteasome inhibition in preventing contractures (Goh et al., 2021). Here, we reanalyzed our data from 4, 8, and 12 w of continuous bortezomib treatment (Figure 8A) according to sex. In contrast with MSTN inhibition, we observed greater and more consistent reductions in elbow and shoulder contracture severity across the different time points in male mice with bortezomib treatment (Figure 8B, C). Sex differences in sarcomere length were less apparent, as bortezomib improved longitudinal muscle growth in female and male mice, except at 8 w of treatment (Figure 8D). Lastly, while bortezomib did not alter β5 subunit activity in either sex (Figure 8E, F), it attenuated β1 subunit activity at 8 and 12 w only in male mice (Figure 8G, H). Our prior reports of reduced β1 activity with bortezomib treatment at 4 w were observed only in female mice, as proteasome activity was not measured in male mice (data not shown) (Goh et al., 2021). Overall, while sex differences do exist with neonatal and long-term bortezomib treatment, these differences are more subtle than sex dimorphisms observed with MSTN inhibition. Regardless, they further highlight that sex mediates neonatally induced contractures through divergent pathways.

## Discussion

Secondary muscle contractures in pediatric neuromuscular disorders such as NBPI and cerebral palsy are major drivers of joint immobility, limb deformity, and physical dysfunction and disability (Hale et al., 2010). As existing treatments for contractures have focused on palliative mechanical solutions and do not address the underlying pathophysiology of contracture formation, the effectiveness of these strategies in restoring physical function is limited (Newman et al., 2006; Pedowitz et al., 2007; van der Sluijs et al., 2004). Specifically, the paucity in our understanding of contracture pathophysiology impedes clinical efforts to prevent contractures. To overcome these limitations, we have previously identified the dysregulation of muscle proteostasis, as characterized by an increase in proteasome-mediated protein degradation, as a causative mechanism of impaired longitudinal muscle growth and contracture formation following NBPI (Nikolaou et al., 2019). This discovery therefore highlights the possibility of pharmacologic strategies to prevent contractures through targeting of a biological mechanism. As our recent findings revealed a need for chronic pharmacologic proteasome inhibition with the potential for cumulative off-target toxicity (Goh et al., 2021), we must continue to identify safe and efficacious strategies for preventing pediatric muscle contractures by targeting muscle-specific regulators of proteostasis.

In our present study, we pharmacologically targeted MSTN, a prominent muscle-specific negative regulator of proteostasis, and identified several key findings regarding MSTN signaling in muscle growth and contracture pathophysiology. First, we discovered that pharmacologic MSTN inhibition with ACVR2B-Fc enhanced the size and protein content of normally innervated forelimb muscles in 1-month-old mice at P33, which is equivalent to the completion of neonatal muscle development in human infants (Dutta and Sengupta, 2016). In comparison, the youngest age that prior studies on MSTN inhibition have reported gains in lean body mass was in 2-month-old mice (McPherron and Lee, 2002). Our current finding therefore indicates a key regulatory role for MSTN signaling in governing muscle growth during the neonatal window, an often overlooked period in developmental muscle biology. This discovery not only reveals greater insights in skeletal muscle development, but also offers potential therapeutic strategies for overcoming low muscle mass arising from a host of genetic, metabolic, and hormonal disorders in the pediatric population (Orsso et al., 2019). In addition, MSTN inhibition also protected against atrophy and sarcomere overstretch in neonatally denervated forelimb muscles, as well as prevented contractures in female but not male mice. These findings therefore establish a sex-specific role for MSTN as a driver of impaired muscle growth and contracture formation exclusively in female mice. Critically, this sex dimorphism lies in the pathophysiology of denervation-induced atrophy and contractures rather than drug pharmacokinetics or pharmacodynamics, given that MSTN inhibition promotes robust growth in normally innervated muscles of both female and male mice. Indeed, MSTN inhibition restored proteostasis only through the attenuation of 20S proteasomal subunit activity in denervated muscles of female mice, without associated changes in overall levels of protein synthesis or K48-linked polyubiquitin. This postulates an intriguing mechanism for MSTN-dependent impairment of longitudinal muscle growth, whereby MSTN putatively circumvents canonical signaling pathways of proteostasis to directly regulate the 20S proteasome after neonatal muscle denervation. Beyond offering potential translational opportunities for female NBPI patients, these seminal findings highlight the underappreciated role of sex as a biological variable in the pathophysiology and treatment of acquired neuromuscular disorders. Our results also establish that longitudinal muscle growth is governed through sex-divergent and non-canonical pathways, which offers valuable directions for future investigations to undertake.

Skeletal muscle exhibits high levels of sex dimorphisms. Female and male muscles typically display heterogeneity in body composition and protein turnover (Griffin and Goldspink, 1973; Smith and Mittendorfer, 2016), regenerative and hypertrophic processes (Kitajima and Ono, 2016; Seko et al., 2020), metabolism and substrate utilization (Rosa-Caldwell and Greene, 2019; Maher et al., 2009), drug pharmacodynamics (Salamone et al., 2022), and even the development of disease-induced pathologies (De Jonghe et al., 2002). While the role of sex in neonatal muscle denervation is largely unknown, we speculate that sex mediates divergent pathways that ultimately lead to contracture formation. Our current discovery of a sex-specific role for MSTN signaling in mediating contractures thus establishes a critical link between neonatal muscle denervation and impaired longitudinal muscle growth. In neonatally denervated female muscles, the improvement in functional muscle length following MSTN inhibition is associated with reduced proteasome subunit activity. Our seemingly paradoxical finding of reduced proteasomal catalytic activity in the absence of any alterations in K48 polyubiquitin is not without precedent. Indeed, the phenomenon of ubiquitin-independent proteasomal degradation has been supported by several prior studies in a subset of proteins, which share common features of belonging to the Intrinsic Disordered Protein (IDP) family (Erales and Coffino, 2014; Jariel-Encontre et al., 2008), such as p21 and p53 (Asher et al., 2005; Chen et al., 2004; Tsvetkov et al., 2010). Since these proteins and their associated intrinsically disordered regions critically regulate various cellular processes, including transcriptional regulation, post-translational modification, and molecular signaling (Wright and Dyson, 2015), it is possible that neonatal muscle denervation induces a direct degradation of these substrates. If so, the sex-specific reduction in proteasome catalytic activity with MSTN inhibition may be due to the preservation of these IDPs, which would not lead to an accumulation of polyubiquitinated substrates. It remains unclear what specific protein substrates are polyubiquitinated with NBPI, and future studies are needed to address this question. Additionally, since we observe discrepancies in the temporal expression of upstream ubiquitin ligases in our current and prior studies (Nikolaou et al., 2019), we wonder if there is a transient temporal window for the upregulation of muscle-specific ligases after NBPI. Thus, a more extensive investigation of ubiquitin ligase expression and function in future studies will provide a better understanding of their role in neonatal denervation and muscle growth.

Our finding of sex-specific reductions in proteasome activity with MSTN inhibition compelled us to thoroughly scrutinize our prior results on proteasome inhibition with bortezomib through the lens of sex differences (Goh et al., 2021). Here, we discovered that bortezomib treatment preferentially prevented elbow and shoulder contractures in male mice after the neonatal stage of muscle development and into skeletal maturity. Though the sex-associated phenotypic differences are more subtle in this context, they further illustrate sex-divergent pathways in the modulation of NBPI-induced contractures. Intriguingly, the effects of MSTN inhibition on proteasome activity in female mice parallel our earlier results of proteasome inhibition with bortezomib (Goh et al., 2021). Specifically, we observed that a 30–45% attenuation in either β1 or β5 subunit activity at 4 weeks post-NBPI is associated with a 50–55% and a 45–60% reduction in elbow and shoulder contractures, respectively. Furthermore, this magnitude of reduction in either subunit activity is associated with an improvement of normalized sarcomere length by 14–15%. The absence of subunit specificity in preventing contractures thus points to a less prominent role for the different proteolytic sites of the 20S proteasome in modulating sarcomerogenesis and muscle length than previously proposed (Goh et al., 2021). Alternatively, the independent alterations in caspase- and chymotrypsin-like activities could potentially reflect different affinities of the two enzymatic processes for specific sarcomeric, cytoskeletal, or regulatory proteins involved in muscle growth and sarcomere addition. This notion is supported by Kisselev et al., 2006, who showed that the unique amino acid sequence of a protein substrate differentially determines the activities of the 20S proteolytic sites during protein degradation, thereby leading to variability in efficacy of the different inhibitors (Kisselev et al., 2006). Importantly, mutations and crystallography of yeast 20S proteasome by Groll et al., 1999 revealed that the three active β subunits are processed autocatalytically and independently of each other, with the processing of one subunit unaffected by the inactivation of another subunit (Groll et al., 1999). The independent processing function of the individual subunits, along with their unique affinities for specific protein substrates, may explain the independent changes in enzymatic activity observed in our current and prior studies. Although, we acknowledge that our findings contradict observations of allosteric interactions among the active subunits observed by Kisselev et al., 1999, specifically between the caspase- and chymotrypsin-like sites and vice versa, in eukaryotic proteasomes (Kisselev et al., 1999). Additional studies are required to reconcile the independent changes of the subunits with total changes in proteasome content, as we continue to develop insights on proteasome function in contracture pathophysiology.

Nevertheless, our collective findings indicate that different upstream pathways in contracture formation between sexes ultimately converge at the 20S proteasome, thereby validating the role of the proteasome as a key mediator of NBPI-induced contractures. Despite these insights, the precise mechanism(s) by which MSTN signaling modulates proteasome activity in denervated female muscles remains to be determined. While we observed altered Akt/mTOR and Smad2/3 signaling with NBPI, which are corroborated by studies from other groups using different mouse denervation models (Castets et al., 2019; MacDonald et al., 2014; Tando et al., 2016). MSTN inhibition failed to impact these canonical pathways in female mice. These findings thus suggest a non-canonical signaling mechanism through which MSTN regulates the 20S proteasome in female muscles after NBPI. Future studies are needed to elucidate this prospective alternative pathway. Further investigations into the molecular interactions between MSTN and proteasome activity are also critical for unraveling the complex regulatory machinery in longitudinal muscle growth, a poorly understood aspect of skeletal muscle biology (Jorgenson and Hornberger, 2019; Jorgenson et al., 2020).

Additionally, it is unclear how proteostasis dysregulation and contractures are mediated in denervated male muscles. To this end, it is conceivable to postulate a role for sex hormones in contracture pathophysiology post-NBPI. An earlier study on juvenile frogs reported a loss of laryngeal muscle fibers only in male frogs following surgical denervation, whereas androgen treatment resulted in an additive effect on total fiber numbers only in innervated female laryngeal muscles (Tobias et al., 1993). These results not only indicate a sexually dimorphic interaction between innervation and the endocrine system in regulating muscle fiber number, but they also suggest a protective effect of the female sex hormones against muscle fiber loss with denervation. Additional studies further verified the role of the female endocrine system in regulating muscle homeostasis. Ovariectomy in adult female rats prevented muscle mass recovery after hindlimb unloading (Sitnick et al., 2006), whereas genetic deletion of estrogen receptor β in muscle stem cells compromised muscle regeneration in juvenile female mice after local injections of barium chloride (Seko et al., 2020). Thus, to gain mechanistic insights on sexual dimorphisms in MSTN-mediated contracture formation, future studies must rigorously interrogate the contributions of sex in contracture pathophysiology by exploring the relationships between the different sex hormones and MSTN signaling in NBPI.

The finding of sex dimorphism in MSTN signaling itself is not without precedent. Targeted deletion of MSTN during skeletal maturity increased masseter mass and bite force in male mice only (Williams et al., 2015). In contrast, long-term MSTN deletion in skeletal muscles increased lean muscle mass only in aged female mice (Tavoian et al., 2019). These discrepancies might be attributable to sex-related differences of MSTN expression itself in skeletal muscles. Indeed, while men are prone to having higher expression of genes encoding ribosomal and mitochondrial proteins, women tend to have higher gene expression of the ACVR2B receptor (Welle et al., 2008). Moreover, there is an increased transcriptional and translational expression of latent MSTN in hindlimb muscles of adult female mice compared to their male counterparts (McMahon et al., 2003). As the increased expression of MSTN and its receptor ligands facilitate higher MSTN activity in females, they conceivably predispose female muscles to more prominent responses from MSTN inhibition. These sex differences in MSTN and receptor gene expression may further account for the mixed outcomes of MSTN inhibition therapy for treating Duchenne Muscular Disease and other muscle disorders (Lee, 2021; Rybalka et al., 2020). In our current study, these intrinsic sex differences could potentially elucidate how pharmacologic MSTN inhibition confers greater neonatal growth in normally innervated female muscles and protects against atrophy in denervated muscles of female mice. Future studies should perform a comprehensive analysis of the expression of MSTN and other TGF-β superfamily members (such as Activin A), as well as the associated receptors (ACVR2 and ACVR2B) across sexes and muscle groups to account for potential sex and/or muscle-dependent regulation.

Our discovery of sex dimorphisms in denervated muscle growth also calls into question prior conclusions on the role of MSTN signaling in denervation-induced atrophy from studies analyzing only male mice. In a juvenile mouse model of sciatic nerve resection, MacDonald et al. reported an inability of both rapamycin and ACVR2B-Fc treatment to prevent atrophy in male mice, and surmised that denervation atrophy is not Akt/mTOR or MSTN-dependent (MacDonald et al., 2014). These findings are similar to our current observations of male mice in a neonatal mouse model of NBPI. In our present study, we extend MacDonald et al.’s earlier findings by revealing a novel role for MSTN in mediating atrophy solely in denervated muscles of female mice. Importantly, the sex dimorphisms in muscle atrophy are not limited to neonatal denervation, as sex differences in the pathophysiology of muscle atrophy have also been documented by an independent group using an adult mouse model of tenotomy (Meyer et al., 2022). Overall, these latest discoveries underscore the need to account for sex as a biological variable in future studies investigating in different models of muscle atrophy. Our study is not without limitations. First, the small size of our denervated muscles precluded the use of the same muscles for all analyses, instead requiring smaller subgroup sizes as well as different muscles for certain biochemical endpoints (Akt, Smad2/3, MuRF1, and Atrogin-1). We therefore acknowledge that our study may be underpowered to detect smaller effects in certain parameters of protein dynamics, specifically signaling proteins and ubiquitin ligases. We also acknowledge that the precision of our findings would be further enhanced with the use of the same muscle type across all of our morphological, physiological, and biochemical analyses. Furthermore, we cannot be certain whether the lack of normal distribution observed for elbow extension in female NBPI mice with MSTN inhibition (Figure 3B) is attributed to different treatment response or an inherent variability in contracture formation with NBPI. Indeed, we have consistently observed varying levels of elbow joint mobility and contracture severity in our mouse model of NBPI in prior studies (Nikolaou et al., 2011; Weekley et al., 2012; Nikolaou et al., 2014; Nikolaou et al., 2015; Nikolaou et al., 2019; Goh et al., 2021; Ho et al., 2021). At this point, we do not have sufficient mechanistic insights to explain this variability. However, despite this variability, the effects of MSTN inhibition seen in this study remain apparent and statistically significant.

Additionally, we observed that the ACVR2B-Fc decoy receptor attenuated growth of other non-skeletal muscle tissues, including the heart, spleen, and bone. Indeed, the broad ligand specificity of ACVR2B-Fc increases the risk of toxicity to non-skeletal muscle tissues and cell types, ranging from epistaxis to telangiectasias (Campbell et al., 2017). Our observations are thus consistent with prior reports of off-target effects with pharmacologic MSTN inhibition (Lee, 2021). The off-target effects we observed in our study could have been minimized through genetic models to manipulate MSTN signaling in the skeletal muscle. While such genetic models could reveal additional insights in contracture formation, the off-target effects do not fully explain the sex specificity of our outcomes. As MSTN plays a key role in cardiac energy homeostasis (Biesemann et al., 2014), the attenuation in heart size of male mice could impair cardiac function and lead to reduced muscle activity. We are unable to ascertain this possibility as we did not perform any metabolic measurements in the current study, though there were no observable differences in spontaneous cage activity with ACVR2B-Fc treatment. Of particular note is the effect of MSTN inhibition on bone growth, which could potentially confound our measurements of muscle parameters that are normalized to humerus length. However, the reduced bone length in male denervated limbs following MSTN inhibition would, if anything, cause an apparent increase in muscle size and/or rescue of contractures. The absence of such muscle findings despite this effect on bone growth reinforces the lack of a beneficial effect of MSTN inhibition on male denervated muscle, further underscoring the sex dimorphisms seen in muscle parameters. Prior studies have failed to find an effect of embryonic MSTN deletion on humerus length in adult mice, although the use of a mixed-sex approach may have masked sex-related differences (Hamrick et al., 2002). Finally, although we identified a link between MSTN inhibition and proteasome activity without perturbations in canonical MSTN signaling pathways, the molecular mechanisms by which MSTN could be regulating proteasome activity remain unclear. Potential non-canonical pathways could include p21-activated kinase 1 (PAK1) signaling, which has recently been shown to be permissive to gains in skeletal muscle mass with MSTN inhibition (Barbé et al., 2021). Additional non-canonical alternatives that should be further explored include TAK1 and its downstream targets JNK and p38, as well as Ras and its downstream targets MEK1 and ERK1/2. Alternatively, MSTN might target contractures through the autophagy–lysosome pathway instead (Wang et al., 2015). Future studies are needed to fully elucidate mechanistic links and complete our understanding of the role of MSTN signaling in contracture pathophysiology.

For a more nuanced interpretation of our findings, we must also consider the specificity of the ACVR2B-Fc decoy receptor as a ligand trap, as mentioned above. Previous studies have reported that many members of the TGF-β superfamily, such as Activin A and GDF-11, are capable of binding Activin Type IIB receptors (Oh et al., 2002; Olsen et al., 2015). This broad array of targeted ligands makes the decoy receptor a potent inhibitor of not only MSTN, but also an inhibitor of various TGF-β family members that signal downstream of Activin binding. Of note, simultaneous inhibition of MSTN and Activin A leads to more effective muscle hypertrophy and force production in rodents and primates (Latres et al., 2017). Consequently, this ligand trap putatively elicits more robust increases in skeletal muscle growth than MSTN-specific agents (Lee, 2021). In our current study, it is possible that Activin A is differentially expressed and/or blocked with ACVR2B-Fc between sexes, which may account for the sex dimorphisms observed in neonatally denervated muscles. Indeed, a sex-specific role for Activin A in pancreatic ductal adenocarcinoma (PDAC)-induced cachexia has recently been reported by Zhong et al., 2022. The authors observed that sex-specific differences in endogenous levels of Activin A contribute to sex dimorphisms in PDAC-induced cachexia, as well as the differential outcomes of ACVR2B-Fc treatment in attenuating tumor-induced Activin (Zhong et al., 2022). Alternatively, we also wonder whether Activin A and/or other TGF-β family members are more acutely involved in the regulation of neonatal longitudinal muscle growth and contractures than MSTN. Future work with genetic models and expression profiles for the various TGF-β superfamily members are necessary to address these limitations in our current study. Finally, we cannot yet comment on the long-term efficacy of MSTN inhibition beyond 4 weeks following denervation. However, the focus of this study was to establish a proof of concept that targeting a muscle-specific regulator of proteostasis (MSTN) can effectively prevent contractures during the critical window of neonatal muscle growth (4 weeks). Furthermore, we have previously established that contracture formation plateaus at 4 weeks post-NBPI, making this time point ideal for such a proof of concept investigation. As we have established that MSTN inhibition does prevent contractures in a sex-specific manner during this stage of development, ongoing studies are currently planned to build on our findings here by interrogating the efficacy of this pharmacological approach in preventing contractures at skeletal maturity and beyond.

### Conclusion

In conclusion, we established several critical insights in this current study that enhance our understanding of contracture pathophysiology and establish the framework for future investigations. First, the efficacy of MSTN inhibition at rescuing contractures, at least in female mice, reinforces the role of skeletal muscle biology in contracture formation and provides proof of concept for potential muscle-specific therapies for contracture prevention and treatment. Second, the sex-specific improvement in longitudinal muscle growth is associated with a corresponding reduction in proteasome activity, offering further evidence that the proteasome is a key regulator of muscle contractures. Third, the rescue in longitudinal growth without perturbations in the canonical signaling pathways with which MSTN regulates proteostasis suggests a potential non-canonical pathway governing muscle length. Lastly, the discovery of sex dimorphisms in our current study highlights the importance of sex in the pathophysiology of denervation atrophy, muscle growth, and pediatric muscle contractures. Moving forward, we strongly advocate the inclusion of sex as a biological variable in both mechanistic and translational studies on muscle atrophy and acquired neuromuscular diseases.

## Materials and methods

**Key resources table:** 

Reagent type (species) or resource	Designation	Source or reference	Identifiers	Additional information
Biological sample (CD-1 IGS mouse)	Mouse	Charles River		Strain code 022
Peptide, recombinant protein	ACVR2B-Fc	https://doi.org/10.1073/pnas.0505996102; https://doi.org/10.1073/pnas.1206410109		Dr. Se-Jin Lee (The Jackson Laboratory)
Chemical compound, drug	Bortezomib	https://doi.org/10.1096%2Ffj.202002194		Sigma-Aldrich #5043140001

### NBPI surgical mouse model

To assess the role of MSTN signaling in pediatric muscle contracture formation, we utilized our surgical mouse model of postganglionic NBPI where injury to the brachial plexus at postnatal day (P)5 induces contractures within 4 weeks after denervation (Nikolaou et al., 2019; Goh et al., 2021; Ho et al., 2021). This type of injury completely denervates all muscles in the forelimb, including flexors (brachialis, biceps), and extensors (triceps). Briefly, unilateral, global (C5–T1), post-ganglionic NBPIs were surgically created in P5 wildtype female and male mice (Charles River; CD-1 IGS mouse, strain code 022) by extraforaminal nerve root excision under isoflurane anesthesia. Following the surgery, mice were returned to their mums and housed in standard cages with bio-huts on a 12-hr light/12-hr dark cycle, with nutrition and activity ad libitum. In order to ensure permanent deficits and to prevent potential confounding consequences of reinnervation, we validated deficits in motor function both postoperatively and prior to sacrifice. We excluded from the study mice that displayed preserved or recovered movement in the denervated limb. Based on these criteria, one female mouse was excluded from the study.

### MSTN inhibition

Beginning 1 day prior to surgery and continuing for 4 weeks after surgery, we pharmacologically inhibited the MSTN pathway by treating wildtype neonatal mice with a soluble decoy receptor fused to an Fc domain (ACVR2B-Fc; The Jackson Laboratory, Farmington, CT) (Lee et al., 2005; Lee et al., 2012). The binding of MSTN to the Fc domain inhibits MSTN from binding to ACVR2B, which blocks MSTN activity in the muscle, ultimately leading to increased muscle protein synthesis and robust muscle growth (Lee et al., 2005). The dosage frequency for the ACVR2B decoy receptor varies from 3 (Lee et al., 2012; Goh et al., 2019), 5–6 (Lee et al., 2020), and 7 days (Lee et al., 2005; Lee et al., 2012). Since this is the first known study in which this drug was tested in neonatal mice, we utilized the lowest dosage frequency in order to prevent potential toxicity (Lee et al., 2005; Lee et al., 2012). Hence, the soluble decoy receptor was administered weekly at a dose of 10 mg/kg via intraperitoneal injections, whereas Dulbecco’s phosphate-buffered saline (PBS) was used as a control in a separate litter of mice (Figure 1A). This regimen was performed twice to obtain two separate control groups and two corresponding drug-treated groups. All control and experimental groups in this study were randomized by litter, with all treatments administered at noon and in the respective cages. Milk spots in non-weaned mice and body weight at all ages were monitored daily for adequate nursing and signs of toxicity. Mice displaying an inability to nurse/self-feed post-weaning were euthanized immediately.

### Assessment of contractures

Mice were euthanized at 4 weeks post-NBPI (P33) by CO_2_ asphyxiation, whereupon passive range of motion of the elbow and shoulder joints were assessed to determine contracture severity. Prior to sacrifice, mice were fasted for 4 hr and then administered 21.8 mg/kg puromycin (Sigma-Aldrich #P7255) intraperitoneally for 30 min, which gets incorporated into newly formed peptide chains (Schmidt et al., 2009). Immediately post-sacrifice, digital photography images of bilateral elbows and shoulders were captured at maximum passive extension and external rotation, respectively. Elbow flexion and shoulder internal rotation contractures were subsequently calculated in AxioVision (Zeiss) (Nikolaou et al., 2019; Goh et al., 2021; Ho et al., 2021). This previously validated method of assessing passive range of motion and contracture severity was performed with blinding to treatment groups. The forelimb images shown in Figure 3A, D are representative of their respective samples, and have been processed to reflect comparable levels of sharpness, brightness, and contrast for illustrative purposes. However, no image manipulation was performed prior to measurements.

### Tissue collection and preparation

Following digital photography, we harvested bilateral biceps muscles (long head only), bilateral triceps muscles, hearts, and spleens. Biceps, hearts, and spleens were also weighed. Both biceps and triceps were then flash frozen and stored at −80°C for subsequent analysis of protein dynamics and signaling pathways. The remaining forelimbs (with intact brachialis muscles) were positioned at 90° elbow flexion on cork, and imaged with digital radiographs for humerus length. Bilateral forelimbs were subsequently fixed in 10% formalin at 90° elbow flexion to avoid sarcomere relaxation with muscle removal. Following 48 hr of fixation, bilateral brachialis muscles were dissected, soaked in 25% Lugol solution (Sigma-Aldrich #32922) overnight, and imaged by micro computed tomography (MicroCT) for assessment of whole-muscle cross-sectional area and volume (Nikolaou et al., 2019; Goh et al., 2021; Ho et al., 2021). Brachialis muscles were then recovered by soaking in PBS overnight at 4°C, digested in 15% sulfuric acid for 30 min to obtain muscle bundles, and imaged for sarcomere length.

### Ex vivo high-resolution studies

MicroCT was performed using a Siemens Inveon PET/SPECT/CT Scanner (Siemens Medical Solutions, Malvern, PA, USA) as previously described (Ho et al., 2021). The cone-beam CT parameters were as follows: 360° rotation, 1080 projections, 1300-ms exposure time, 1500-ms settle time, 80-kVp voltage, 500-µA current, and effective pixel size 17.67 µm. Briefly, acquisitions were reconstructed using a Feldkamp algorithm with mouse beam-hardening correction, slight noise reduction, and 3D matrix size 1024 × 1024 × 1536, using manufacturer-provided software. Protocol-specific Hounsfield unit (HU) calibration factor was applied.

### Muscle length

Although the functional length of a whole muscle is defined by its total number of sarcomeres in series, morphological constraints restrict its direct measurement (Nikolaou et al., 2019). To overcome these limitations, we measured the average sarcomere length at 90° elbow flexion to determine the relative functional length of the brachialis muscles on control and denervated limbs. As previously described, elongated (overstretched) sarcomeres indicate fewer sarcomeres in series (Goh et al., 2021). This correlates with a shorter functional whole-muscle length, because the fewer sarcomeres a muscle has in series, the more each sarcomere has to stretch to accommodate any given position.

Following overnight recovery in PBS at 4°C, the brachialis muscles were digested in 15% sulfuric acid for 30 min, and then dissected into muscle bundles for imaging with differential interference contrast (DIC) microscopy at ×40 on a Nikon Ti-E SpectraX widefield microscope. Six images representing different muscle bundles of the same brachialis were acquired per muscle. Average sarcomere length of the brachialis was subsequently determined by measuring a series of 10 sarcomeres from each of the six representative DIC images with the AxioVision program as previously described (Nikolaou et al., 2019; Goh et al., 2021; Ho et al., 2021), with blinding to treatment groups. Representative sarcomere images in Figure 4A have been cropped to identical sizes, and processed to reflect comparable levels of sharpness, brightness, and contrast for illustrative purposes. No image manipulation was performed prior to measurements.

### Humerus length and whole-muscle size

To quantify humerus lengths from digital radiographs, AxioVision software was used to measure the distance between the proximal humerus physis to the distal articular surface (Nikolaou et al., 2019; Goh et al., 2021; Ho et al., 2021). Whole-muscle cross-sectional area and muscle volume of the brachialis were obtained by processing the MicroCT scans into digital imaging and communications in medicine (DICOM) images with Fiji programs (Segmentation Editor and 3D Viewer, respectively), and normalized to humerus length of the corresponding forelimb (Nikolaou et al., 2019; Goh et al., 2021; Ho et al., 2021). All measurements were performed with blinding to treatment groups. For illustrative purposes, raw DICOM files were processed in IMARIS software (Bitplane, Zurich, Switzerland) to create whole-muscle images presented in Figures 1B and 2A.

### Assessment of protein dynamics

To determine the effects of MSTN inhibition on muscle proteostasis, we assayed for protein synthesis by detection of the antibiotic puromycin using non-radioactive surface sensing of translation (SUnSET) (Goodman et al., 2011; Schmidt et al., 2009), and protein degradation by quantification of the amount of proteins marked for ubiquitination, with blinding to treatment groups (Nikolaou et al., 2019). Bilateral biceps or triceps muscles were first bead homogenized (TissueLyser II; Qiagen) at a frequency of 30 Hz for 3 cycles of 2 min in lysis buffer [10 mM Tris (pH 7.4), 1 mM ethylenediaminetetraacetic acid (EDTA), 1 mM dithiothreitol, 0.5% Triton X-100, 2.1 mg/ml NaF] containing protease and phosphatase inhibitor cocktails (5 μl/ml; Sigma-Aldrich #P8340 and #P5726, respectively) (Goh and Millay, 2017). Following centrifugation at 15,000 × *g* for 10 min at 4°C, the amount of protein in supernatants was quantified using Bradford protein assay. Muscle homogenates were then heated at 65°C for 30 min, separated on 4–20% sodium dodecyl sulfate–polyacrylamide gel electrophoresis (SDS–PAGE) gels (10–30 µg of proteins), and transferred at 4°C to Immobilon-fluorescently labeled (Immobilon-FL) polyvinylidene fluoride (PVDF) membranes. Homogenates from adult mouse plantaris muscles that had been subjected to 7 days of mechanical overload were included in each gel as positive controls (Goh and Millay, 2017). Membranes were subsequently blocked in 5% bovine serum albumin (BSA) in TRIS-buffered saline (TBS)-Tween (pH 7.5), and incubated overnight at 4°C with an antibody against puromycin (1:1000; MilliporeSigma #MABE343), K48-linkage-specific-polyubiquitin (1:1000, Cell Signaling #8081), MuRF1 (1:1000; Abcam #ab183094), or Atrogin-1 (1:500; Invitrogen #PA5-19056). GAPDH (1:5000; Cell Signaling #2118) served as a control for sample loading. Membranes were then washed and incubated with IRDye 680CW anti-goat or 800CW anti-mouse IgG2a (1:5000; LI-COR Biosciences) or DyLight anti-rabbit (1:5000; Cell Signaling #5151) secondary antibodies. Following image detection on the Odyssey infrared detection system (LI-COR Biosciences), the relative abundance of puromycin incorporation (30 µg), K48-linked protein levels (30 µg), MuRF1 (10 µg), and Atrogin-1 protein levels (10 µg) were quantified using the Image Studio Lite program (LI-COR Biosciences), and normalized to corresponding GAPDH protein levels (Nikolaou et al., 2019; Goh and Millay, 2017).

To determine the effects of MSTN inhibition on proteasome activity, we assayed for 20S proteasome subunit catalytic activity with blinding to treatment groups as previously described (Nikolaou et al., 2019; Goh et al., 2021). Briefly, bilateral triceps muscles were homogenized in 20 mM of Tris–HCl (pH 7.2), 0.1 mM of EDTA, 1 mM of 2-mercaptoethanol, 5 mM of adenosine triphosphate (ATP), 20% of glycerol, and 10% (vol/vol) IGEPAL CA-630 (Sigma-Aldrich #I8896) (Penna et al., 2016). Following centrifugation described above, protein concentration was determined using the Pierce 660 nm protein assay kit (Thermo Scientific #22662). The caspase-like activity of the 20S proteasome β1 catalytic subunit and chymotrypsin-like activity of the β5 catalytic subunit were assayed with 10 μg total protein per muscle, through detection of the 7-amino-4-methylcoumarin (AMC) labeled fluorogenic peptide substrates Z-LLE-AMC (Boston Biochem #S-230) and LLVY-AMC (Chemicon #APT280), respectively. Both activity assay kits specifically target the 20S proteasome, with cleavage of the fluorogenic substrates occurring via the 20S subunits. The duration of substrate incubation is 2 hr at 37°C, following which assays were carried out in a white opaque polystyrene 96-well plate, with endpoint fluorescence measured at 380/460 nm on a SpectraMax M5 microplate reader (Molecular Devices). Relative fluorescence units were calculated per μg protein.

Regarding the use of different muscles for analyses of protein dynamics compared to physiological endpoints, the dissection of fiber bundles for sarcomere length assessment described above prevents any subsequent processing of the brachialis. Furthermore, while the biceps long head was processed for puromycin incorporation and total levels of K48 polyubiquitin, the limited amounts of protein available in denervated biceps precluded their use for assessment of ubiquitin ligases and the 20S proteasome subunits. Consequently, triceps muscles were used to assess muscle-specific ubiquitin ligases, proteasome activity, and the various signaling pathways described below.

### Assessment of signaling pathways

To assess the effects of MSTN inhibition on downstream pathways, we assayed for Akt/mTOR and Smad2/3 signaling, with blinding to treatment groups. Briefly, triceps muscle homogenates were heated at 65°C for 30 min, separated on 10% SDS–PAGE gels (20 µg of proteins), and transferred at 4°C to PVDF membranes (Immobilon-FL) (Goh and Millay, 2017). Membranes were subsequently blocked in 5% BSA/TBS-Tween, and incubated overnight at 4°C with an antibody against phosphorylated Akt (Ser473) (1:750; Cell Signaling #9271), total Akt (1:750; Cell Signaling #9272), phosphorylated Smad2 (Ser465/467) (1:1000; Cell Signaling #3108), total Smad2 (1:1000; Cell Signaling #5339), phosphorylated Smad3 (Ser423, Ser425) (1:2000 Abcam #ab51451), or total Smad3 (1:2000; Abcam #ab28379). GAPDH (1:5000; Cell Signaling #2118) served as a control for sample loading. Membranes were then washed and incubated with a DyLight anti-rabbit secondary antibody (1:5000; Cell Signaling #5151). Western blot signals were subsequently imaged and the relative abundance of phosphorylated and total protein levels of Akt, Smad2, and Smad3 were quantified as described above.

### Statistical analysis

All statistical tests were performed with Prism 8 software (GraphPad). For all continuous data sets that contained at least four independent samples, outliers were detected a priori by Grubbs’ test and excluded. To compare differences in values between two data sets, all data were subsequently tested for normality with the Shapiro–Wilk test. Normally distributed data were compared with unpaired two-tailed Student’s *t*-tests (Figure 1E, H), whereas non-normally distributed data were compared using the Mann–Whitney *U*-tests (Figure 3—figure supplement 1A). For data sets with two independent variables (sex and treatment), a two-way analysis of variance (ANOVA) with Bonferroni correction for multiple comparisons was performed. For data sets with three independent variables (sex, treatment, denervation), a three-way ANOVA with repeated measures between forelimbs and Bonferroni correction for multiple comparisons was performed. All data are presented as mean ± standard deviation (SD). The degree of significance between data sets is depicted as follows: *p < 0.05, **p < 0.01, ***p < 0.001, and ****p < 0.0001. A priori power analyses based on prior work determined that 6 mice per group were required to detect a 10° difference in contractures and a 0.2-µm difference in sarcomere lengths at 80% power between experimental conditions. A total of 32 mice (17 female – 10 control, 7 experimental; and 15 male – 8 control, 7 experimental) were used in this study.

### Ethical statement

This study was performed in strict accordance with recommendations in the Guide for the Care and Use of Laboratory Animals of the National Institutes of Health. All rodents were handled according to approved Institutional Animal Care and Use Committee (IACUC) protocols (#2020-0067) of the Cincinnati Children’s Hospital Medical Center, and every effort was made to minimize suffering. This study also adhered to the Animal Research: Reporting of In Vivo Experiments (ARRIVE) 2.0 guidelines, and a checklist is provided with the manuscript.

### Material availability statement

All data generated or analyzed during this study are included in the manuscript and supporting files.

## Data Availability

All data generated or analyzed during this study are included in the manuscript and supporting file.

## Additional files

MDAR checklist
