# Supplementary figures and images for: Sex-specific role of myostatin signaling in neonatal muscle growth, denervation atrophy, and neuromuscular contractures
Source: eLife. 2022 Oct 31;11:e81121. doi: 10.7554/eLife.81121 (PMC9873256; doi:10.7554/eLife.81121)

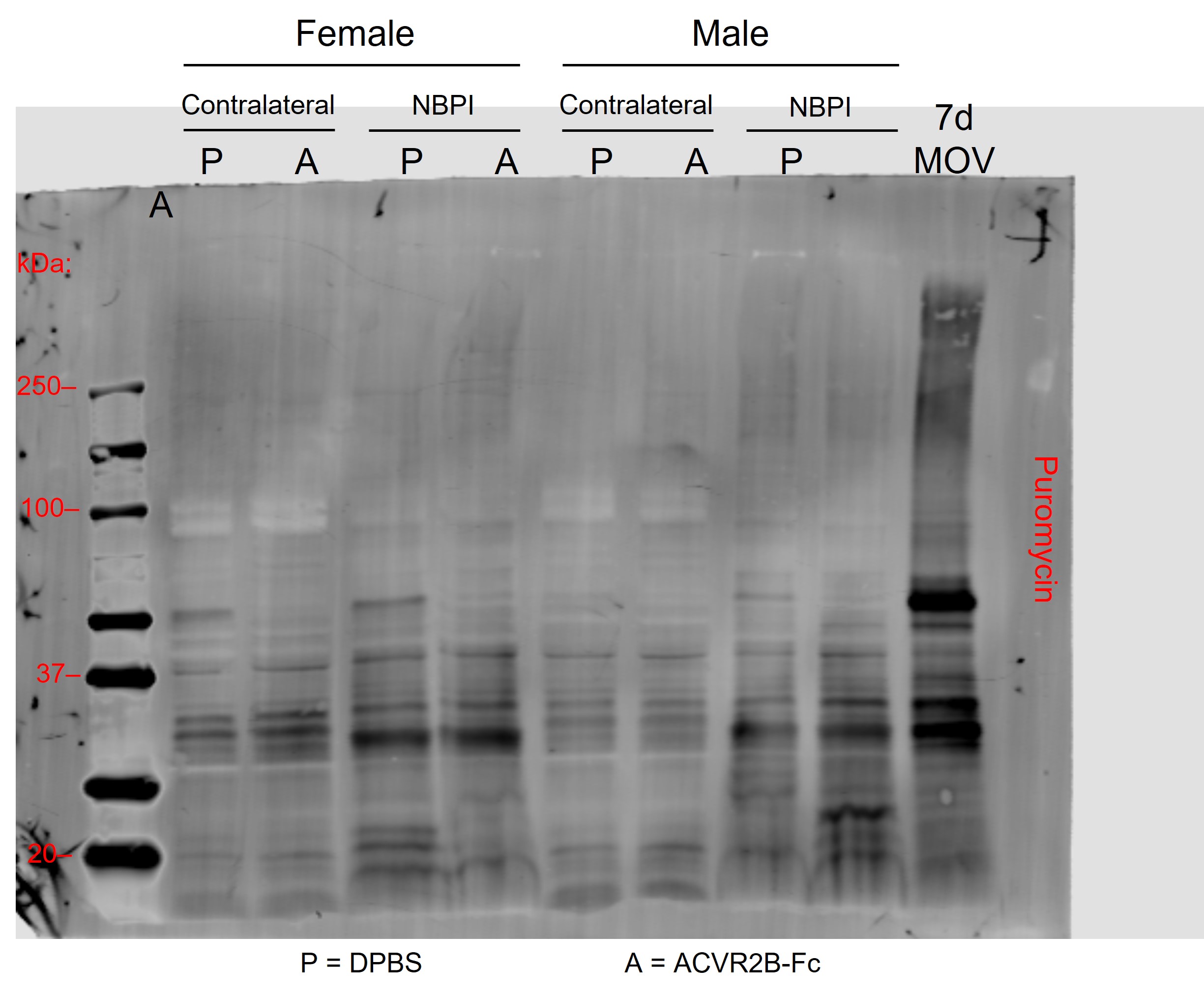

Supplement: Figure 5—source data 1. [file elife-81121-fig5-data1.zip › Figure 5-source data 1/Figure 5a Puromycin original.jpg]

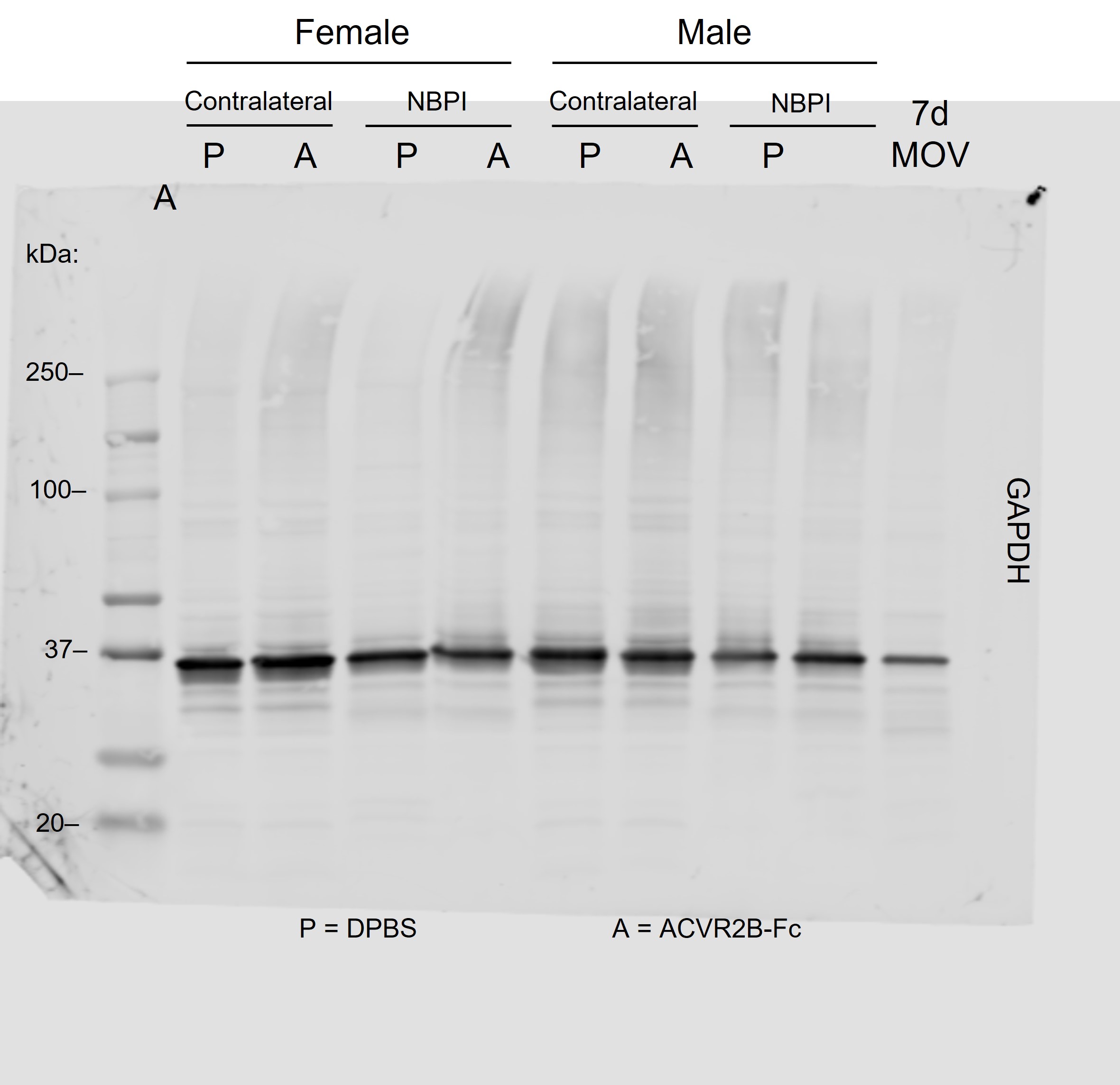

Supplement: Figure 5—source data 2. [file elife-81121-fig5-data2.zip › Figure 5-source data 2/Figure 5a GAPDH original.jpg]

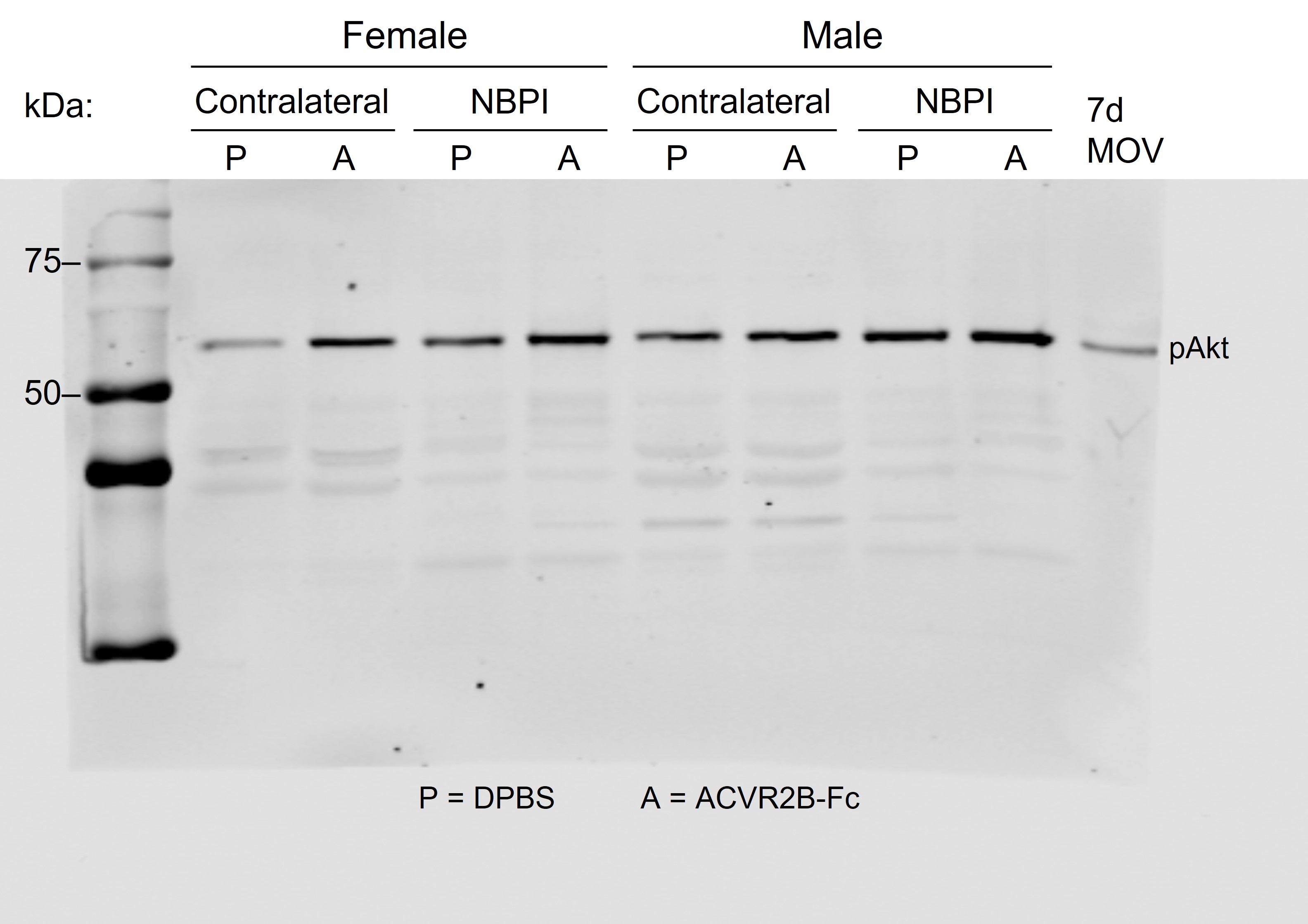

Supplement: Figure 5—source data 3. [file elife-81121-fig5-data3.zip › Figure 5-source data 3/Figure 5c pAkt original.jpg]

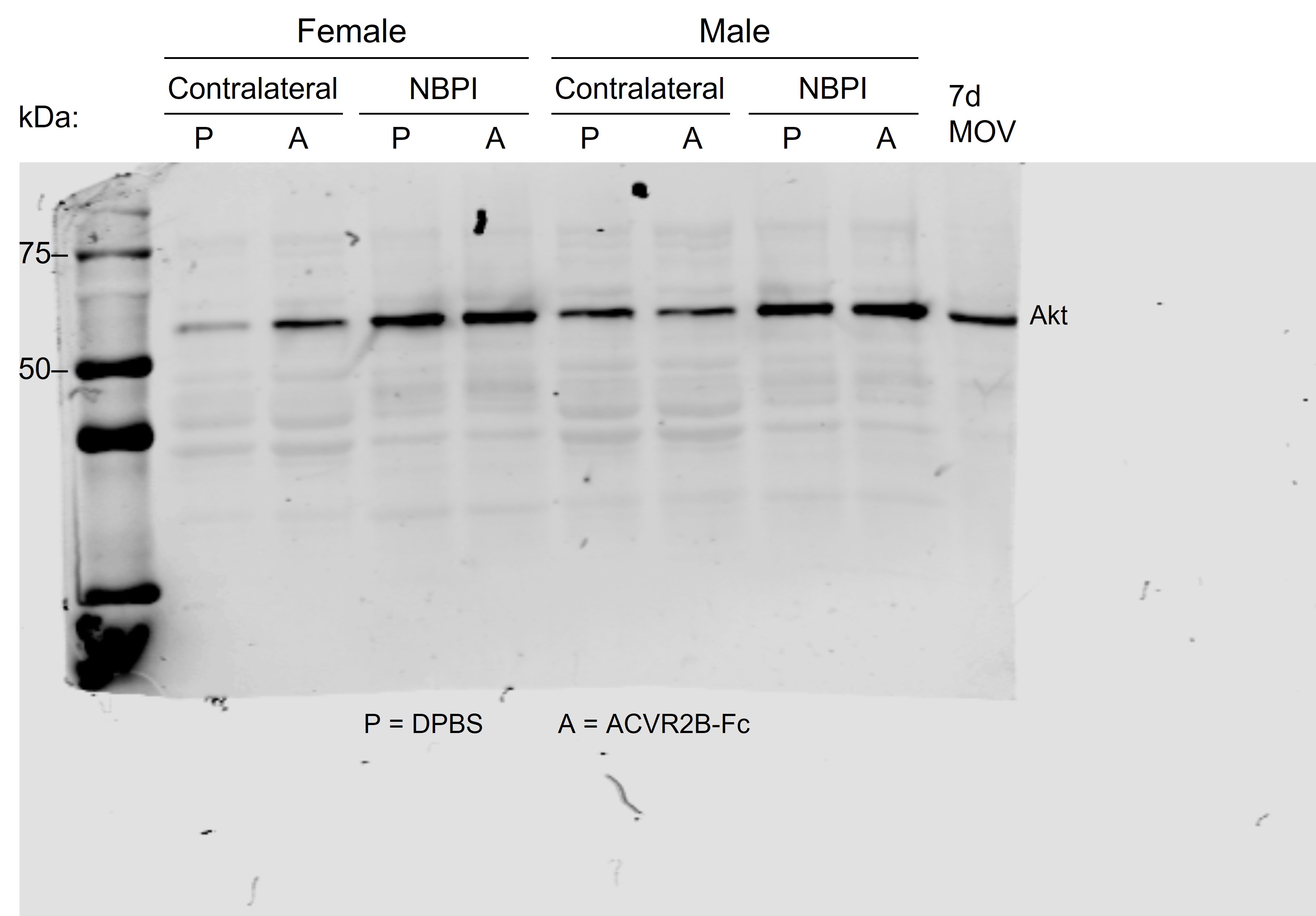

Supplement: Figure 5—source data 4. [file elife-81121-fig5-data4.zip › Figure 5-source data 4/Figure 5c Akt original.jpg]

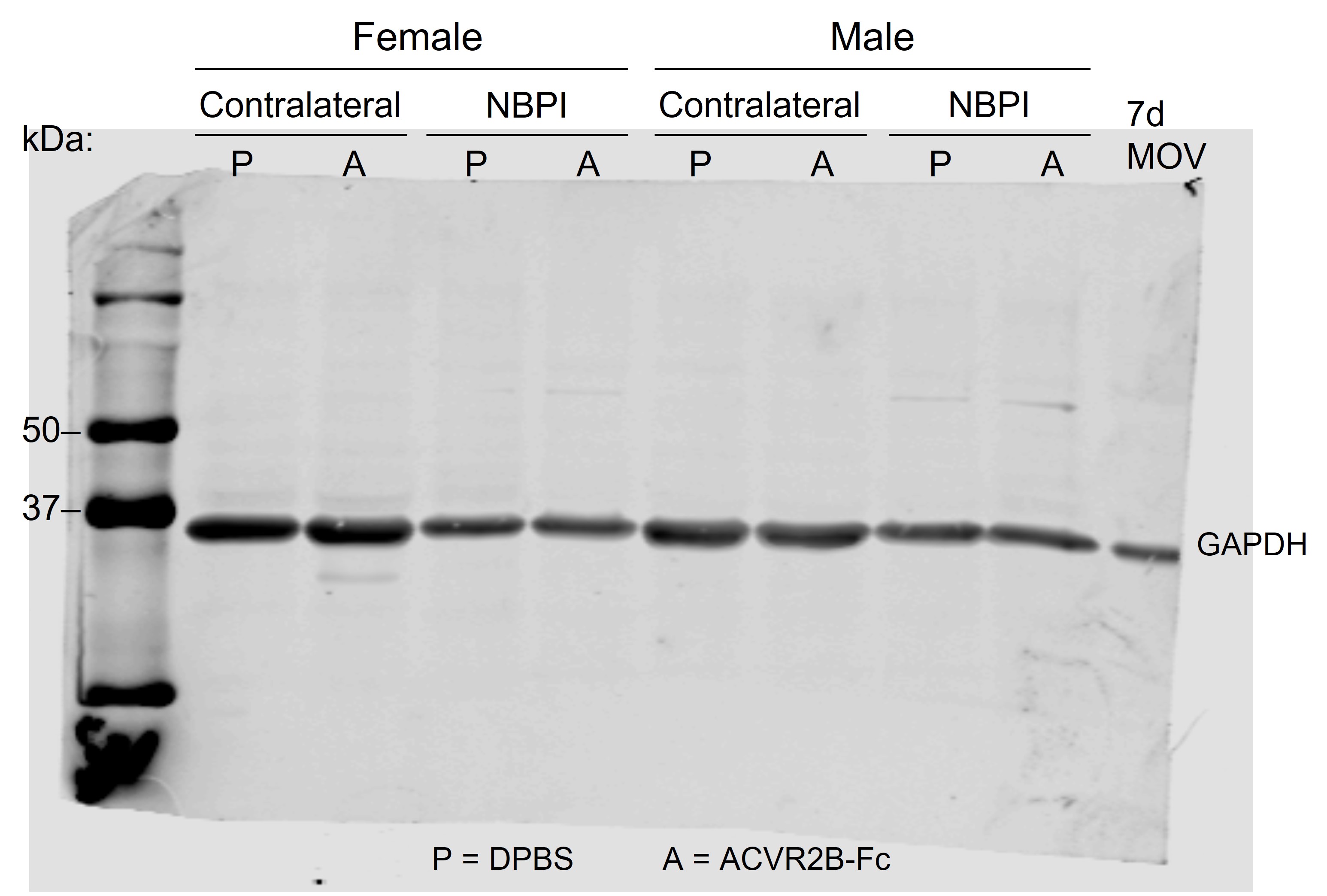

Supplement: Figure 5—source data 5. [file elife-81121-fig5-data5.zip › Figure 5-source data 5/Figure 5c GAPDH original.jpg]

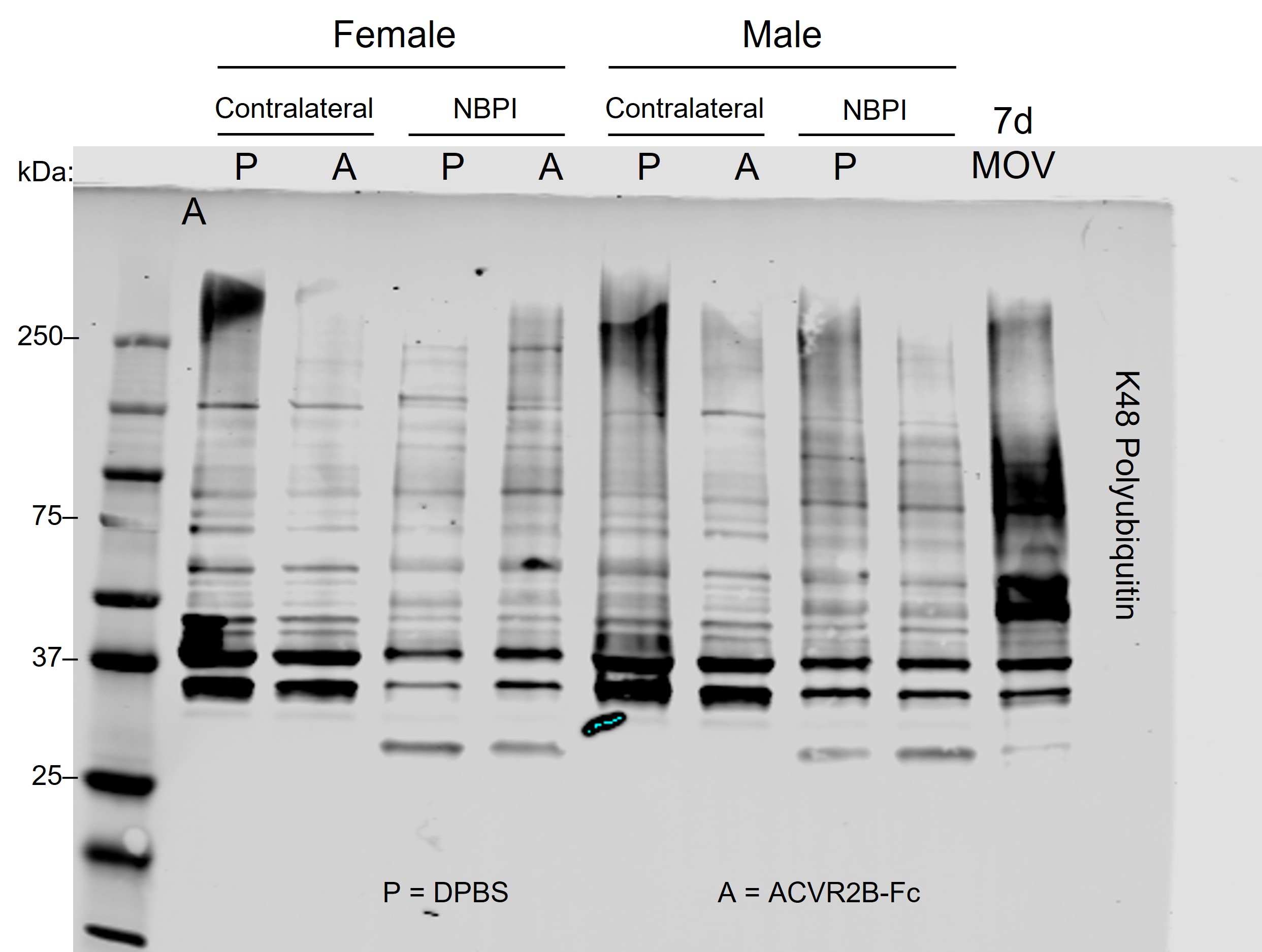

Supplement: Figure 6—source data 1. [file elife-81121-fig6-data1.zip › Figure 6-source data 1/Figure 6a K48 Polyubiquitin original.jpg]

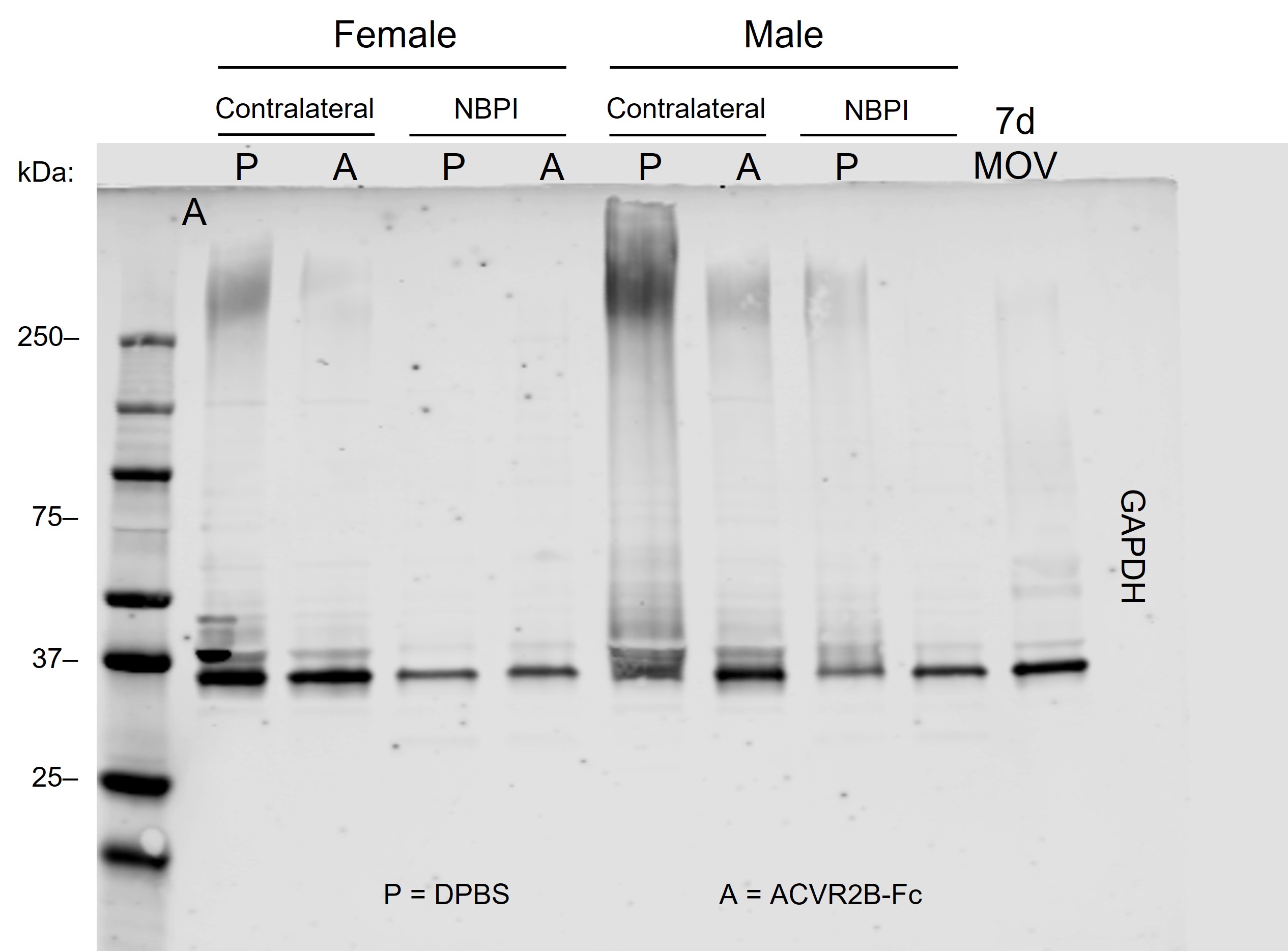

Supplement: Figure 6—source data 2. [file elife-81121-fig6-data2.zip › Figure 6-source data 2/Figure 6a GAPDH original.jpg]

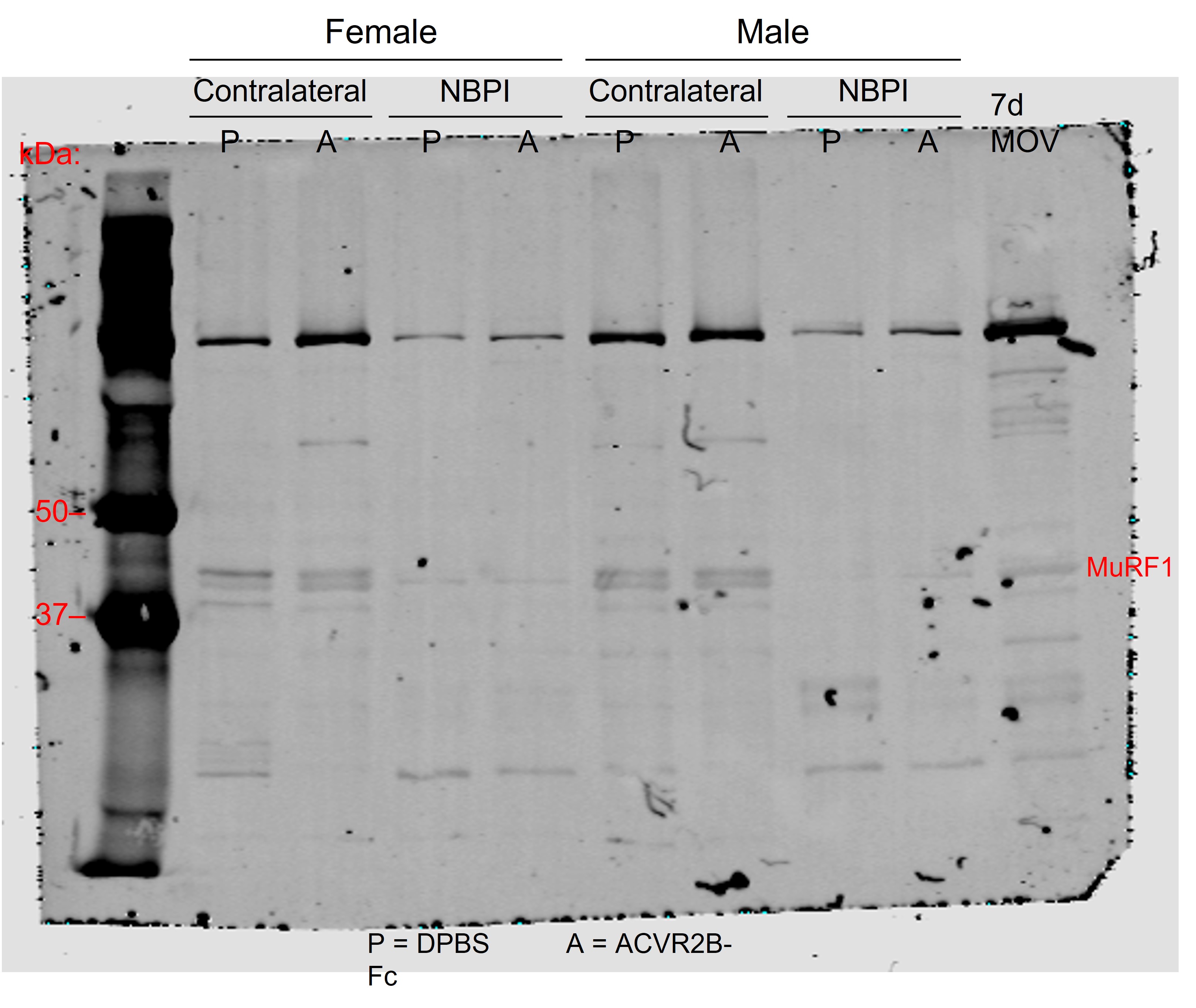

Supplement: Figure 6—figure supplement 1—source data 1. [file elife-81121-fig6-figsupp1-data1.zip › Figure 6-figure supplement 1-source data 1/Figure 6-figure supplement 1a MuRF1 original.jpg]

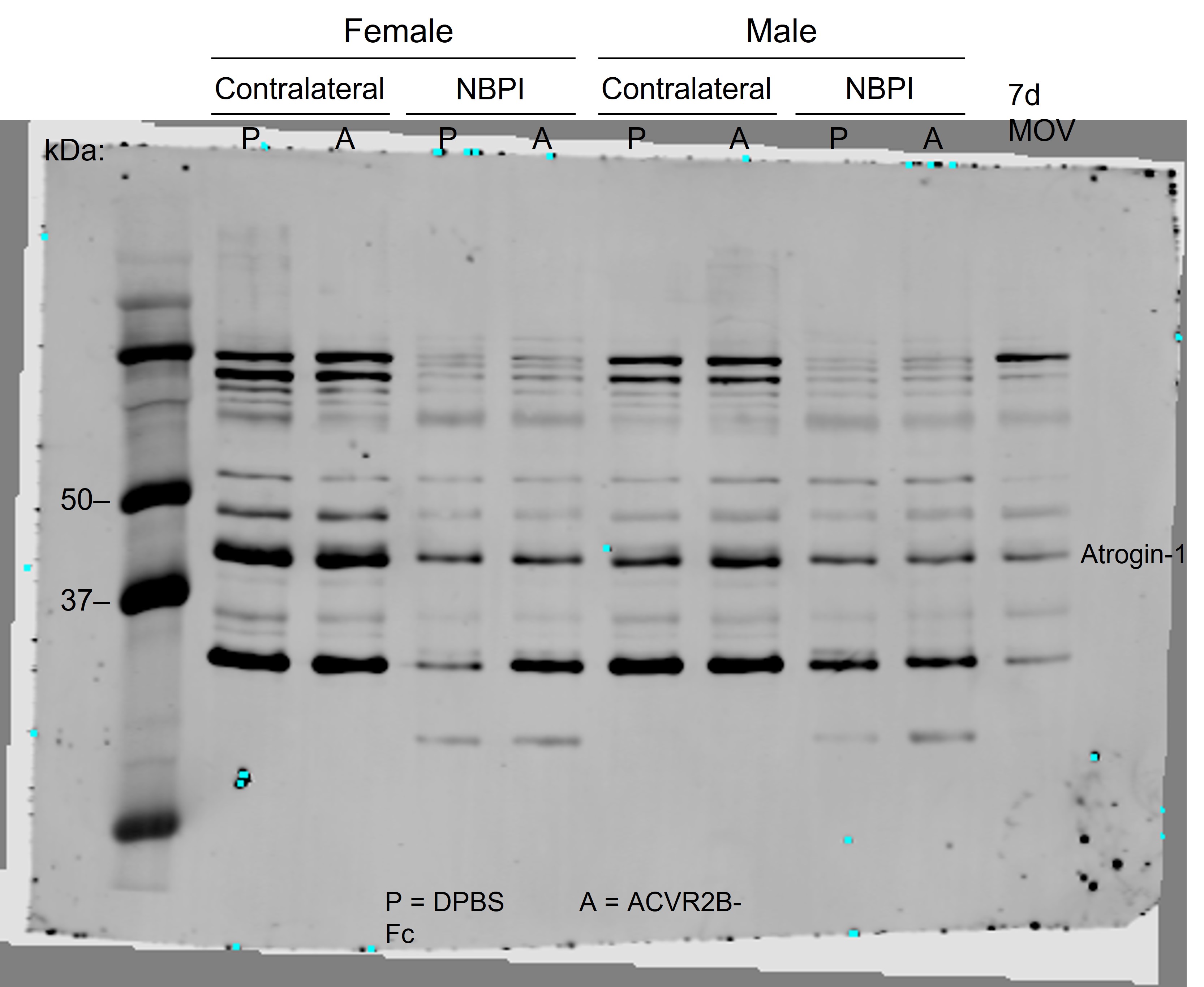

Supplement: Figure 6—figure supplement 1—source data 2. [file elife-81121-fig6-figsupp1-data2.zip › Figure 6-figure supplement 1-source data 2/Figure 6-figure supplement 1a Atrogin-1 original.jpg]

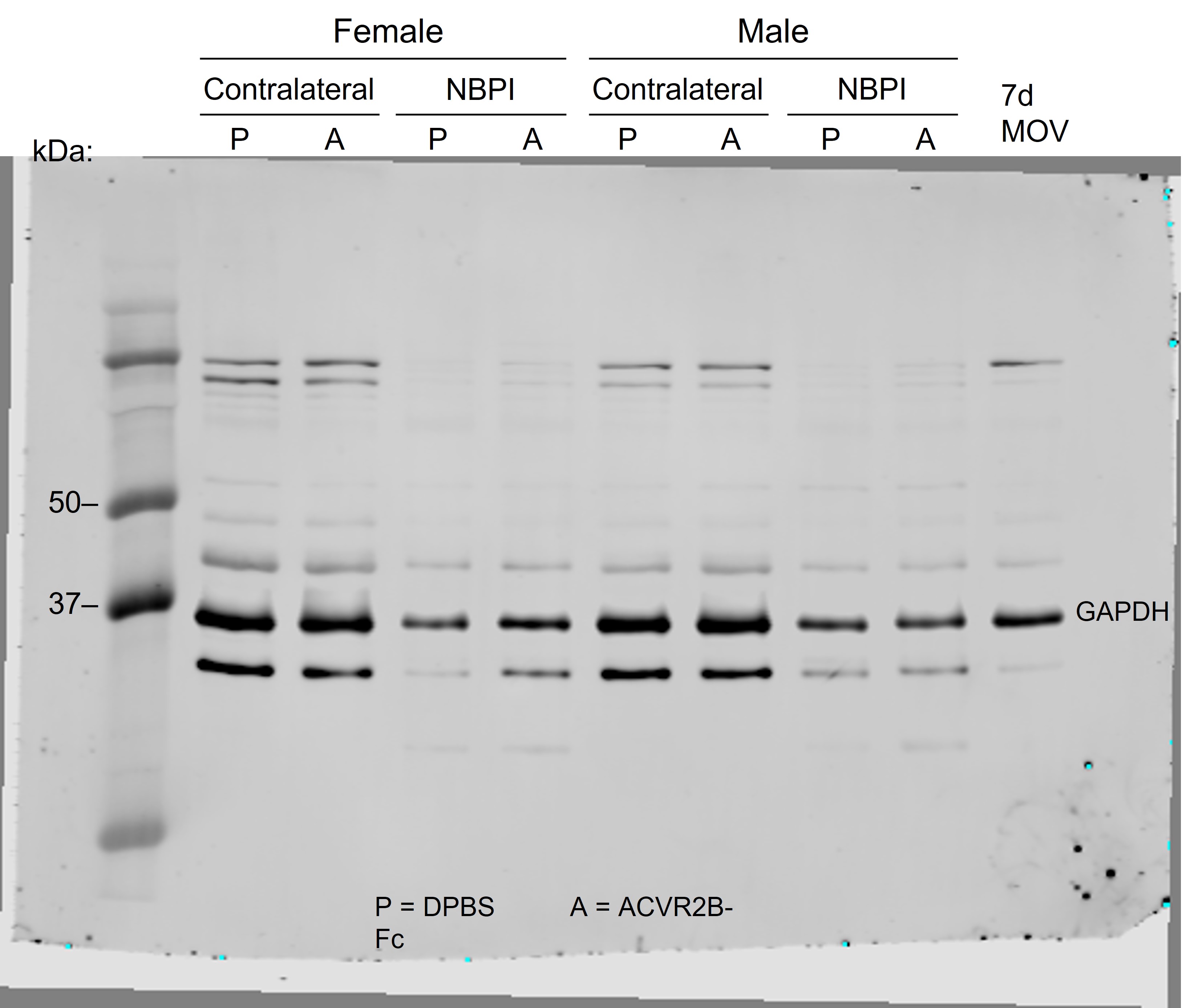

Supplement: Figure 6—figure supplement 1—source data 3. [file elife-81121-fig6-figsupp1-data3.zip › Figure 6-figure supplement 1-source data 3/Figure 6-figure supplement 1a GAPDH original.jpg]

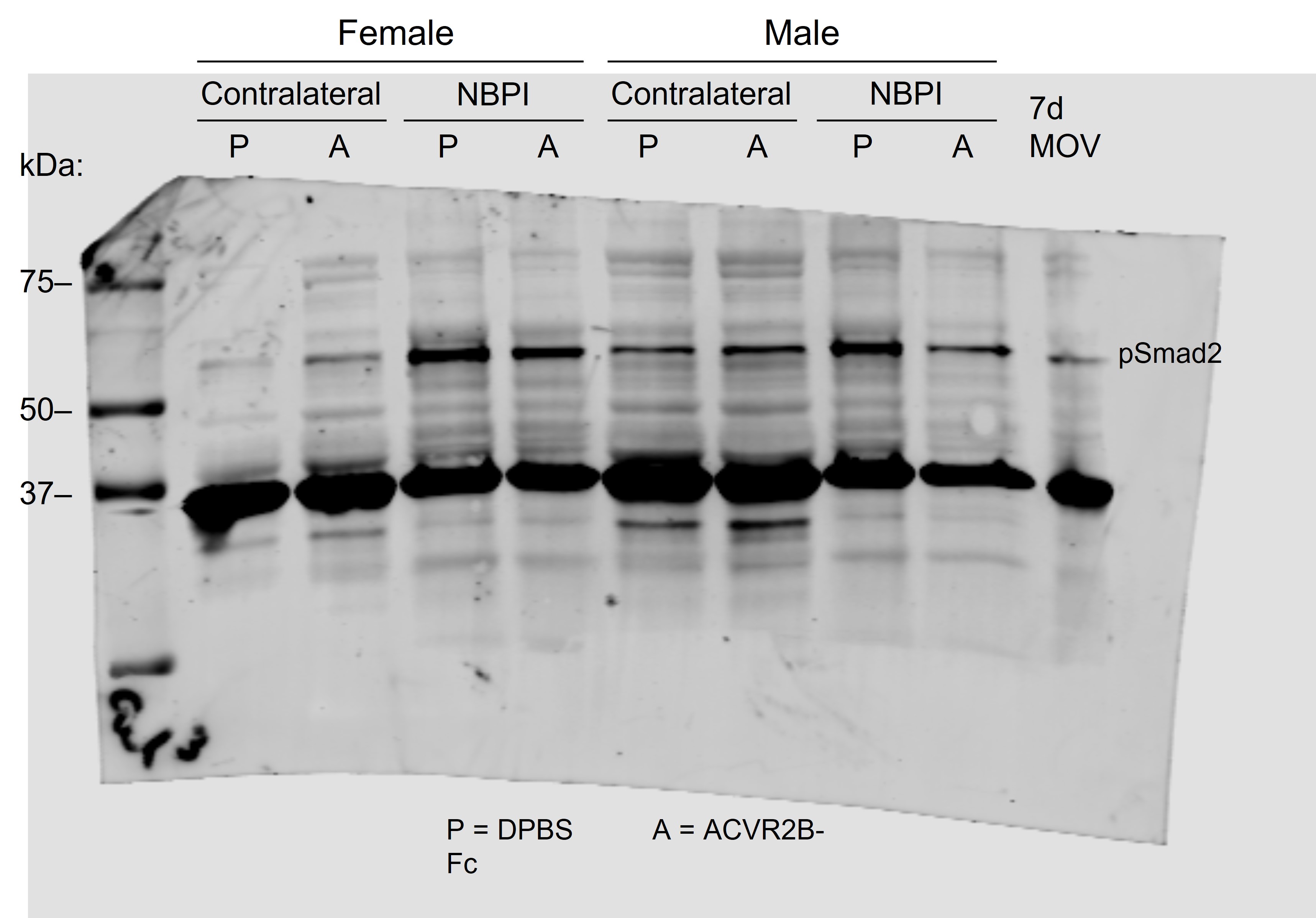

Supplement: Figure 7—source data 1. [file elife-81121-fig7-data1.zip › Figure 7-source data 1/Fig 7a pSmad2 original.jpg]

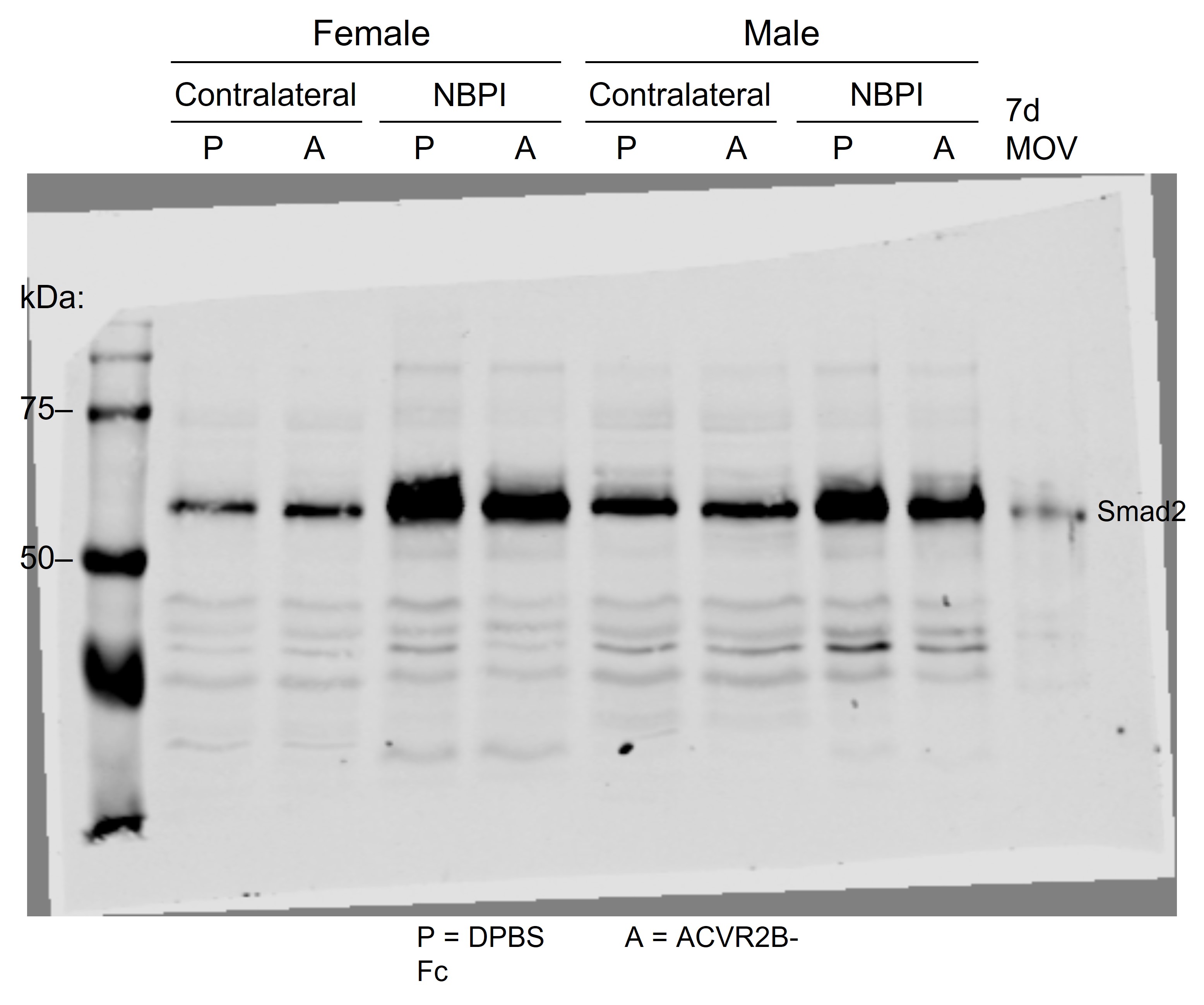

Supplement: Figure 7—source data 2. [file elife-81121-fig7-data2.zip › Figure 7-source data 2/Fig 7a Smad2 original.jpg]

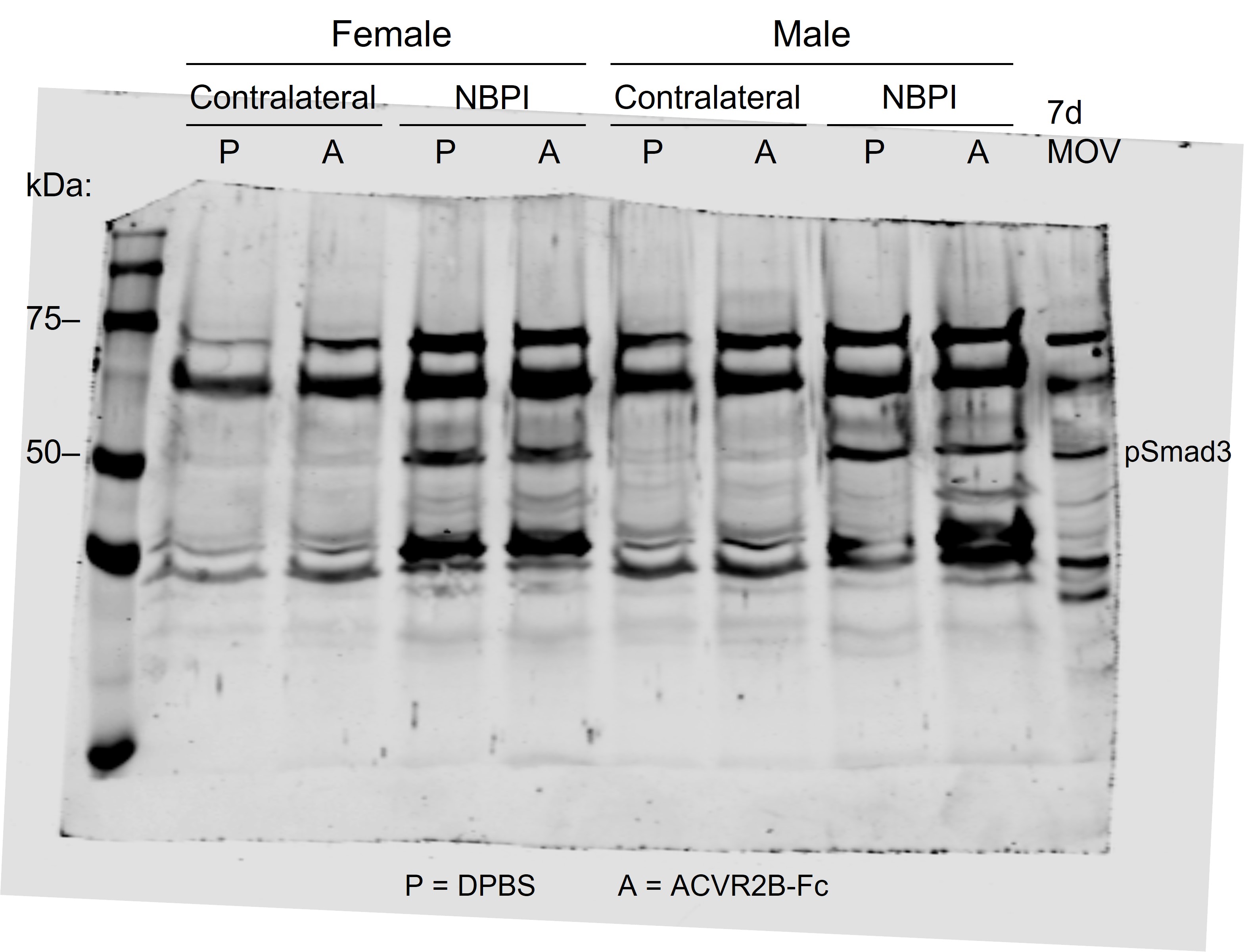

Supplement: Figure 7—source data 3. [file elife-81121-fig7-data3.zip › Figure 7-source data 3/Figure 7a pSmad3 original.jpg]

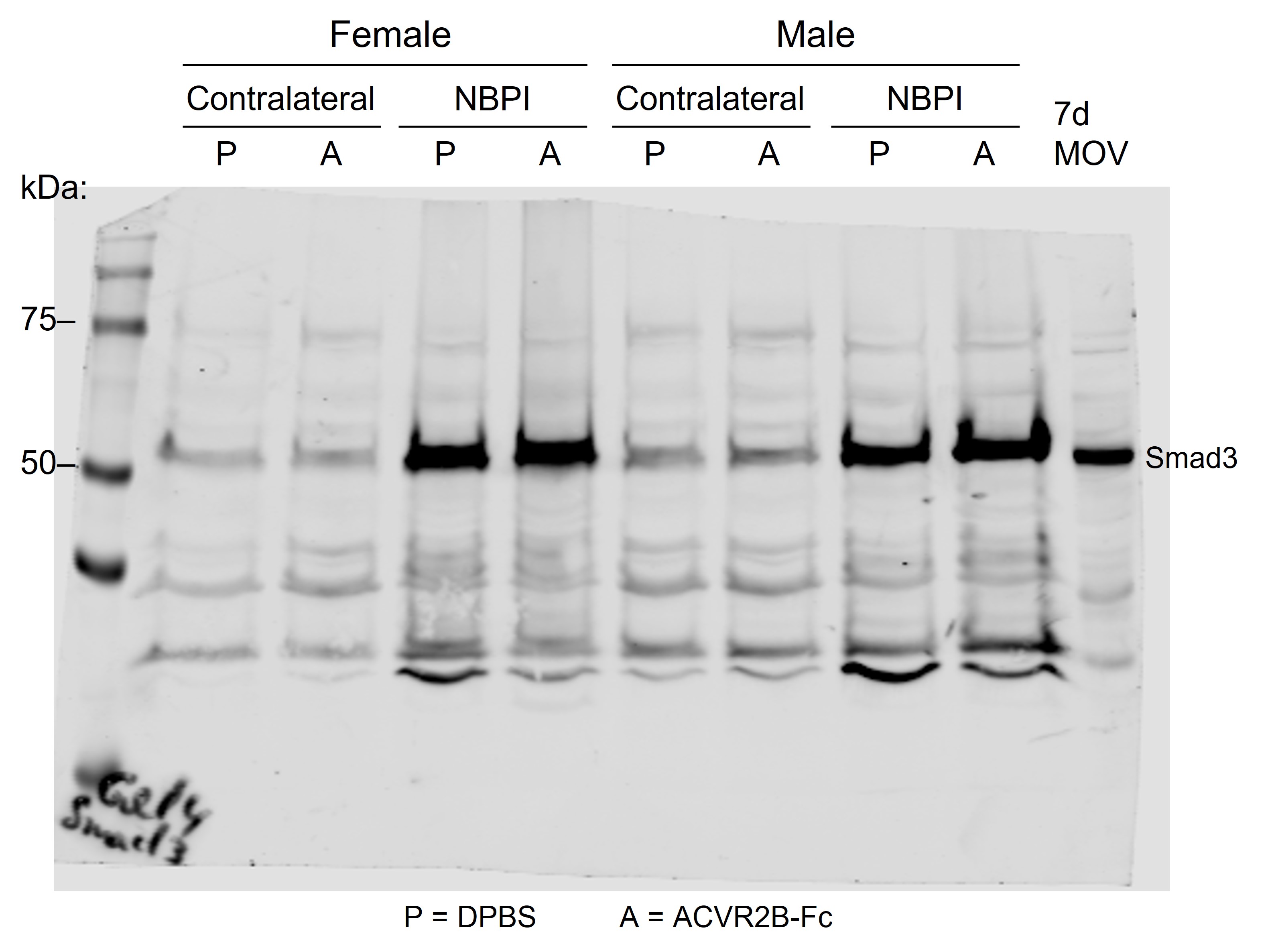

Supplement: Figure 7—source data 4. [file elife-81121-fig7-data4.zip › Figure 7-source data 4/Figure 7a Smad3 original.jpg]

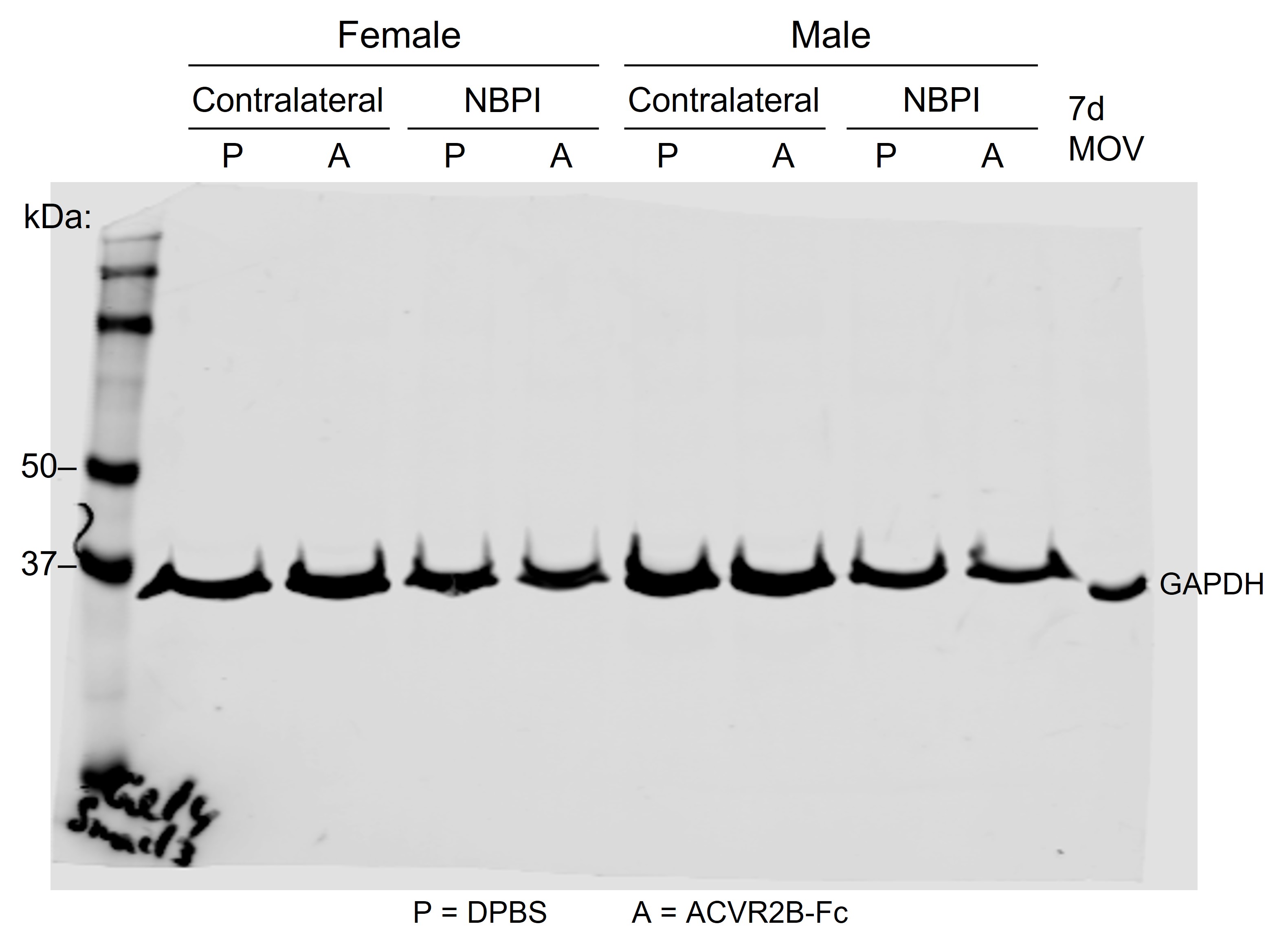

Supplement: Figure 7—source data 5. [file elife-81121-fig7-data5.zip › Figure 7-source data 5/Figure 7a GAPDH original.jpg]
