# Supplementary material for: Sex-specific role of myostatin signaling in neonatal muscle growth, denervation atrophy, and neuromuscular contractures
Source: eLife. 2022 Oct 31;11:e81121. doi: 10.7554/eLife.81121 (PMC9873256; doi:10.7554/eLife.81121)
Supplement: Figure 7—source data 5. [file elife-81121-fig7-data5.zip › Figure 7-source data 5 legend.docx]

**Figure 7A – GAPDH:** This file contains the full raw unedited gel scanned to Image Studio^TM^ Lite as well as the uncropped gel saved as a JPG file.
