# Supplementary material for: Sex-specific role of myostatin signaling in neonatal muscle growth, denervation atrophy, and neuromuscular contractures
Source: eLife. 2022 Oct 31;11:e81121. doi: 10.7554/eLife.81121 (PMC9873256; doi:10.7554/eLife.81121)
Supplement: Peer review history

# Editor report: Editor's evaluation

**DOI:** 10.7554/eLife.81121.sa0

**Authors:** Christopher Cardozo

**Affiliations:** https://ror.org/04a9tmd77 Icahn School of Medicine at Mount Sinai United States

This paper will be of interest to scientists within the field of neuromuscular disorders and has potential clinical relevance. The authors test a novel targeted strategy to reduce the sequelae of neonatal brachial plexus injury in a mouse model. The key claims of the manuscript are well supported by the data, and the approaches used are thoughtful and rigorous.

# Decision letter

**DOI:** 10.7554/eLife.81121.sa1

**Affiliations:** https://ror.org/04a9tmd77 Icahn School of Medicine at Mount Sinai United States; https://ror.org/04a9tmd77 Icahn School of Medicine at Mount Sinai United States

Our editorial process produces two outputs: (i) public reviews designed to be posted alongside the preprint for the benefit of readers; (ii) feedback on the manuscript for the authors, including requests for revisions, shown below. We also include an acceptance summary that explains what the editors found interesting or important about the work.

**Decision letter after peer review:**

Thank you for submitting your article "Sex-Specific Role of Myostatin Signaling in Neonatal Muscle Growth, Denervation Atrophy, and Neuromuscular Contractures" for consideration by *eLife*. Your article has been reviewed by 3 peer reviewers, including Christopher Cardozo as Reviewing Editor and Reviewer #1, and the evaluation has been overseen Mone Zaidi as the Senior Editor.

The reviewers have discussed their reviews with one another, and the Reviewing Editor has drafted this to help you prepare a revised submission.

Essential revisions:

1) The reviewers have raised a number of important points that must be addressed to improve the clarity and completeness of the manuscript. Some of these were raised by more than one reviewer. Please carefully consider each of these points as you revise the manuscript.

*Reviewer #1 (Recommendations for the authors):*

The original description of the ActRIIB-Fc ligand trap from Acceleron reported on ligands bound by the drug which included Activin A and, possibly GDF11. The specificity of the test agent should be discussed.

The authors should comment on whether their data on K48 polyubiquitination and proteasomal catalytic activity are internally consistent. Generally, reductions in proteasome catalytic activity lead to the accumulation of polyubiquitinated substrates unless the substrate is one of the small number of proteins that are degraded or processed without prior ubiquitination.

When interpreting the data on proteasomal activity, the authors should discuss the possible factors that might explain why caspase-like or chymotrypsin-like activities would change independently. One would expect these to change in a coordinated manner if they were simply due to changes in the total content of the 20S proteasome. However, these substrates are most likely also cleaved by subunits of the immunoproteasome though with different kinetics. Proteasomes exhibit both cooperativity and extreme sensitivity to allosteric regulators such as the protein complexes described by Rechsteiner and colleagues and sodium dodecyl sulfate.

*Reviewer #2 (Recommendations for the authors):*

In the Introduction section:

– Lines 73-74: The authors state that NBPI occurs in approximately 1.5 of every 1,000 live births. Because the article focuses partly on sex dimorphism, the reviewer wonders if the authors could provide epidemiological data about girls and boys.

– Lines 107-109: The authors state "In normally innervated skeletal muscles, ligand binding of MSTN to Activin A through its Type 2 receptors (ACVR and ACVR2B) activates the downstream Smad proteins 2 and 3". The reviewer thinks that there is a mistake. To its best knowledge, the reviewer thinks that Mstn does not bind to Activin A. Mstn and Activin A are able to bind to the same receptor though. The reviewer suggests revising it.

– Line 109: Please replace "ACVR" with "ACVR2". The reviewer suggests adding that ACVR is also known as ActRIIA and that ACVR2B is also known as ActRIIB.

– Line 114: The authors state that "it is possible that MSTN is a signaling pathway by which denervation impairs muscle growth". The reviewer wonders what the links would be between MSTN, denervation, and contractures, and how it could impede muscle growth.

– Line 121: In the sentence "to inhibit ligand binding of MSTN to Activin A", the reviewer feels there is a mistake/typo and does not clearly understand. Did the authors want to write "MSTN or Activin A"? Did the authors want to write "MSTN to ACVR2B"? The reviewer suggests rephrasing it.

In the Results section:

– Line 143: The authors state that it "has been shown to induce robust muscle growth in adult mice". The reviewer wonders if the authors could provide an appropriate reference for this statement.

– Line 153/Figure 1G: The reviewer wonders why the total protein levels in the biceps muscle were not normalized with muscle weight, because usually if the muscle is bigger, the total protein levels should be higher (same question for Figure 2B).

– Lines 157-158: The authors state that "it facilitates greater protein anabolism and growth in female muscles.". While the data looks promising and show a sex difference, the reviewer thinks that the figures do not show an effect on protein anabolism itself. The authors should have a moderate view of the subject and revise this sentence accordingly.

– Line 161/Supplemental Figure 1B and line 176/Supplemental Figure 2: The reviewer wonders how the authors could explain the same effects observed on the humerus length of the innervated side as the denervated side (in both females and males). The reviewer wonders also what the literature does say about the (pharmacologic or non-pharmacologic) blockade of MSTN signaling and bone growth, and what links could be made with the situation described here.

– Lines 160-161: The reviewer feels that "(Figure 1 – supplemental figures 1A-D)" is a bit confusing. The reviewer suggests renaming it Supplemental Figures 1A-D.

– Lines 175-176: The reviewer feels that "(Figure 2 – supplemental figure 1A)" is a bit confusing. The reviewer suggests renaming it as Supplemental Figure 2.

– Figure 5: While the whole assessment of MSTN inhibition on muscle mass, muscle CSA, muscle volume, muscle protein content, and sarcomere length was performed mainly on the Brachialis muscle (and Biceps long head), the reviewer wonders why it was not used for the biochemical analysis of protein synthesis and its associated signaling pathway. While Brachialis and Biceps are the flexors, Triceps is an extensor, therefore could the authors explain why they choose to use the Triceps muscle instead?

Also, the reviewer wonders why a part of the analysis is made on the Biceps long head and the other part on the Triceps muscle. How the authors can be sure that these muscles behave the same way in this context? The reviewer feels that for more precision and reliability the authors should have used the same muscle.

– Figure 5F: It clearly shows an increase in the phosphorylation level of Akt in control females compared to control males. The reviewer wonders how the authors could explain this difference. What would be their hypothesis?

– Figure 6: While the whole assessment of MSTN inhibition on muscle mass, muscle CSA, muscle volume, muscle protein content, and sarcomere length was performed mainly on the Brachialis muscle (and Biceps long head), the reviewer wonders why it was not used for the biochemical analysis of protein degradation. While Brachialis and Biceps are the flexors, Triceps is an extensor, therefore could the authors explain why they choose to use the Triceps muscle instead?

Also, the reviewer wonders why a part of the analysis is made on the Biceps long head and the other part on the Triceps muscle. How the authors can be sure that these muscles behave the same way in this context? The reviewer feels that for more precision and reliability the authors should have used the same muscle.

– Lines 220-223: The authors state "Since MSTN is known to activate the ubiquitin-proteasome pathway by upregulating the expression of upstream ubiquitin ligases,30,31 it was surprising that its inhibition did not reduce ubiquitination levels.". Based on their statement, the reviewer wonders why the authors did not assess the mRNA and protein levels of them. For example, *Murf1* and Atrogin-1. The reviewer suggests the authors provide this detailed information for a better comprehension of the hidden mechanisms.

– Figure 7: While the whole assessment of MSTN signaling inhibition on muscle mass, muscle CSA, muscle volume, muscle protein content, and sarcomere length was performed mainly on the Brachialis muscle (and Biceps long head), the reviewer wonders why it was not used for the biochemical analysis of the MSTN signaling pathway. While Brachialis and Biceps are the flexors, Triceps is an extensor, therefore could the authors explain why they choose to use the Triceps muscle instead?

Also, the reviewer wonders why the analysis is made on the Triceps muscle alone. How the authors can be sure that these muscles behave the same way in this context? The reviewer feels that for more precision and reliability the authors should have used the same muscle.

– Figure 7: In order to characterize the Smad2/3 pathway, the authors investigated the Smad3 phosphorylation level. Due to prior literature in the field, the reviewer wonders why the authors did not assess Smad2 phosphorylation level, in order to get a fully explained picture of the situation. The reviewer suggests the authors provide this detailed information for a better comprehension of the hidden mechanisms.

– Lines 232-234: The authors state "Our collective findings thus posit a sex-specific role for MSTN in muscle proteostasis dysregulation after neonatal denervation through non-canonical signaling pathways."? While the reviewer agrees based on the data provided, the reviewer wonders why the authors did not investigate further in that direction. The reviewer wonders how the *TAK1* pathway and its downstream targets such as *JNK* or p38 are regulated. The reviewer also wonders how the Ras pathway and its downstream targets such as ERK1/2 are regulated.

– It is known that MSTN has inhibitory effects on the Wnt/β-catenin pathway, thereby blunting satellite cell proliferation. Besides Wnt/β-catenin signaling plays a crucial role in myoblast fusion. In this context of MSTN signaling inhibition, the reviewer wonders how this Wnt/β-catenin signaling is regulated and what sort of effect it can have on longitudinal muscle growth.

– Lines 245: The authors wrote "(Figures 8E-F)", but they forgot to call Figures 8G-H in the manuscript. The reviewer suggests revising it accordingly.

In the Discussion section:

– Line 272: The authors state "we pharmacologically targeted MSTN". While this is true, the authors cannot forget that it targets also the signaling mediated by Activin A. The reviewer thinks that it should appear in the whole manuscript.

– Line 333: Please replace "NPBI" with "NBPI".

– Lines 361-362 to 372: The authors state "These discrepancies might be attributable to sex-related differences of MSTN expression itself in skeletal muscles.". The reviewer wonders how the MSTN, Activin A, ACVR2, and ACVR2B expression is, in both females and males here. Also, the reviewer wonders what is the level of expression of MSTN, Activin A, and ACVR2 and ACVR2B expression in the Brachialis, Biceps, and Triceps. Could the authors imagine that there is also a muscle-dependent regulation?

In a previous study, the authors looked at the effects of bortezomib over different periods (4, 8, and 12 weeks). Here, the authors studied the effects of ACVR2B-Fc treatment over a period of 4 weeks. The reviewer wonders why they did not repeat the same durations of experimentation. The reviewer thinks it would be helpful to have this detailed information for having a better comprehension of the observed effect.

Also, the reviewer wonders what the effect of combining bortezomib and ACVR2B-Fc treatment would be. What would the authors hypothesize?

In the Materials and methods section:

– Lines 456-458: The authors state "The binding of MSTN to the Fc domain inhibits MSTN from binding to Activin A, which blocks MSTN activity in the muscle". The reviewer thinks that it should be ACVR2B instead of Activin A. The reviewer suggests revising it.

– Line 495: Please replace "micro-computed tomography" with "micro-computed tomography (MicroCT)".

– Line 550: Please replace "PVDF" with "polyvinylidene fluoride (PVDF)".

– Line 553: Please replace "BSA" with "bovine serum albumin (BSA)".

– Lines 569-570: Please replace "β-1" with "β1".

– Line 570: Please replace "β-5" with "β5".

– Line 580: Please replace "SDS-PAGE" with "sodium dodecyl sulfate-polyacrylamide gel electrophoresis (SDS-PAGE)".

– Line 598: Please replace "ANOVA" with "analysis of variance (ANOVA)".

– Line 602: Please replace "SD" with "standard deviation (SD)".

– Line 613: Please replace "ARRIVE" with "Animal Research: Reporting of in vivo Experiments (ARRIVE)".

In the Figure Legends section:

– Line 650: Please replace "micro-CT" with "MicroCT".

– Lines 656-658: The reviewer feels that (E) and (H) are misplaced in the sentence "Despite this, (E), (H) the increases in muscle volume, muscle weight, and protein levels were larger in females than males when compared to their respective DPBS controls."

– Line 663: Please replace "micro-CT" with "MicroCT".

# Reply: Author response

**DOI:** 10.7554/eLife.81121.sa2

Essential revisions:

1) The reviewers have raised a number of important points that must be addressed to improve the clarity and completeness of the manuscript. Some of these were raised by more than one reviewer. Please carefully consider each of these points as you revise the manuscript.

Reviewer #1 (Recommendations for the authors):

The original description of the ActRIIB-Fc ligand trap from Acceleron reported on ligands bound by the drug which included Activin A and, possibly GDF11. The specificity of the test agent should be discussed.

The ActRIIB-Fc ligand trap is indeed capable of binding different ligands, including Activin A and GDF11. This broad specificity confers both advantages and adverse effects – the ligand trap is capable of eliciting more robust muscle growth than MSTN-specific agents while concomitantly increasing the risk of toxicity to other tissues and cell types. Given this, we have included a discussion on the specificity of the ligand trap in our revised manuscript, and provided appropriate sources for additional clarification.

Discussion (lines #479-481): “Indeed, the broad ligand specificity of ACVR2B-Fc increases the risk of toxicity to non-skeletal muscle tissues and cell types, ranging from epistaxis to telangiectasias.^69^”

Discussion (lines #511-531): “For a more nuanced interpretation of our findings, we must also consider the specificity of the ACVR2B-Fc decoy receptor as a ligand trap, as mentioned above. Previous studies have reported that many members of the TGF-β superfamily, such as Activin A and GDF-11, are capable of binding Activin Type IIB receptors.^74,75^ This broad array of targeted ligands makes the decoy receptor a potent inhibitor of not only MSTN, but also an inhibitor of various TGF-β family members that signal downstream of Activin binding. Of note, simultaneous inhibition of MSTN and Activin A leads to more effective muscle hypertrophy and force production in rodents and primates.^76^ Consequently, this ligand trap putatively elicits more robust increases in skeletal muscle growth than MSTN specific-agents.^19^ In our current study, it is possible that Activin A is differentially expressed and/or blocked with ACVR2B-Fc between sexes, which may account for the sex dimorphisms observed in neonatally denervated muscles. Indeed, a sex-specific role for Activin A in pancreatic ductal adenocarcinoma (PDAC)induced cachexia has recently been reported by Zhong et al. (2022). The authors observed that sex-specific differences in endogenous levels of Activin A contribute to sex dimorphisms in PDAC-induced cachexia, as well as the differential outcomes of ACVR2BFc treatment in attenuating tumor-induced Activin.^77^ Alternatively, we also wonder whether Activin A and/or other TGF-β family members are more acutely involved in the regulation of neonatal longitudinal muscle growth and contractures than MSTN. Future work with genetic models and expression profiles for the various TGF-β superfamily members are necessary to address these limitations in our current study.”

The authors should comment on whether their data on K48 polyubiquitination and proteasomal catalytic activity are internally consistent. Generally, reductions in proteasome catalytic activity lead to the accumulation of polyubiquitinated substrates unless the substrate is one of the small number of proteins that are degraded or processed without prior ubiquitination.

We agree that our data on unaltered K48 polyubiquitination and reduced proteasomal catalytic activity in female mice after treatment do appear paradoxical, but as the reviewer pointed out, they are not without precedent. The phenomenon of ubiquitin-independent proteasomal degradation has been supported by several prior studies in a small number of proteins. These proteins share common features of belonging to the Intrinsic Disordered Protein (IDP) family and possessing intrinsically disordered regions (IDR). We have expanded our discussion to include this possibility.

Discussion (lines #342-354): “Our seemingly paradoxical finding of reduced proteasomal catalytic activity in the absence of any alterations in K48 polyubiquitin is not without precedent. Indeed, the phenomenon of ubiquitin-independent proteasomal degradation has been supported by several prior studies in a subset of proteins, which share common features of belonging to the Intrinsic Disordered Protein (IDP) family,^46,47^ such as p21 and p53.^48-50^ Since these proteins and their associated intrinsically disordered regions (IDRs) critically regulate various cellular processes, including transcriptional regulation, posttranslational modification, and molecular signaling,^51^ it is possible that neonatal muscle denervation induces a direct degradation of these substrates. If so, the sex-specific reduction in proteasome catalytic activity with MSTN inhibition may be due to the preservation of these IDPs, which would not lead to an accumulation of polyubiquitinated substrates. It remains unclear what specific protein substrates are polyubiquitinated with NBPI, and future studies are needed to address this question.”

When interpreting the data on proteasomal activity, the authors should discuss the possible factors that might explain why caspase-like or chymotrypsin-like activities would change independently. One would expect these to change in a coordinated manner if they were simply due to changes in the total content of the 20S proteasome. However, these substrates are most likely also cleaved by subunits of the immunoproteasome though with different kinetics. Proteasomes exhibit both cooperativity and extreme sensitivity to allosteric regulators such as the protein complexes described by Rechsteiner and colleagues and sodium dodecyl sulfate.

We thank the reviewer for raising this point about coordinated changes among the proteasome subunits. We cannot be certain whether the enzymatic changes arise due to total changes in proteasome content, or reflect changes in affinities to specific proteins involved in muscle longitudinal growth and sarcomere addition. There have also been contrasting reports on whether the subunits act independently or in synchrony. We have updated our discussion to acknowledge both possibilities, as well as the potential of different subunit affinities for specific protein substrates.

Discussion (lines #375-393): “Alternatively, the independent alterations in caspase-like and chymotrypsin-like activities could potentially reflect different affinities of the two enzymatic processes for specific sarcomeric, cytoskeletal, or regulatory proteins involved in muscle growth and sarcomere addition. This notion is supported by Kisselev et al. (2006), who showed that the unique amino acid sequence of a protein substrate differentially determines the activities of the 20S proteolytic sites during protein degradation, thereby leading to variability in efficacy of the different inhibitors.^52^ Importantly, mutations and crystallography of yeast 20S proteasome by Groll et al. (1999) revealed that the three active β-subunits are processed autocatalytically and independently of each other, with the processing of one subunit unaffected by the inactivation of another subunit.^53^ The independent processing function of the individual subunits, along with their unique affinities for specific protein substrates, may explain the independent changes in enzymatic activity observed in our current and prior studies. Although, we acknowledge that our findings contradict observations of allosteric interactions among the active subunits observed by Kisselev et al. (1999), specifically between the caspase-like and chymotrypsin-like sites and vice versa, in eukaryotic proteasomes.^54^ Additional studies are required to reconcile the independent changes of the subunits with total changes in proteasome content, as we continue to develop insights on proteasome function in contracture pathophysiology.”

Regarding the 20S immunoproteasome, we did observe an accompanying decrease in β1i activity in the absence of any changes in β5i activity, in our prior study with bortezomib treatment (data not reported). We did not assess these activities in our current study. To our knowledge, the overall abundance of the immunoproteasome is low in non-lymphoid tissues, and requires induction by cytokines. As such, we also cannot be certain whether the immunoproteasome contributes to the discrepancies, nor can we discount its role. Finally, we thank the reviewer for bringing to our attention the cooperativity and sensitivity exhibited by proteasomes to allosteric regulators, such as the proteasome activator PA200 described by Rechsteiner and colleagues (2002). We speculate that these characteristics were not observed in our current study potentially because MSTN acts upstream of the ubiquitin-proteasome system. Thus, we suspect the decoy receptor effects upstream changes in signaling that regulates proteasome subunit activity independently, rather than binding to the site of an allosteric enzyme on the subunit as an allosteric effector would. As such factors are highly speculative at this point, we would prefer not to include them in the manuscript.

Reviewer #2 (Recommendations for the authors):

In the Introduction section:

– Lines 73-74: The authors state that NBPI occurs in approximately 1.5 of every 1,000 live births. Because the article focuses partly on sex dimorphism, the reviewer wonders if the authors could provide epidemiological data about girls and boys.

This is a relevant question given our discovery of sex dimorphisms in the pathophysiology of contracture formation. The ratio of NBPI occurrence between girls and boys is approximately 1:1 (McLaren et al., 2019). We have included this epidemiological data in our revised Introduction.

Introduction (lines #72-74): “Injury to the brachial plexus at birth (Neonatal Brachial Plexus Injury – NBPI) is the most common cause of upper limb paralysis in children,^1^ occurring in approximately 1.5 of every 1,000 live births, in both females and males at an equal rate.^2”^

– Lines 107-109: The authors state "In normally innervated skeletal muscles, ligand binding of MSTN to Activin A through its Type 2 receptors (ACVR and ACVR2B) activates the downstream Smad proteins 2 and 3". The reviewer thinks that there is a mistake. To its best knowledge, the reviewer thinks that Mstn does not bind to Activin A. Mstn and Activin A are able to bind to the same receptor though. The reviewer suggests revising it.

– Line 109: Please replace "ACVR" with "ACVR2". The reviewer suggests adding that ACVR is also known as ActRIIA and that ACVR2B is also known as ActRIIB.

The reviewer is correct in saying that MSTN and Activin A are capable of binding to the ACVR2/ACVR2B receptor, as they are both members of the TGF-β superfamily. We have corrected this error, replaced “ACVR” with “ACVR2”, and included the alternative names of ActRIIA and ActRIIB in our revised Introduction.

Introduction (lines #109-112): “In normally innervated skeletal muscles, ligand binding of MSTN to its Type 2 receptors, ACVR2 (ActRIIA) and ACVR2B (ActRIIB), activates the downstream Smad proteins 2 and 3.^16,17”^

– Line 114: The authors state that "it is possible that MSTN is a signaling pathway by which denervation impairs muscle growth". The reviewer wonders what the links would be between MSTN, denervation, and contractures, and how it could impede muscle growth.

We have revised our Introduction to improve the clarity of the links between MSTN, denervation, and contractures.

Introduction (lines #117-124): “As mentioned, we previously discovered that neonatal denervation increases protein degradation in denervated muscles,^13^ which leads to impaired longitudinal muscle growth that ultimately causes contractures.^9-12^ However, the mechanism by which neonatal denervation increases protein degradation is unknown. Given that MSTN is a potent inhibitor of muscle growth through its mediation of muscle proteostasis, we speculate that MSTN signaling may be a mechanistic pathway linking denervation with protein degradation, leading to impaired muscle growth and ultimately contractures.”

– Line 121: In the sentence "to inhibit ligand binding of MSTN to Activin A", the reviewer feels there is a mistake/typo and does not clearly understand. Did the authors want to write "MSTN or Activin A"? Did the authors want to write "MSTN to ACVR2B"? The reviewer suggests rephrasing it.

Thank you for bringing this to our attention. We indeed meant to write “MSTN to ACVR2B,” as noted by the reviewer.

Introduction (lines #130): “Using a soluble decoy receptor (ACVR2B-Fc) to inhibit ligand binding of MSTN to ACVR2B,^20-23^ we specifically investigated whether pharmacologic inhibition of MSTN signaling preserves longitudinal muscle growth and prevents contractures after NBPI.”

In the Results section:

– Line 143: The authors state that it "has been shown to induce robust muscle growth in adult mice". The reviewer wonders if the authors could provide an appropriate reference for this statement.

We have included the appropriate citation for this statement (Lee et al., 2005).

Results (lines #150): “While pharmacologic inhibition of MSTN signaling with the soluble ACVR2B-Fc decoy receptor has been shown to induce robust muscle growth in adult mice,^20^ it has not been validated in neonatal mice.”

– Line 153/Figure 1G: The reviewer wonders why the total protein levels in the biceps muscle were not normalized with muscle weight, because usually if the muscle is bigger, the total protein levels should be higher (same question for Figure 2B).

To our knowledge, total muscle protein content is a fairly standard marker of muscle hypertrophy (Marino et al., 2008; Dearth et al., 2013). The reviewer is correct that total protein levels should generally track with muscle weight, thus it serves as an additional readout to compare muscle growth. We have analyzed protein levels according to muscle weight as recommended, and recorded the new observations in our revised manuscript. Normalized protein levels were not changed with MSTN inhibition in either normally innervated or denervated muscles, although treatment led to higher levels in denervated female vs. male muscles.

Results (lines #160-162): “There were no changes in protein levels normalized to muscle weight with treatment (Figure 1—figure supplement 1A).”

Results (lines #183-186): “Additionally, while MSTN inhibition did not elevate protein levels normalized to muscle weight in denervated muscles beyond the respective sexspecific controls, it led to higher levels in female than male muscles (Figure 2—figure supplement 1A).”

Figure legends (lines #904-906): “Figure 1—figure supplement 1: Off-target effects of pharmacologic MSTN inhibition. (A) MSTN inhibition does not alter protein levels in normally innervated biceps when normalized to muscle weight.”

Figure legends (lines #913-916): “Figure 2—figure supplement 1: Effect of pharmacologic MSTN inhibition on normalized protein levels and skeletal growth in denervated forelimb. (A) MSTN inhibition does not alter protein levels in denervated biceps when normalized to muscle weight.”

– Lines 157-158: The authors state that "it facilitates greater protein anabolism and growth in female muscles.". While the data looks promising and show a sex difference, the reviewer thinks that the figures do not show an effect on protein anabolism itself. The authors should have a moderate view of the subject and revise this sentence accordingly.

We thank the reviewer for this cautionary advice and have revised the sentence accordingly.

Results (lines #167): “These results indicate that while neonatal inhibition of MSTN signaling effectively enhances skeletal muscle growth in both sexes, it facilitates greater increases in protein content and muscle weight in female muscles.”

– Line 161/Supplemental Figure 1B and line 176/Supplemental Figure 2: The reviewer wonders how the authors could explain the same effects observed on the humerus length of the innervated side as the denervated side (in both females and males). The reviewer wonders also what the literature does say about the (pharmacologic or non-pharmacologic) blockade of MSTN signaling and bone growth, and what links could be made with the situation described here.

We thank the reviewer for bringing these observations to our attention. We interpret the collective results here to indicate that ACVR2B-Fc attenuates neonatal skeletal growth exclusively in male mice, adding to the list of sex dimorphisms reported in our study. We state this finding more explicitly in our revised manuscript.

Results (lines #189-192): “Similar to the contralateral forelimbs, MSTN inhibition in male mice resulted in an attenuation of skeletal growth in the denervated humerus (Figure 2— figure supplement 1B). These parallel observations suggest that ACVR2B-Fc attenuates neonatal skeletal growth exclusively in male mice.”

Regarding prior literature, Hamrick and colleagues (2002) found that embryonic genetic deletion of MSTN affected humerus morphology but not length in adult mice. However, these authors used a mixed-sex approach that may have masked sex-specific effects of MSTN deletion. We have revised our manuscript to include this prior literature.

Discussion (lines 497-500): “Prior studies have failed to find an effect of embryonic MSTN deletion on humerus length in adult mice, although the use of a mixed-sex approach may have masked sex-related differences.^71”^

Beyond comparison with prior findings, the deficits in bone growth of male mice strengthens the sex-specific phenotype we observed with MSTN inhibition. We would like to take this opportunity to reiterate a point we had discussed in our original manuscript that, biomechanically, a shorter bone should reduce contracture severity and result in an increase of normalized muscle size. The absence of such findings in male mice thus further underscores the sex-specificity of MSTN inhibition in denervated muscles (lines #491-497).

– Lines 160-161: The reviewer feels that "(Figure 1 – supplemental figures 1A-D)" is a bit confusing. The reviewer suggests renaming it Supplemental Figures 1A-D.

– Lines 175-176: The reviewer feels that "(Figure 2 – supplemental figure 1A)" is a bit confusing. The reviewer suggests renaming it as Supplemental Figure 2.

While we concur with the reviewer’s feedback, we are unable to adopt this suggestion due to specific instructions from the editorial staff on naming our supplemental figures. We thank the reviewer for his/her understanding in this matter.

Results (lines #169-170): (Figure 1—figure supplement 1B-E)

Figure Legends (line #904-905): Figure 1—figure supplement 1: Off-target effects of pharmacologic MSTN inhibition.

Results (line #186): (Figure 2—figure supplement 1A)

Figures Legends (line #913-915): Figure 2—figure supplement 1: Effect of pharmacologic MSTN inhibition on normalized protein levels and skeletal growth in denervated forelimb.

– Figure 5: While the whole assessment of MSTN inhibition on muscle mass, muscle CSA, muscle volume, muscle protein content, and sarcomere length was performed mainly on the Brachialis muscle (and Biceps long head), the reviewer wonders why it was not used for the biochemical analysis of protein synthesis and its associated signaling pathway. While Brachialis and Biceps are the flexors, Triceps is an extensor, therefore could the authors explain why they choose to use the Triceps muscle instead?

Also, the reviewer wonders why a part of the analysis is made on the Biceps long head and the other part on the Triceps muscle. How the authors can be sure that these muscles behave the same way in this context? The reviewer feels that for more precision and reliability the authors should have used the same muscle.

– Figure 6: While the whole assessment of MSTN inhibition on muscle mass, muscle CSA, muscle volume, muscle protein content, and sarcomere length was performed mainly on the Brachialis muscle (and Biceps long head), the reviewer wonders why it was not used for the biochemical analysis of protein degradation. While Brachialis and Biceps are the flexors, Triceps is an extensor, therefore could the authors explain why they choose to use the Triceps muscle instead?

Also, the reviewer wonders why a part of the analysis is made on the Biceps long head and the other part on the Triceps muscle. How the authors can be sure that these muscles behave the same way in this context? The reviewer feels that for more precision and reliability the authors should have used the same muscle.

– Lines 220-223: The authors state "Since MSTN is known to activate the ubiquitin-proteasome pathway by upregulating the expression of upstream ubiquitin ligases,30,31 it was surprising that its inhibition did not reduce ubiquitination levels.". Based on their statement, the reviewer wonders why the authors did not assess the mRNA and protein levels of them. For example, Murf1 and Atrogin-1. The reviewer suggests the authors provide this detailed information for a better comprehension of the hidden mechanisms.

– Figure 7: While the whole assessment of MSTN signaling inhibition on muscle mass, muscle CSA, muscle volume, muscle protein content, and sarcomere length was performed mainly on the Brachialis muscle (and Biceps long head), the reviewer wonders why it was not used for the biochemical analysis of the MSTN signaling pathway. While Brachialis and Biceps are the flexors, Triceps is an extensor, therefore could the authors explain why they choose to use the Triceps muscle instead?

Also, the reviewer wonders why the analysis is made on the Triceps muscle alone. How the authors can be sure that these muscles behave the same way in this context? The reviewer feels that for more precision and reliability the authors should have used the same muscle.

We appreciate the reviewer’s concern and thorough scrutiny of our findings. We agree that using the same muscle type for all physiological and biochemical analyses would ideally allow for greater precision. Our selection of the different forelimb muscles was ultimately based on both methodological constraints and tissue availability. As described in the Materials and methods, we digested the brachialis muscles to isolate fiber bundles for assessment of sarcomere length. Following this terminal physiological measurement, the brachialis muscle cannot be used for subsequent biochemical analyses. We therefore utilized the biceps (long head) muscle to analyze protein dynamics, specifically levels of protein synthesis and K48 polyubiquitination. However, the limited amounts of protein available in the denervated biceps precluded their use for assessment of signaling pathways and proteasome activity. We therefore used the triceps muscle for these latter analyses. Despite being an extensor as the reviewer pointed out, it is important to note that the triceps muscle is completely denervated in our surgical mouse model of postganglionic NBPI, which essentially denervates the entire forelimb. Thus, the triceps muscle is subjected to the same denervation condition as the elbow flexors. In our revised manuscript, we provide an explanation for our choice of the different forelimb muscles in the Materials and methods, and acknowledge that using the same muscles may improve the precision of our findings in the Discussion.

Discussion (lines #431-470): “First, the small size of our denervated muscles precluded the use of the same muscles for all analyses, instead requiring smaller subgroup sizes as well as different muscles for certain biochemical endpoints (Akt, Smad2/3, *Murf1*, and Atrogin-1). We therefore acknowledge that our study may be underpowered to detect smaller effects in certain parameters of protein dynamics, specifically signaling proteins and ubiquitin ligases. We also acknowledge that the precision of our findings would be further enhanced with the use of the same muscle type across all of our morphological, physiological, and biochemical analyses.”

Materials and methods (lines #565-566): “This type of injury completely denervates all muscles in the forelimb, including flexors (brachialis, biceps), and extensors (triceps).”

Materials and methods (lines #708-715): “Regarding the use of different muscles for analyses of protein dynamics compared to physiological endpoints, the dissection of fiber bundles for sarcomere length assessment described above prevents any subsequent processing of the brachialis. Furthermore, while the biceps long head was processed for puromycin incorporation and total levels of K48 polyubiquitin, the limited amounts of protein available in denervated biceps precluded their use for assessment of ubiquitin ligases and the 20S proteasome subunits. Consequently, triceps muscles were used to assess muscle-specific ubiquitin ligases, proteasome activity, and the various signaling pathways described below.”

– Figure 5F: It clearly shows an increase in the phosphorylation level of Akt in control females compared to control males. The reviewer wonders how the authors could explain this difference. What would be their hypothesis?

We thank the reviewer for this astute observation. Our finding here suggests that Akt signaling in normally innervated muscles is mediated in a sex-specific manner. This sex dimorphism in Akt signaling is not without precedent. Camper-Kirby and colleagues (2001) similarly reported elevated Akt signaling in the myocardium of young women and juvenile female mice (~6w) compared to young men and juvenile male mice, respectively. However, the mechanisms for these sex-specific differences may vary between muscles. In cardiac muscles of female mice, Camper-Kirby et al. reported a 2-fold increase in phosphorylated Akt levels when corrected for loading (P<0.01). Conversely, we observed a non-statistically significant 4-fold increase in corrected total Akt levels in skeletal muscles of male mice. As increased total levels of p70s6k (a protein kinase downstream of Akt/mTOR signaling) is potentially indicative of enhanced overall protein translation (Bakker et al., 2016), we speculate the increased level of Akt similarly points to increased protein production. This speculation is corroborated by the higher levels of total protein (Figure 1G) in normally innervated skeletal muscles of neonatal male mice. In this manner, sex dimorphisms in hypertrophic signaling pathways putatively account for the greater protein content and overall mass in male skeletal muscles. As this hypothesis is untested, we prefer to exclude the preceding discussion from our manuscript unless its inclusion is specifically requested.

– Lines 220-223: The authors state "Since MSTN is known to activate the ubiquitin-proteasome pathway by upregulating the expression of upstream ubiquitin ligases,30,31 it was surprising that its inhibition did not reduce ubiquitination levels.". Based on their statement, the reviewer wonders why the authors did not assess the mRNA and protein levels of them. For example, Murf1 and Atrogin-1. The reviewer suggests the authors provide this detailed information for a better comprehension of the hidden mechanisms.

We have performed western blots for both *Murf1* and Atrogin-1, and observed MSTN inhibition does not alter MuRF-1 or Atrogin-1 protein levels in neonatal skeletal muscles. Surprisingly, four weeks of denervation led to reduced *Murf1* expression but did not alter Atrogin-1 translation. Since we previously reported increased transcriptional activity of *Murf1* two weeks after NBPI (Nikolaou et al., 2019), it is conceivable there is a defined window for the upregulation of muscle-specific ubiquitin ligases after NBPI. Unfortunately, we do not have samples from the current experiments at earlier timepoints to assess the kinetics of these upstream ubiquitin ligases. Lastly, we have processed all available forelimb muscles for our various physiological and biochemical endpoints, and as such, do not have viable muscle samples for RNA extraction to determine the transcriptional activities of these ligases. We are unable to add these experiments to our manuscript, and have included a statement on the need for a more extensive investigation of ubiquitin ligases in future studies.

Results (lines #245-254): “Further investigation revealed that MSTN inhibition did not alter protein levels of the muscle-specific E3 ligases, *Murf1* and Atrogin-1, (Figure 6—figure supplement 1A-C). Unexpectedly, denervation decreased *Murf1* and did not change Atrogin-1 levels at 4 weeks post-NBPI, which differ from our earlier reports of increased transcriptional activity of *Murf1* at 2 weeks post NBPI.^13^ While there is a need to better characterize the precise kinetics of these upstream ubiquitin ligases over time following denervation, we show that downstream levels of ubiquitination are ultimately elevated with denervation. The relationship between ubiquitin ligase expression, ubiquitination, and proteasome activity in neonatally denervated muscle must be more extensively investigated.”

Discussion (lines #354-359): “Additionally, since we observe discrepancies in the temporal expression of upstream ubiquitin ligases in our current and prior studies,^13^ we wonder if there is a transient temporal window for the upregulation of muscle-specific ligases after NBPI. Thus, a more extensive investigation of ubiquitin ligase expression and function in future studies will provide a better of their role in neonatal denervation and muscle growth.”

Materials and methods (lines #678-690): “Membranes were subsequently blocked in 5% bovine serum albumin (BSA) in TRIS-buffered saline (TBS)-Tween (pH 7.5), and incubated overnight at 4°C with an antibody against puromycin (1:1000; MilliporeSigma #MABE343), K48-linkage-specific-polyubiquitin (1:1000, Cell Signaling #8081), *Murf1*

(1:1000; Abcam #ab183094), or Atrogin-1 (1:500; Invitrogen #PA5-19056). GAPDH (1:5000; Cell Signaling #2118) served as a control for sample loading. Membranes were then washed and incubated with IRDye 680CW anti-goat or 800CW anti-mouse IgG2a (1:5000; LI-COR Biosciences) orDyLight anti-rabbit (1:5000; Cell Signaling #5151) secondary antibodies. Following image detection on the Odyssey infrared detection system (LI-COR Biosciences), the relative abundance of puromycin incorporation (30 µg), K48-linked protein levels (30 µg), *Murf1* (10 µg), and Atrogin-1 protein levels (10 µg) were quantified using the Image Studio Lite program (LI-COR Biosciences), and normalized to corresponding GAPDH protein levels.^13,78^”

Figure Legends (lines #926-932): “Figure 6—figure supplement 1: Assessment of muscle-specific ubiquitin ligases. (A) Representative western blots and quantitative analyses of (B) *Murf1*, and (C) Atrogin-1 showed MSTN inhibition does not alter protein levels in denervated (NBPI) or contralateral triceps muscles. Data are presented as mean ± SD, n = 3 independent mice. (B), (C) 3-way ANOVA for sex, treatment, and denervation (repeated measures between forelimbs) with a Bonferroni correction for multiple comparisons. *P<0.05.”

– Figure 7: In order to characterize the Smad2/3 pathway, the authors investigated the Smad3 phosphorylation level. Due to prior literature in the field, the reviewer wonders why the authors did not assess Smad2 phosphorylation level, in order to get a fully explained picture of the situation. The reviewer suggests the authors provide this detailed information for a better comprehension of the hidden mechanisms.

We have performed western blots for Smad2 signaling, and updated the findings in our manuscript accordingly. Our results here show MSTN inhibition does not reduce Smad2 signaling in denervated muscles, adding to our speculation that MSTN targets the proteasome independent of the Smad2/3 pathway.

Results (lines #261-264): “Here, we observed sex-independent increases in both Smad2 and Smad3 phosphorylation and translation with neonatal denervation, which were not further altered with MSTN inhibition (Figures 7A-G).”

Materials and methods (lines #721-726): “Membranes were subsequently blocked in 5% BSA/TBS-Tween, and incubated overnight at 4°C with an antibody against phosphorylated Akt (Ser473) (1:750; Cell Signaling #9271), total Akt (1:750; Cell Signaling #9272), phosphorylated Smad2 (Ser465/467) (1:1000; Cell Signaling #3108), total Smad2 (1:1000; Cell Signaling #5339), phosphorylated Smad3 (Ser423, Ser425) (1:2000 Abcam #ab51451), or total Smad3 (1:2000; Abcam #ab28379).”

Materials and methods (lines #730): “Western blot signals were subsequently imaged and the relative abundance of phosphorylated and total protein levels of Akt, Smad2, and Smad3 were quantified as described above.”

Figure Legends (lines #872-882): “Figure 7: Sex-specific differences in MSTNmediated proteostasis dysregulation occurs independent of Smad2/3 signaling. (A) Representative western blots and quantitative analyses of (B) pSmad2, (C) total Smad2, (E) pSmad3, and (F) total Smad3 revealed that ACVR2B-Fc treatment does not blunt the denervation-induced increase in activity and translation of Smad2 and Smad3 in triceps muscles of both sexes. Quantification of the western signal for (D) pSmad2 and (G) pSmad3 normalized to total protein levels further indicated that MSTN inhibition does not alter Smad2/3 signaling in neonatally denervated muscles. n = 3-6 independent mice. Data are presented as mean ± SD. Statistical analyses: (B), (C), (D), (E), (F), (G) 3-way ANOVA for sex, treatment, and denervation (repeated measures between forelimbs) with a Bonferroni correction for multiple comparisons. *P<0.05, **P<0.01.”

– Lines 232-234: The authors state "Our collective findings thus posit a sex-specific role for MSTN in muscle proteostasis dysregulation after neonatal denervation through non-canonical signaling pathways."? While the reviewer agrees based on the data provided, the reviewer wonders why the authors did not investigate further in that direction. The reviewer wonders how the TAK1 pathway and its downstream targets such as JNK or p38 are regulated. The reviewer also wonders how the Ras pathway and its downstream targets such as ERK1/2 are regulated.

We thank the reviewer for these excellent suggestions. As noted in our response to the Public Review above, we will certainly be following up on our current findings by rigorously interrogating these pathways downstream of MSTN signaling in future studies. We have included a statement in our revised discussion to acknowledge this.

Discussion (lines #505-507): “Additional non-canonical alternatives downstream of MSTN signaling that should be further explored include *TAK1* and its downstream targets *JNK* and p38, as well as Ras and its downstream targets MEK1 and ERK1/2.”

– It is known that MSTN has inhibitory effects on the Wnt/β-catenin pathway, thereby blunting satellite cell proliferation. Besides Wnt/β-catenin signaling plays a crucial role in myoblast fusion. In this context of MSTN signaling inhibition, the reviewer wonders how this Wnt/β-catenin signaling is regulated and what sort of effect it can have on longitudinal muscle growth.

We have previously ruled out a role for satellite cell-mediated myonuclear accretion in longitudinal muscle growth (Nikolaou et al., 2009). Briefly, we found that satellite cellspecific genetic ablation of Myomaker, a muscle-specific fusogen, does not alter sarcomere length in both normally innervated and denervated muscles. Hence, we believe it is unlikely that MSTN signaling governs longitudinal muscle growth through modulation of satellite cell activity with the Wnt/β-catenin pathway. We apologize for omitting this prior finding, and have revised our Introduction to include this salient point.

Introduction (lines #86-87): “Such deficits in muscle length are independent of satellite cell-mediated myonuclear accretion, but are instead caused by aberrant levels of muscle protein degradation associated with increased catalytic activity of the 20S proteasome.^13^”

– Lines 245: The authors wrote "(Figures 8E-F)", but they forgot to call Figures 8G-H in the manuscript. The reviewer suggests revising it accordingly.

We apologize for this oversight. We have corrected this error and properly referenced the appropriate figures.

Results (lines #274-276): “Lastly, while bortezomib did not alter β5 subunit activity in either sex (Figures 8E-F), it attenuated β1 subunit activity at 8 and 12w only in male mice (Figures 8G-H).”

In the Discussion section:

– Line 272: The authors state "we pharmacologically targeted MSTN". While this is true, the authors cannot forget that it targets also the signaling mediated by Activin A. The reviewer thinks that it should appear in the whole manuscript.

The reviewer is correct that the ligand trap targets Activin A signaling, as well as other members of the TGF-β superfamily. As noted in our response to item #1 from Reviewer 1, we more clearly address the limitations of the ligand trap and how its broad specificity may affect the interpretation of our findings in our revised Discussion. While the ACVR2B-Fc decoy receptor binds to many members of the TGF-β superfamily, we have kept the focus of this extended discussion on Activin A.

Discussion (lines #479-481): “Indeed, the broad ligand specificity of ACVR2B-Fc increases the risk of toxicity to non-skeletal muscle tissues and cell types, ranging from epistaxis to telangiectasias.^69”^

Discussion (lines #511-531): “For a more nuanced interpretation of our findings, we must also consider the specificity of the ACVR2B-Fc decoy receptor as a ligand trap, as mentioned above. Previous studies have reported that many members of the TGF-β superfamily, such as Activin A and GDF-11, are capable of binding Activin Type IIB receptors.^74,75^ This broad array of targeted ligands makes the decoy receptor a potent inhibitor of not only MSTN, but also an inhibitor of various TGF-β family members that signal downstream of Activin binding. Of note, simultaneous inhibition of MSTN and Activin A leads to more effective muscle hypertrophy and force production in rodents and primates.^76^ Consequently, this ligand trap putatively elicits more robust increases in skeletal muscle growth than MSTN specific-agents.^19^ In our current study, it is possible that Activin A is differentially expressed and/or blocked with ACVR2B-Fc between sexes, which may account for the sex dimorphisms observed in neonatally denervated muscles. Indeed, a sex-specific role for Activin A in pancreatic ductal adenocarcinoma (PDAC)induced cachexia has recently been reported by Zhong et al. (2022). The authors observed that sex-specific differences in endogenous levels of Activin A contribute to sex dimorphisms in PDAC-induced cachexia, as well as the differential outcomes of ACVR2BFc treatment in attenuating tumor-induced Activin.^77^ Alternatively, we also wonder whether Activin A and/or other TGF-β family members are more acutely involved in the regulation of neonatal longitudinal muscle growth and contractures than MSTN. Future work with genetic models and expression profiles for the various TGF-β superfamily members are necessary to address these limitations in our current study.”

– Line 333: Please replace "NPBI" with "NBPI".

We apologize for this typographical error. We have corrected this acronym in our revised manuscript.

Discussion (line #399): “While we observed altered Akt/mTOR and Smad2/3 signaling with NBPI, which are corroborated by studies from other groups using different mouse denervation models,^55-57^ MSTN inhibition failed to impact these canonical pathways in female mice.”

– Lines 361-362 to 372: The authors state "These discrepancies might be attributable to sex-related differences of MSTN expression itself in skeletal muscles.". The reviewer wonders how the MSTN, Activin A, ACVR2, and ACVR2B expression is, in both females and males here. Also, the reviewer wonders what is the level of expression of MSTN, Activin A, and ACVR2 and ACVR2B expression in the Brachialis, Biceps, and Triceps. Could the authors imagine that there is also a muscle-dependent regulation?

We share the same questions regarding expression of these TGF-β superfamily members and their receptors in our neonatal muscles. In order for relevant conclusions to be drawn on potential sex-specific and muscle-dependent differences in expression, we would require an in-depth analysis of additional data from future studies. We have included a statement in our revised Discussion to reflect this point.

Discussion (lines #445-448): “Future studies should perform a comprehensive analysis of the expression of MSTN and other TGF-β superfamily members (such as Activin A), as well as the associated receptors (ACVR2 and ACVR2B) across sexes and muscle groups to account for potential sex- and/or muscle-dependent regulation.”

In a previous study, the authors looked at the effects of bortezomib over different periods (4, 8, and 12 weeks). Here, the authors studied the effects of ACVR2B-Fc treatment over a period of 4 weeks. The reviewer wonders why they did not repeat the same durations of experimentation. The reviewer thinks it would be helpful to have this detailed information for having a better comprehension of the observed effect.

Also, the reviewer wonders what the effect of combining bortezomib and ACVR2B-Fc treatment would be. What would the authors hypothesize?

We thank the reviewer for these excellent suggestions. As noted in our response to the Public Review above, we will certainly be following up on our current findings by integrating these pharmacological approaches and rigorously interrogating their longterm efficacy in preventing contractures.

The focus of this study is to establish a proof of concept that targeting a muscle-specific regulator of proteostasis (MSTN) can effectively prevent contractures during the critical window of neonatal muscle growth (4 weeks). As we have established that MSTN inhibition does prevent contractures in a sex-specific manner during this stage of development, ongoing studies are currently planned to build on our findings here by interrogating the efficacy of this pharmacological approach in preventing contractures at skeletal maturity and beyond (8 and 12 weeks). These studies also include combining bortezomib with ACVR2B-Fc over different periods, which conceptually, would optimize contracture prevention and truncate the window of necessary treatment in both sexes. Beyond this broad speculation, we would prefer not to discuss our specific hypotheses for these experiments in the manuscript.

Discussion (lines #532-541): “Finally, we cannot yet comment on the long-term efficacy of MSTN inhibition beyond four weeks following denervation. However, the focus of this study was to establish a proof of concept that targeting a muscle-specific regulator of proteostasis (MSTN) can effectively prevent contractures during the critical window of neonatal muscle growth (4 weeks). Furthermore, we have previously established that contracture formation plateaus at 4 weeks post-NBPI, making this time point ideal for such a proof of concept investigation. As we have established that MSTN inhibition does prevent contractures in a sex-specific manner during this stage of development, ongoing studies are currently planned to build on our findings here by interrogating the efficacy of this pharmacological approach in preventing contractures at skeletal maturity and beyond.”

In the Materials and methods section:

– Lines 456-458: The authors state "The binding of MSTN to the Fc domain inhibits MSTN from binding to Activin A, which blocks MSTN activity in the muscle". The reviewer thinks that it should be ACVR2B instead of Activin A. The reviewer suggests revising it.

We apologize for the misunderstanding and have revised this sentence accordingly.

Materials and methods (line #581): “The binding of MSTN to the Fc domain inhibits MSTN from binding to ACVR2B, which blocks MSTN activity in the muscle, ultimately leading to increased muscle protein synthesis and robust muscle growth.^20”^

– Line 495: Please replace "micro-computed tomography" with "micro-computed tomography (MicroCT)".

We have made the suggested revision.

Materials and methods (line #619): “micro computed tomography (MicroCT)”

– Line 550: Please replace "PVDF" with "polyvinylidene fluoride (PVDF)".

– Line 580: Please replace "SDS-PAGE" with "sodium dodecyl sulfate-polyacrylamide gel electrophoresis (SDS-PAGE)".

We have made the suggested revisions.

Materials and methods (lines #672-676): “Muscle homogenates were then heated at 65°C for 30 minutes, separated on 4-20% sodium dodecyl sulfate-polyacrylamide gel electrophoresis (SDS-PAGE) gels (10-30 µg of proteins), and transferred at 4°C to Immobilon-fluorescently labeled (Immobilon-FL) polyvinylidene fluoride (PVDF) membranes.”

– Line 553: Please replace "BSA" with "bovine serum albumin (BSA)".

We have made the suggested revision.

Materials and methods (line #679): “Membranes were subsequently blocked in 5% bovine serum albumin (BSA)”

– Lines 569-570: Please replace "β-1" with "β1".

– Line 570: Please replace "β-5" with "β5".

We have made the suggested revisions.

Materials and methods (lines #698): “The caspase-like activity of the 20S proteasome β1 catalytic subunit and chymotrypsin-like activity of the β5 catalytic subunit were assayed with 10 μg total protein per muscle”

– Line 598: Please replace "ANOVA" with "analysis of variance (ANOVA)".

We have made the suggested revision.

Materials and methods (lines #739-740): “For data sets with two independent variables (sex and treatment), a 2-way analysis of variance (ANOVA) with Bonferroni correction for multiple comparisons was performed.”

– Line 602: Please replace "SD" with "standard deviation (SD)".

We have made the suggested revision.

Materials and methods (line #743): “All data are presented as mean ± standard deviation (SD).”

– Line 613: Please replace "ARRIVE" with "Animal Research: Reporting of in vivo Experiments (ARRIVE)".

We have made the suggested revision.

Materials and methods (lines #755-756): “This study also adhered to the Animal Research: Reporting of in vivo Experiments (ARRIVE) 2.0 guidelines, and a checklist is provided with the manuscript.”

In the Figure Legends section:

– Line 650: Please replace "micro-CT" with "MicroCT".

– Lines 656-658: The reviewer feels that (E) and (H) are misplaced in the sentence "Despite this, (E), (H) the increases in muscle volume, muscle weight, and protein levels were larger in females than males when compared to their respective DPBS controls."

We thank the reviewer for bringing this to our attention. The original placement of (E) and (H) was indeed incorrect. We have corrected the placement to improve the readability of this sentence.

Figure Legends (lines #795-797): “Despite this, the increases in (E) muscle volume, and (H) muscle weight and protein levels were larger in females than males when compared to their respective DPBS controls.”
